# Supplementary material for: Geographic variation and factors associated with under-five mortality in Ethiopia. A spatial and multilevel analysis of Ethiopian mini demographic and health survey 2019
Source: PLoS One. 2022 Oct 13;17(10):e0275586. doi: 10.1371/journal.pone.0275586 (PMC9560495; doi:10.1371/journal.pone.0275586)

PLOS ONE

Geographic variation and factors associated with under-five mortality in Ethiopia. A spatial and multilevel analysis of Ethiopian Mini Demographic and Health Surveys 2019.

--Manuscript Draft--

**Manuscript Number:**	PONE-D-21-20589
**Article Type:**	Research Article
**Full Title:**	Geographic variation and factors associated with under-five mortality in Ethiopia. A spatial and multilevel analysis of Ethiopian Mini Demographic and Health Surveys 2019.
**Short Title:**	Geographic variation and factors associated with under-five mortality in Ethiopia
**Corresponding Author:**	Zemenu Tadesse Tessema, MSC Biostatistics<br>University of Gondar<br>Gondar, ETHIOPIA
**Keywords:**	Under five mortality; factors; spatial distribution; multilevel; Ethiopia
**Abstract:**	Background<br><br>The distribution of under-five mortality (U5M) worldwide is uneven and the burden is higher in Sub-Saharan African countries which account for more than 53% of the global under-five mortality. In Ethiopia, though U5M shows a substantial decrease from 1990 to 2019, it is still unacceptably high and unevenly distributed. Therefore, this study aimed to assess geographic variation and factors associated with under five mortality in Ethiopia.<br><br>Method<br><br>We sourced data from the most recent nationally representative 2019 Ethiopian mini Demographic and Health Survey data for this study. A sample size of 5,695 live births were considered for this study. Descriptive and analytical Analysis was conducted by using STATA version 16. Both multilevel and spatial analyses were employed to ascertain the factors associated with under-five mortality in Ethiopia.<br><br>Result<br><br>The under-five mortality is 5.9% with its 95% CI 5.4% to 6.6%. Based on the multivariable multilevel logistic regression model result; family size (AOR=0.09, 95%CI:(0.84, 0.99)), number of under-five children in the family (AOR=0.17, 95%CI: 0.14, 0.21), multiple birth (AOR=14.4, 95%CI: (8.5, 24.3)), Children who breastfed for less than 6 months (AOR=5.04, 95%CI: (3.81, 6.67)), People whose main roof is palm (AOR=0.57, 95%CI: 0.34, 0.96), Under-five children who are at birth order sixth or more (AOR=2.46, 95%CI: (1.49, 4.06)), institutional delivery (AOR=0.57, 95%CI: (0.41, 0.81)), being resident of Somali and Afar region (AOR=3.46, 95%CI: (1.58, 7.55)) and (AOR=2.54, 95%CI: (1.10, 5.85)) respectively were factors associated with under-five mortality. A spatial analysis result revealed that hot spot areas of under-five mortality were located in Somali region and Dire Dawa.<br><br>Conclusion<br><br>Under-five mortality in Ethiopia is 59 per 1000 live births which is high and unacceptable as compared to the 2030 agenda for sustainable development aimed at 25 per 1000 live births. Breast feed less than 6 month, twin birth, institutional delivery and high risk areas of under-five mortality (Somali and dire Dawa) were modifiable risk factors. Therefore maternal education on the advantage of breast feeding and institutional delivery is highly recommended. Women who deliver twins should be given special attention. An effective strategy should be designed for intervention for hot spot areas of under-five mortality that is Somali and Dire Dawa.
**Order of Authors:**	Zemenu Tadesse Tessema, MSC Biostatistics

Powered by Editorial Manager® and ProduXion Manager® from Aries Systems Corporation

	Tsion Mulat Tebeje
	Lewi Goytom Gebrehewet
**Additional Information:**	
**Question**	**Response**
**Financial Disclosure**<br><br>Enter a financial disclosure statement that describes the sources of funding for the work included in this submission. Review the submission guidelines for detailed requirements. View published research articles from *PLOS ONE* for specific examples.<br><br>This statement is required for submission and **will appear in the published article** if the submission is accepted. Please make sure it is accurate.<br><br>**Unfunded studies**<br>Enter: *The author(s) received no specific funding for this work.*<br><br>**Funded studies**<br>Enter a statement with the following details:<br>• Initials of the authors who received each award<br>• Grant numbers awarded to each author<br>• The full name of each funder<br>• URL of each funder website<br>• Did the sponsors or funders play any role in the study design, data collection and analysis, decision to publish, or preparation of the manuscript?<br>• **NO** - Include this sentence at the end of your statement: *The funders had no role in study design, data collection and analysis, decision to publish, or preparation of the manuscript.*<br>• **YES** - Specify the role(s) played.<br><br>* typeset	We didn’t receive external funds for this research.
**Competing Interests**<br><br>Use the instructions below to enter a competing interest statement for this submission. On behalf of all authors, disclose any competing interests that could be perceived to bias this work—acknowledging all financial support and any other relevant financial or non-	he authors have declared that no competing interests exist.

financial competing interests.<br><br>This statement is required for submission and **will appear in the published article** if the submission is accepted. Please make sure it is accurate and that any funding sources listed in your Funding Information later in the submission form are also declared in your Financial Disclosure statement.<br><br>View published research articles from *PLOS ONE* for specific examples.<br><br>**NO authors have competing interests**<br><br>Enter: *The authors have declared that no competing interests exist.*<br><br>**Authors with competing interests**<br><br>Enter competing interest details beginning with this statement:<br><br>*I have read the journal's policy and the authors of this manuscript have the following competing interests: [insert competing interests here]*<br><br>* typeset	
**Ethics Statement**<br><br>Enter an ethics statement for this submission. This statement is required if the study involved:<br><br>• Human participants<br>• Human specimens or tissue<br>• Vertebrate animals or cephalopods<br>• Vertebrate embryos or tissues<br>• Field research<br><br>Write "N/A" if the submission does not require an ethics statement.<br><br>General guidance is provided below. Consult the submission guidelines for	Ethical clearance was obtained from measure DHS through filling requesting a form for accessing data. The data used in this study are publicly available, aggregated secondary data that hasn't any personal identifying information that can be linked to study participants. The confidentiality of data was maintained anonymously.

detailed instructions. **Make sure that all information entered here is included in the Methods section of the manuscript.**<br><br>**Format for specific study types**<br><br>**Human Subject Research (involving human participants and/or tissue)**<br>• Give the name of the institutional review board or ethics committee that approved the study<br>• Include the approval number and/or a statement indicating approval of this research<br>• Indicate the form of consent obtained (written/oral) or the reason that consent was not obtained (e.g. the data were analyzed anonymously)<br><br>**Animal Research (involving vertebrate animals, embryos or tissues)**<br>• Provide the name of the Institutional Animal Care and Use Committee (IACUC) or other relevant ethics board that reviewed the study protocol, and indicate whether they approved this research or granted a formal waiver of ethical approval<br>• Include an approval number if one was obtained<br>• If the study involved *non-human primates*, add *additional details* about animal welfare and steps taken to ameliorate suffering<br>• If anesthesia, euthanasia, or any kind of animal sacrifice is part of the study, include briefly which substances and/or methods were applied<br><br>**Field Research**<br><br>Include the following details if this study involves the collection of plant, animal, or other materials from a natural setting:<br>• Field permit number<br>• Name of the institution or relevant body that granted permission	
**Data Availability**<br><br>Authors are required to make all data underlying the findings described fully available, without restriction, and from the time of publication. PLOS allows rare	Yes - all data are fully available without restriction

exceptions to address legal and ethical concerns. See the PLOS Data Policy and FAQ for detailed information.<br><br>A Data Availability Statement describing where the data can be found is required at submission. Your answers to this question constitute the Data Availability Statement and **will be published in the article**, if accepted.<br><br>**Important:** Stating 'data available on request from the author' is not sufficient. If your data are only available upon request, select 'No' for the first question and explain your exceptional situation in the text box.<br><br>Do the authors confirm that all data underlying the findings described in their manuscript are fully available without restriction?	
**Describe where the data may be found in full sentences. If you are copying our sample text, replace any instances of XXX with the appropriate details.**<br><br>• If the data are **held or will be held in a public repository**, include URLs, accession numbers or DOIs. If this information will only be available after acceptance, indicate this by ticking the box below. For example: *All XXX files are available from the XXX database (accession number(s) XXX, XXX.*).<br>• If the data are all contained **within the manuscript and/or Supporting Information files**, enter the following: *All relevant data are within the manuscript and its Supporting Information files.*<br>• If neither of these applies but you are able to provide **details of access elsewhere**, with or without limitations, please do so. For example:<br><br>*Data cannot be shared publicly because of [XXX]. Data are available from the XXX Institutional Data Access / Ethics Committee (contact via XXX) for researchers who meet the criteria for*	The datasets used during the current study are publicly available data from measure DHS program

access to confidential data.<br><br>*The data underlying the results presented in the study are available from (include the name of the third party and contact information or URL).*<br>• This text is appropriate if the data are owned by a third party and authors do not have permission to share the data.<br><br>* typeset	
Additional data availability information:	

Manuscript Click here to access/download;Manuscript;Final manuscript.docx

Geographic variation and factors associated with under-five mortality in Ethiopia. A spatial and multilevel analysis of Ethiopian Mini Demographic and Health Surveys 2019.

Zemenu Tadesse Tessema [1*], Tsion Mulat Tebeje[2], Lewi Goytom Gebrehewet[1]

[1] Department of Epidemiology and Biostatistics, Institute of Public Health, College of Medicine and Health Sciences, University of Gondar, Gondar, Ethiopia

[2] Epidemiology and Biostatistics unit, School of public health, Dilla university,Dilla, Ethiopia

E-mail : LGG lewigt369@gmail.com

TMT yemarina12@gmail.com

ZTT zemenut1979@gmail.com

***= Corresponding author**

Abstract

Background: The distribution of under-five mortality (U5M) worldwide is uneven and the burden is higher in Sub-Saharan African countries which account for more than 53% of the global under-five mortality. In Ethiopia, though U5M shows a substantial decrease from 1990 to 2019, it is still unacceptably high and unevenly distributed. Therefore, this study aimed to assess geographic variation and factors associated with under five mortality in Ethiopia.

Method: We sourced data from the most recent nationally representative 2019 Ethiopian mini Demographic and Health Survey data for this study. A sample size of 5,695 live births were considered for this study. Descriptive and analytical Analysis was conducted by using STATA version 16. Both multilevel and spatial analyses were employed to ascertain the factors associated with under-five mortality in Ethiopia.

Result: The under-five mortality is 5.9% with its 95% CI 5.4% to 6.6%. Based on the multivariable multilevel logistic regression model result; family size (AOR=0.09, 95%CI :(0.84, 0.99)), number of under-five children in the family (AOR=0.17, 95%CI: 0.14, 0.21), multiple birth (AOR=14.4, 95%CI: (8.5, 24.3)), Children who breastfed for less than 6 months (AOR=5.04, 95%CI: (3.81, 6.67)), People whose main roof is palm (AOR=0.57, 95%CI: 0.34, 0.96), Under-five children who are at birth order sixth or more (AOR=2.46, 95%CI: (1.49, 4.06)), institutional delivery (AOR=0.57, 95%CI: (0.41, 0.81)), being resident of Somali and Afar region (AOR=3.46, 95%CI: (1.58, 7.55)) and (AOR=2.54, 95%CI: (1.10, 5.85)) respectively were factors associated with under-five mortality. A spatial analysis result revealed that hot spot areas of under-five mortality were located in Somali region and Dire Dawa.

Conclusion: Under-five mortality in Ethiopia is 59 per 1000 live births which is high and unacceptable as compared to the 2030 agenda for sustainable development aimed at 25 per 1000 live births. Breast feed less than 6 month, twin birth, institutional delivery and high risk areas of under-five mortality (Somali and dire Dawa) were modifiable risk factors. Therefore maternal education on the advantage of breast feeding and institutional delivery is highly recommended. Women who deliver twins should be given special attention. An effective strategy should be designed for intervention for hot spot areas of under-five mortality that is Somali and Dire Dawa.

Keywords: Under five mortality, factors, spatial distribution, multilevel, Ethiopia

Background

Under-five mortality is defined as deaths reported at ages 0 to 59 months and it contains neonatal, post-neonatal, and child deaths (1). Under-five mortality rate (U5MR) is the probability of a child dying before celebrating fifth birthday (2). It is a significant indicator of the socioeconomic, health, environmental conditions, and national development of health equity and access (3).

Globally, in 1990, under-five deaths were 12,494,000 (93 deaths per 1000 live births). By showing an average annual decline of 3.1%, in 2019 it was significantly reduced to 5,189,000 deaths (38 deaths per 1000 live births) with a 58% reduction (4). The 2030 agenda for sustainable development is aimed at ending the preventable deaths of newborns and children under 5 years of age, with all countries aiming to reduce under-5 mortality to make it at least less than 25 per 1000 live births (5).

The distribution of U5MR worldwide is uneven and has wider disparity and the global burden of U5MR is concentrated at two sustainable development goals (SDG) regions: Sub-Saharan Africa and Central and Southern Asia with more than 80% of U5MR accounted for (6). The burden is higher in Sub-Saharan African countries with 74 deaths per 1000 live births, which accounts for more than 53% of the global under-five mortality (7).

In Ethiopia, U5MR shows a substantial decrease from 200 per 1000 live births it was in 1990 to 55 per 1000 live births in 2019 but still couldn't achieve the SDG aim (4). But a difference in administrative levels or regions and spatial variation is considerable. Afar, Somali, and Benishangul Gumuz are the three high risk regions of Ethiopia for U5MR (8). Different studies conducted in Ethiopia revealed several Socioeconomic, demographic, and geographic/spatial factors.

Different studies conducted on U5M previously showed that: source of water (9), multiple birth (7)(8), region (10)(11), age and sex of household head (10), maternal education status (12)(13), place of delivery (10)(11)(14), maternal age (15)(16)(17), sex of the child (9)(14)(16), residence (18)(19), family size (17)(11), number of children under five (18)(20), birth order (11)(18), and breast feeding status (18)(21) have a significant association with under-five mortality.

Though U5M has been declining it is still high and is taking the lives of many children, no research on U5M has been made on the recent survey data, Ethiopian mini Demographic Health Survey (EMDHS) 2019. Identifying areas and factors with the highest burden of U5M in Ethiopia is helpful to show the current burden of U5M, to make different policies and interventions of U5M prevention with the efficient allocation of scarce resources according to spatial variability based on our findings and to achieve the SDG by understanding the determinants. Therefore, this study is aimed at assessing the geographic variation and factors associated with Under-five mortality in Ethiopia.

Method

Data Source

Based on the 2019 EMDHS, which is the second mini demographic health survey (DHS) conducted in Ethiopia next to the 2014 EMDHS, secondary data analysis was accompanied. Kids Record (KR) format was the data of interest to conduct the study. The study took place in Ethiopia, which is a land-locked country located in the Horn of Africa that lies between the 30 N and 150N Latitude or 330 E and 480 E Longitude(22). Data collection was conducted from March 21, 2019, to June 28, 2019, from the nine regions (Tigray, Afar, Amhara, Oromia, Somali, Benishangul Gumuz, SNNPR, Harari, and Gambella) and two administrative cities (Addis Ababa and Dire Dawa). The study design was a population-based cross-sectional study. A frame of all census Enumeration areas (EAs) were used as a sampling frame for the 2019 EMDHS. 149,093 EAs were created which covers an average of 131 Households (HHS). A two-stage stratified cluster sampling technique was employed and each region was stratified into urban and rural areas, yielding 21 sampling strata. Enumeration areas (EAs) were selected independently in each stratum. In the first stage, 305 clusters (93 urban and 212 rural) were selected with probability proportional to EAs size and with independent selection in each sampling stratum. In the second stage, a fixed number of 30 households per cluster were selected. Finally, women aged 15–49 in 9150 (2790 urban and 6360 rural) households from 305 clusters were selected. The whole procedure of sampling is found in the full 2019 EMDHS report (23).

Study variables

The response variable was under-five mortality status, which was categorized as (child alive: Yes= 0 and No = 1). The age was recorded in months. Independent variables were classified as individual level and community level predictors (Place of residence, Region, Community place of delivery, Community wealth, Community media exposure, and Community toilet facility). The individual-level predictors were further categorized as socio-demographic and economic factors (Educational level of the mother, Sex of household head, age of household head, Number of household members, Number of child under the age of five, Marital status of the mother, Wealth index, Source of water, Time to get water, Type of toilet facility, Household electricity, Types of cooking fuel, Main floor material, Main wall material, Main roof material, and Media exposure) and Maternal and child factors (Maternal age at first birth, Sex of the child, Utilization of contraception, Order of birth, Mode of delivery, duration of breastfeeding and Multiple births).

Spatial analysis

The data for spatial analysis was cleaned by STATA version 16 and excel soft wares. ArcGIS version 10.8 and saTScan version 9.7 were used for the spatial analysis.

Spatial autocorrelation

Spatial autocorrelation (Global Moran's I) was conducted to see if under-five mortality was dispersed (Moran's I value closer to -1), clustered (Moran's I value closer to 1), or randomly distributed (Moran's I value of 0) in Ethiopia.

Spatial interpolation

Though all enumeration areas have known under-five mortality, areas that are not selected were predicted. Spatial interpolation was applied by using the geostatistical ordinary Kriging spatial interpolation technique to predict under-five mortality from a limited number of sample data points (EAs) to un-sampled areas.

Spatial scan statistics

Sat scan was used based on the Bernoulli test for cases (child is not alive) and controls (child is alive) to conduct the scan analysis. The upper limit used was the default maximum spatial cluster size of less than 50% of the population, allowing both small and large clusters to be detected, and clusters that contained more than the maximum limit with the circular shape of the window were avoided. Most likely clusters were identified using p-values and likelihood ratio tests, which is the ratio of the likelihood of the alternative hypothesis (higher activity level inside the window) over the likelihood of the null hypothesis (same activity level inside and outside).

Data management and analysis

We used STATA version 16 to analyze the data. Data (KR) was cleaned and 31 variables were kept that were needed for the analysis. Residents who are not De jure (included in the study if they lasted the night before the study) were excluded which affect under-five mortality, as they couldn't respond to most of the socio-demographic and economic characteristics though they can answer the maternal and child characteristics, which has changed our sample size from 5753 to 5695.

The outcome variable was re-coded to (child alive: Yes= 1 and No = 0). Generating of other variables, recording, renaming, and labeling was done. New 4 community variables were generated by taking the individual variable, calculating their proportion, and dichotomizing them based on their mean or median according to their distribution.

In the end, we had 29 predictor variables out of which 6 were community-level predictor variables and 23 individual-level predictor variables.

As we have a binary outcome variable (child alive = yes/no), to analyze factors associated with under-five mortality, binary logistic regression is appropriate. But based on EMDHS; respondents with the same cluster show similar findings or function at the same level and the data has a hierarchical structure. This makes binary logistic regression not to be used as it violates the assumption of independence of the residuals; rather a model which considers clustering effect should be used (8)(10). Multilevel logistic modeling separates the within-cluster effects from the between-cluster effects (24). Therefore, to assess the predictors associated with U5M, the multilevel logistic regression model was used.

Bivariable multilevel logistic regression was used for each predictor variable to screen out the significant variables (p-value less than 0.2) for multivariable multilevel logistic models. Twenty predictor variables (3 of them were community-level predictor variables) passed to multivariable analysis. In multivariable analysis, a p-value less than 0.05 was considered a factor associated with U5M.

The first model fitted was the null model (intercept model) which contains the outcome variable only (under-five mortality status) with the cluster number. The intra-cluster correlation (ICC) was used to assess whether there was a random effect. ICC of 0.130 which is not negligible that means there is a 13% under-five mortality which is explained by between cluster difference and around 87% of the under-five mortality is explained by within-cluster difference. The second model was fitted by using the outcome, the cluster and as a predictor variable the individual level only. The probability of U5M was predicted as a function of individual-level predictors.

For the third model the outcome variable, the cluster number, and the community level predictor variables were accounted for. Then the final model was fitted with both the individual level and the community level predictor variables taken into account.

The models were compared by using a log-likelihood statistic, where the fitted model had the smallest -2log * likelihood value that is deviance.

Ethical consideration

Permission for data access was obtained from a major demographic and health survey through an online request from http://www.dhsprogram.com. The data used for this study were publicly available with no personal identifier.

Result

Background socio-demographic and maternal and child health factor

An overall of 5,695 live births was encompassed in the study, of which 2,942 (51.66%) were male, 5,528 (97.07%) were single children, 2,937 (51.57%) were delivered at home and around 6.04% of the children were born via cesarean section (**Table 1**)

Out of the total children's mothers, most were married 5,346 (93.87 %) and 3,135 (55.05%) have no education, only 291(5.11%) attain higher education and Greater than 51.62% of the respondents are poor economic status. The majority of the respondents 64.27% are utilizers of improved water and 30.99 % travel more than 30 minutes, 50.85% travel less than 30 min to get water unlike those who have water on-premises. The majority of sex of household head is male 4,561 (80.09%) and the commonest age of household head is 35-50 with 45.58%.

Background of community factors (**Table 1**)

Among the population, the majority of the participants were rural residents with 4389(77.07%). And 3457 (60.70%) of the community practiced home delivery. The number of communities that utilize unimproved toilets is 3175(55.75%). 3391 of the population (59.54%) are not exposed to the media. And the majority of the children reside in rural areas (**Table 2**)

Prevalence of under-five mortality

The under-five mortality is 5.9% with its 95% (CI 5.4%, 6.6%) and it varies across regions ranging from 2.13% in Addis Ababa to 9.82 in the Somali region. Out of the total 918(15.79%) of children who had a history of lower than 6 months of breastfeeding died (**Table 3**)

Spatial autocorrelation

The global Moran's index was 0.09, z-score 3.5, p-value <0.001 suggesting that the spatial distribution under-five mortality is not at random in the 2019 EMDHS (**Figure 1**)

Hotspot analysis

A high proportion of U5M was observed around the regions of Somali and Dire Dawa (**Figure 2**)

Spatial Interpolation

The spatial interpolation or prediction analysis result showed that the under-five mortality ranges from 0% to 50%. The high risk predicted areas were located in Somali, Dire Dawa, Benishangul Gumuz, Gambella, and SNNPR (**Figure 3**)

Spatial scan statistics analysis

As observed in the figure 22 significant clusters were identified in the Somali region. Located within 7.384788 N, 45.908936 E with 612.75 km radius. Children living in the primary cluster were 62% more likely to die before they celebrate their fifth birthday when compared to those who lived outside the window (relative risk (1.62), Likelihood ratio (7.36), and p-value (0.03)) (**Figure 4**)

Factors associated with Under-five mortality

By using multilevel logistic regression, after filtering all variables in bivariable multilevel logistic regression, 4 models were fitted (null model, individual-level model, community-level model, and both individual and community level). The ICC was found to be 0.13 (95% CI: (0.08, 0.2)) which is not negligible. The 4th model was the best-fitted model with the lowest deviance. When the number of household members increases by one, the odds of under-five mortality will be decreased by 8.5% (AOR=0.09, 95%CI: (0.84, 0.99)). The odds of under-five death will be decreased by 82.8% (AOR=0.17, 95%CI: 0.14, 0.21) as the number of under-five children in the family increases by one. Children who have multiple births have 14 times higher odds of under-five mortality (AOR=14.4, 95%CI: (8.5, 24.3)) than singletons. Children who breastfed for less than 6months (AOR=5.04, 95%CI: (3.81, 6.67)) have 5 times higher odds of experiencing under-five mortality than those who have breastfed. People whose main roof is palm/bamboo have 43% (AOR=0.57, 95%CI: (0.34, 0.96)) decreased odds of having under-five deaths in the family than that of corrugated iron. Under-five children who are in the sixth birth order and more are 2.5 (AOR=2.46, 95%CI: (1.49, 4.06)) times higher odds to have under-five mortality as compared with second and third birth order. Place of delivery was found to have a significant association with U5M, those who have delivered in institutions have 43% less chance of experiencing U5M (AOR=0.57, 95%CI: (0.41, 0.81)) as compared with those delivered at home. Being a resident of the Somali and Afar region increases the odds of under-five death by 3.5 (AOR=3.46, 95%CI:

(1.58, 7.55)) and 2.5 (AOR=2.54, 95%CI: (1.10, 5.85)) times respectively as compared with Tigray region (**Table 4**).

Discussion

The study mainly focused on the spatial distribution of under-five mortality and on factors that influence it. The findings of this study revealed that the prevalence of under-five mortality was 59 per 1000 live births with (95% CI: (54, 66)). The multivariable multilevel logistic regression model result showed that family size, number of under five children, birth order, breast feed, multiple birth, region and institutional delivery were associated with under-five mortality in Ethiopia. The spatial analysis result revealed that Somali and Dire Dawa were high risk areas for under five mortality in Ethiopia.

The study showed that under-five mortality in Ethiopia is 59 per 1000 live births with (95% CI: (5.4%, 6.6%)). This finding is not consistent with studies conducted in Ethiopia (8)(19), this difference might be due to the survey time of the data used and the sample size.

Increased number of households (family size) resulted in decreased odds of under-five death. It is in line with a similar study in Ethiopia (17), and Eastern Nigeria (25). This can be explained by, as the family size increases, the mother of the children will be more experienced in taking good and adequate care of the children. As the number of household members is increasing, many people can care for the under-five children with fewer burdens.

As the number of under-five children in a family increases, the odds of under-five mortality will be decreased. This is consistent with a study in Bangladesh (26) and Ghana (26). This is because the mother will be more experienced and she will learn from her previous mistakes if there is one. But there is a contradiction with another study result in Ethiopia, which demonstrated that the odds of under-five mortality increases as the number of under-five children increases (18). This can be justified as the number of under-five children increases: it will be difficult to fulfill their basic needs and their family (mother) will be exhausted to look after the children.

According to our study results being fourth and fifth or sixth and above-born children increases the odds of under-five mortality. This is in line with a study conducted in rural parts of Ethiopia (11). But it is not supported by a study conducted in Ethiopia which pointed out that being firstborn increases the odds of under-five mortality by two folds when compared to those with five and above birth orders(18). This can be because firstborns are more likely to be hospitalized as a result of congenital malformations and perinatal conditions in their early childhood. But, this changes for firstborn children as they get older, when younger brothers and sisters are more likely to be hospitalized for injuries and avoidable conditions, which shows less parental care (27).

Breastfeeding less than six months increases the odds of under-five mortality by 6.78 fold as compared with those who breastfed for six and more months. Which is consistent with a finding in Ethiopia (18). This can be justified by, breastfeeding is one of the prevention methods for reducing child mortality and it is a means of prevention of delayed growth too (28).

Being multiple birth children increases the odds of under-five mortality by fourteen times that of singleton children. This finding is in line with different studies conducted in Ethiopia at different times, a study conducted in rural parts of Ethiopia (11)(16)(7)(8)(18). This can be due to multiple birth imposes short and long term medical complications (29).

The odds of under-five mortality are higher for residents of Afar and Somali regions by 2.5 and 3.5 times respectively as compared to the Tigray region. Somali and Afar have the first and second lowest coverage of children with all basic vaccinations. Children under five with symptoms of Acute Respiratory Infection and diarrhea are below the national average. There is serious child malnutrition with the first and second highest prevalence of wasting for Somali and Afar respectively (30)(31). This is not supported by studies conducted in Ethiopia based on the 2011 and 2016 EDHS (9)(10)(11) where other regions are found to have a significant association with under-five mortality. This discrepancy can be a result of the different number of enumeration areas (clusters) which led to different samples, different statistical models used for analysis (hurdle negative binomial mode1, multilevel negative binomial regression model, and binary logistic regression).

Facility or institutional delivery reduces the odds of under-five mortality by 43% as compared to home delivery. This finding is consistent with studies conducted in Ethiopia (10)(11) and the

Democratic Republic of Congo (14). This is because; Home delivery increases the death of under-five children due to a higher risk of postpartum complications after the intrapartum, exposure to common vaccine-preventable diseases as a result of an absence of vaccination, and less likely to attend prenatal and postpartum visits (10)(32).

Conclusion: Under-five mortality in Ethiopia is 59 per 1000 live births which is high and unacceptable as compared to the 2030 agenda for sustainable development aimed at 25 per 1000 live births. Breast feed more than 6 month, twin birth, institutional delivery and high risk areas of U5M (Somali and dire Dawa) were modifiable risk factors. Therefore maternal education on the advantage of breast feeding and institutional delivery is highly recommended. Women who deliver twin should be given special attention. An effective strategy should be designed for intervention for hot spot areas of U5M that is Somali and Dire Dawa.

Strength and limitation of the study

We used EMDHS 2019 data which is more inclusive and generalizable as EAs are taken from most parts of the country. The larger sample size increases the power of the study and helps to make geographical comparisons. As it is cross-sectional it does not show a temporal relationship (chicken egg dilemma), important variables: antenatal care follow-up, postnatal care, and preceding birth interval were excluded from the study due to their highest rate of missing values.

Abbreviation

AOR: Adjusted Odds Ratio, CI: Confidence Interval, CS: Cesarean Section, EAs: Enumeration Areas, DHS: Demography and Health Survey, EMDHS: Ethiopia Mini Demography and Health Survey, HHs: households, ICC: Intraclass Correlation, KR: kids record, SGD: Sustainable Development Goal, U5M: under-five mortality, U5MR: under-five mortality rate

Declaration

Ethics approval and consent to participate

Ethical clearance was obtained from measure DHS through filling requesting a form for accessing data. The data used in this study are publicly available, aggregated secondary data that hasn’t any personal identifying information that can be linked to study participants. The confidentiality of data was maintained anonymously.

Availability of data and materials

The datasets used during the current study are publicly available data from measure DHS program

Funding

We didn't receive external funds for this research.

Acknowledgment

We acknowledge the DHS program for providing us the dataset.

Consent for publication

Not applicable as there is no image or other confidentiality related issues.

Competing interests

The authors declared that they have no competing interests.

Authors' contributions

LGG, TMT and ZTT conceived the study, data analysis, drafted the manuscript, and critically reviewed the manuscript. Read and approve the final version of the manuscript.

References

1. Croft, Trevor N., Aileen M. J. Marshall, Courtney K. Allen, et al. Guide to DHS Statistics. Rockville, Maryland, USA: ICF Guide. 2018;22–51.

2. World Health Organization. Proposed working definition of an older person in Africa for the MDS Project. Zagreb International Review of Economics & Business. 2018;18(1):1–15.

3. Woldeamanuel BT, Aga MA. Count Models Analysis of Factors Associated with Under-Five Mortality in Ethiopia. Global Pediatric Health. 2021 Jan;8:2333794X2198953.

4. UN Inter-agency Group for Child Mortality. Levels and Trends in Child Mortality: Report 2020: Estimates Developed by the Un Inter-Agency Group for Child Mortality Estimation. Report 2020; 2020.

5. Johnston R. Arsenic and the 2030 Agenda for Sustainable Development. In: Arsenic in the Environment - Proceedings [Internet]. CRC Press; 2016 [cited 2021 Jun 20]. p. 12–4. Available from: http://dx.doi.org/10.1201/b20466-7

6. Li Z, Hsiao Y, Godwin J, Martin BD, Wakefield J, Clark SJ. Changes in the spatial distribution of the under-five mortality rate: Small-area analysis of 122 DHS surveys in 262 subregions of 35 countries in Africa. PLOS ONE. 2019 Jan 22;14(1):e0210645.

7. Fenta SM, Fenta HM, Ayenew GM. The best statistical model to estimate predictors of under-five mortality in Ethiopia. Journal of Big Data. 2020 Aug 17;7(1).

8. Worku MG, Teshale AB, Tesema GA. Determinants of under-five mortality in the high mortality regions of Ethiopia: mixed-effect logistic regression analysis. Archives of Public Health. 2021 Apr 23;79(1).

9. Ayele DG, Zewotir TT, Mwambi HG. Structured additive regression models with spatial correlation to estimate under-five mortality risk factors in Ethiopia. BMC Public Health. 2015 Mar 19;15(1).

10. Geremew BM, Gelaye KA, Melesse AW, Akalu TY, Baraki AG. Factors Affecting Under-Five Mortality in Ethiopia: A Multilevel Negative Binomial Model. Pediatric Health, Medicine and Therapeutics. 2020 Dec;Volume 11:525–34.

11. Gebremichael SG, Fenta SM. Under-Five Mortality and Associated Risk Factors in Rural Settings of Ethiopia: Evidence from 2016 Ethiopian Demographic and Health Survey. Advances in Public Health. 2020 Aug 31;2020:1–13.

12. Singh A, Masquelier B. Continuities and changes in spatial patterns of under-five mortality at the district level in India (1991–2011). International Journal of Health Geographics. 2018 Nov 15;17(1).
13. Ghilagaber G, Antai D, Kandala N-B. Modeling Spatial Effects on Childhood Mortality Via Geo-additive Bayesian Discrete-Time Survival Model: A Case Study from Nigeria. In: Advanced Techniques for Modelling Maternal and Child Health in Africa [Internet]. Dordrecht: Springer Netherlands; 2013 [cited 2021 Jun 20]. p. 29–48. Available from: http://dx.doi.org/10.1007/978-94-007-6778-2_3
14. Kandala N-B, Mandungu TP, Mbela K, Nzita KP, Kalambayi BB, Kayembe KP, et al. Child mortality in the Democratic Republic of Congo: cross-sectional evidence of the effect of geographic location and prolonged conflict from a national household survey. BMC Public Health. 2014 Mar 20;14(1).
15. Anafcheh T, Yaghoubi Doust M, Mojadam M, Mirkazemi R, Khafaie MA. Temporal and spatial distribution of under-five mortality and factors associated with multiple cases of under-five deaths within a family in the rural area of Khuzestan, Southern Iran. Scientific Reports. 2018 Dec;8(1).
16. Dejene T, Girma E. Social determinants of under-five mortality in Ethiopia: Event history analysis using evidence from Ethiopian Demographic and Health Survey (EDHS). Health. 2013;05(05):879–84.
17. Ayele DG, Zewotir TT. Childhood mortality spatial distribution in Ethiopia. Journal of Applied Statistics. 2016 Feb 18;43(15):2813–28.
18. Zewudie AT, Gelagay AA, Enyew EF. Determinants of Under-Five Child Mortality in Ethiopia: Analysis Using Ethiopian Demographic Health Survey, 2016. International Journal of Pediatrics. 2020 Sep 22;2020:1–9.
19. Fikru C, Getinet M, Shaweno T. Proximate Determinants of Under-Five Mortality in Ethiopia: Using 2016 Nationwide Survey Data. Pediatric Health, Medicine and Therapeutics. 2019 Dec;Volume 10:169–76.
20. Abir T, Agho KE, Page AN, Milton AH, Dibley MJ. Risk factors for under-5 mortality: evidence from Bangladesh Demographic and Health Survey, 2004–2011. BMJ Open. 2015 Aug;5(8):e006722.

21. Pretorius CE, Asare H, Genuneit J, Kruger HS, Ricci C. Impact of breastfeeding on mortality in sub-Saharan Africa: a systematic review, meta-analysis, and cost-evaluation. European Journal of Pediatrics. 2020 Jun 26;179(8):1213–25.

22. Country profile: Croatia. 2019 Jun 18;

23. Ethiopian Public Health Institute (EPHI) [Ethiopia] and ICF. Ethiopia Mini Demographic and Health Survey 2019: Final Report. Rockville, Maryland, USA: EPHI and ICF; 2021.

24. Sommet N, Morselli D. Keep Calm and Learn Multilevel Logistic Modeling: A Simplified Three-Step Procedure Using Stata, R, Mplus, and SPSS. International Review of Social Psychology. 2017 Sep 8;30(1):203–18.

25. Azuike EC. Determinants of Under-Five Mortality in South-Eastern Nigeria. Community Medicine and Public Health Care. 2019 Jul 10;6(2):1–6.

26. Aheto JMK. Predictive model and determinants of under-five child mortality: evidence from the 2014 Ghana demographic and health survey. BMC Public Health. 2019 Jan 14;19(1).

27. Björkegren E, Svaleryd H. Birth Order and Child Health . Institute for Evaluation of Labour Market and Education Policy (IFAU). 2017(16).

28. WHO UNICEF IBFAN. (2016). Marketing of Substitutes : National Implementation of the International Code Status Report. 2016.

29. Elster N. Less is more: the risks of multiple births. Fertility and Sterility. 2000 Oct;74(4):617–23.

30. UNICEF Ethiopia. Regional Situation Analysis of Children and Women: Afar Region . 2019 Dec.

31. UNICEF Ethiopia. Regional Situation Analysis of Children and Women: Somali Region. 2019 Dec.

32. Goyal S, Kortsmit K, Cox S, D'Angelo DV, Romero L, Henderson ZT, et al. Prevalence of Home Births and Associated Risk Profile and Maternal Characteristics, 2016–2018. Obstetrics & Gynecology. 2020 Nov 4;136(6):1195–203.

List of Figures

Figure 1 Spatial autocorrelation of under-five mortality in Ethiopia 2019.

Figure 2 Hotspot analysis of under-five mortality in Ethiopia 2019.

Figure 3 Empirical Bayesian Kriging prediction map of under-five mortality in Ethiopia 2019.

Figure 4 Spatial scan statistics of under-five mortality in Ethiopia 2019.

Table 1 Sociodemographic, maternal, and child health factors

Sociodemographic, Maternal and child health factors		Frequency	Percentage
Age of the mother at first birth	Less than 18	3,024	53.10%
	19 upto 34	2,650	46.53%
	35 and more	21	0.37%
Sex of child	Male	2,942	51.66%
	Female	2,753	48.34%
Contraceptive utilization	not use	3,875	68.04%
	use	1,820	31.96%
Multiple births	Singleton	5,528	97.07%
	Multiple	167	2.93%
Birth order	1st order	1,228	21.56%
	2nd and 3rd order	1,883	33.06%
	4th and 5th order	1,298	22.79%
	6th order	1,286	22.58%
Place where the mother delivered	home delivery	2,937	51.57%
	Facility delivery	2,758	48.43%
Delivery by cesarean section	No	5,351	93.96%
	yes	344	6.04%
Months of Breastfeed	Less than 6month	918	16.12%
	6 month and grater	4,777	83.88%
	Male	4,561	80.09%

Sex of household head	Female	1,134	19.91%
Age of household head	less than 34	2,479	43.53%
	35 upto 50	2,596	45.58%
	50 and more	620	10.89%
Marital Status	Married	5,346	93.87%
	Unmarried	349	6.13%
Highest educational level	No education	3,135	55.05%
	Primary	1,802	31.64%
	Secondary	467	8.20%
	Higher	291	5.11%
Wealth index	Poor	2,940	51.62%
	Middle	797	13.99%
	Rich	1,958	34.38%
Water source	Improved	3,660	64.27%
	Unimproved	2,035	35.73%
Time to fetch water	more than 30 min	1,765	30.99%
	less than 30 min	2,896	50.85%
	On premises	1,034	18.16%
Type of latrine used	Improved	1,164	20.44%
	Not improved	4,531	79.56%
Household has electricity	No	4,073	71.52%
	Yes	1,622	28.48%
Cooking fuel in use	Clean fuel	432	7.59%
	Solid fuel	5,263	92.41%
Type of floor of the house	Earth/sand/dung	4,384	76.98%
	Wood	49	0.86%
	Cement	799	14.03%
	Carpet	463	8.13 %

Type of the house wall	Cane/Palm/Trunk/Reed	1,261	22.14%
	Bamboo/wood	3,531	62.00%
	Cement/stone with lime/bricks/covered adobe	903	15.86%
Type of the house roof	Thatch	1,488	26.13%
	Palm/bam	995	17.47%
	Cement	198	3.48%
	corrugated iron	3,014	52.92 %

Table 2 Community-level factors

Community level factors		frequency	Percentage
Place of residence	Urban	1,306	22.93%
	Rural	4,389	77.07%
Community place of delivery	Home delivery	3,457	60.70%
	Facility delivery	2,238	39.30%
Community toilet utilization	Improved	2,520	44.25%
	Not improved	3,175	55.75%
Community wealth status	Poor	2,776	48.74%
	Rich	2,919	51.26%
Community media exposure	Not exposed to media	3,391	59.54%
	Exposed to media	2,304	40.46%

Table 3 Under-five mortality among predictors

Characteristics		Is the child alive?		total
		Yes 5357(94.06%)	No 338(5.94%)	
Is child twin?	Yes	123	44	167
	No	5234	294	5,528
Region	Tigray	436	14	450
	Afar	616	35	651
	Amhara	478	23	501
	Oromia	674	41	715
	Somali	580	57	637
	Benishangul	477	45	522
	SNNPR	631	26	657
	Gambella	409	35	444
	Harari	408	29	437
	Addis Ababa	281	6	287
	Dire Dawa	367	27	394
Breastfeeding	Less than 6 months	773	145	918
	6 months and more	4,584	193	4,777
Birth order	1st order	1,142	86	1,228
	2-3rd order	1,793	90	1,883
	4-5th order	1,231	67	1,298
	6th + order	1,191	95	1,286
Place of delivery	Home delivery	2,733	204	2,937
	Facility delivery	2,624	134	2,758

Table 4 Multivariable multilevel logistic regression analysis of both individual and community-level factors associated with under-five mortality in Ethiopia, EMDHS 2019.

Variable	Null model	Model2 AOR(95%CI)	Model3 AOR(95%CI)	Model4 AOR(95%CI)
Education				
No education		1		1
Primary		1.14 (0.83,1.56)		1.14 (0.83,1.58)
Secondary		0. .59 (0.31, 1.13)		0.58(0.30,1.12)
Higher		0. .65(0. .28, 1.55)		0.60(0.25,1.44)
Household members		0.922(0. 85, 1.01)		0.92(0.83,0.99) *
Number of under-five children		0.17 (0.14, 0 .22)		0.17(0.14, 0.21) ***
Sex				
Male		1		1
female		0 .83(0 .64, 1.07)		0.84(0.64,1.09)
Mode of delivery by C/S				
No		1		1
Yes		1.36 (0. 79, 2.35)		1.37(0.79,2.39)
Preceding birth order				
1st		1		1
2-3rd		1.32(0.92, 1.91)		1.34(0.93,1.94)

4-5th		1.52(0.97, 2.39)		1.51(0.96,2.38)
6+		2.44(1.48, 4.02)		2.46(1.49,4.06) ***
Time to water				
>30min		1		1
<30min		0.77(0.56, 1.04)		0.89(0.64,1.23)
On-premises		0.66(0.38, 1.14)		0.76(0.43,1.34)
Cooking fuel				
Clean fuel		1		1
Solid fuel		1.27(0.62, 2.58)		0.93(0.45,1.94)
Age at 1st birth				
19-34		1		1
<=18		0.91(0.70, 1.20)		0.90(0.68,1.18)
>=35		2.58(0.55, 12.01)		2.72(0.55,13.59)
Breastfeeding				
< 6 months		1		1
>= 6 months		5.09(3.85, 6.72)		5.05(3.82,6.67) ***
Place of delivery				
Home delivery		1		1
Facility delivery		0.54 (0.40,0.75)		0.57(0.41,0.81) **
Main floor				
earth/sand/dung		1		1
Wood		1.71(0.53, 5.52)		2.14(0.66,6.95)
Cement		0.89(0.50, 1.59)		0.86(0.48,1.58)
Carpet		1.56(0.85, 2.86)		1.28(0.67,2.46)
Main wall				
bamboo/wood		1		1

cane/palm/trunks/reed		1.42(0 .97, 2.07)		1.06(0.70,1.58)
cement/stone		1.53(0.89,2.61)		1.34(0.76,2.34)
Main roof				
Corrugated iron		1		
thatch		1.25(0.87,1.79)		1.14(0.77,1.67)
palm/bamboo		0.71(0.44,1.15)		0.57(0.34,0.96)*
calamine/cement		1.14(0.54,2.44)		1.04(0.47,2.29)
Contraceptive utilization				
No		1		1
Yes		0.72(0.53,0.98)		0.82(0.60,1.13)
Marital status				
Currently Married		1		1
Currently Unmarried		1.19(0.74,1.92)		1.22(0.75,1.97)
Multiple Twin child				
No		1		1
Yes		15.63(9.33,26.20)		14.39(8.51,24.33)***
Region				
Tigray			1	1
Afar			1.60(0.76,3.41)	2.55(1.10,5.85)*
Amhara			1.47(0.68,3.19)	0.97(0.44,2.15)
Oromia			1.73(0.84,3.55)	1.52(0.73,3.15)
Somali			2.86(1.39,5.89)	3.46(1.59,7.55)**

Benishangul gumuz			2.74(1.32,5.68)	1.95(0.91,4.20)
SNNPR			1.17(.55,2.47)	0.94(0.44,2.03)
Gambella			2.71(1.29,5.69)	1.94(0.90,4.21)
Harari			2.37(1.12,5.04)	1.95(0.87,4.36)
Addis Ababa			0.71(0.25,2.03)	0.64(0.21,2.02)
Dire Dawa			2.18(1.01,4.70)	2.02(0.91,4.50)
Community delivery place				
Home delivery			1	1
Facility delivery			0.75(0.52, 1.09)	0.94(0.62,1.43)
Community media				
Not exposed			1	1
exposed			1.09(0.75,1.58)	0.94(0.64,1.40)
Intercept	0.49(0.30,0.81)	0.22(0.08,0.63)	0.35(0.19,0.64)	0.16(0.04,0.57)
Log-likelihood	-1263.56	-918.15	-1247.40	-903.72
Deviance	2527.12	1836.30	2494.80	1807.44

*** P-value<0.001, ** p-value<0.01, * p-value<0.05

Figure

Click here to access/download;Figure;Figure 1.tif

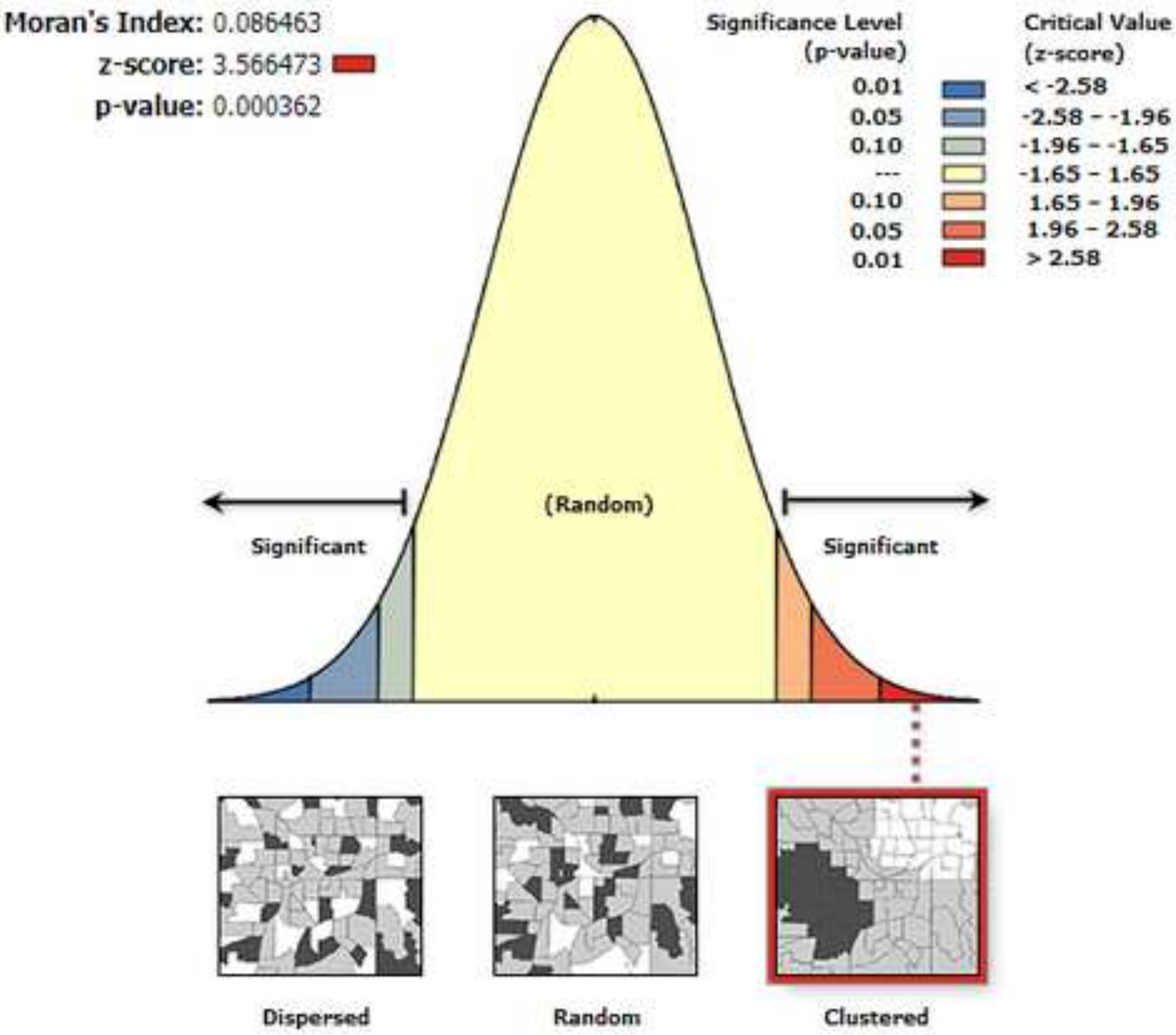

Figure
Click here to access/download;Figure;Figure 2.tif

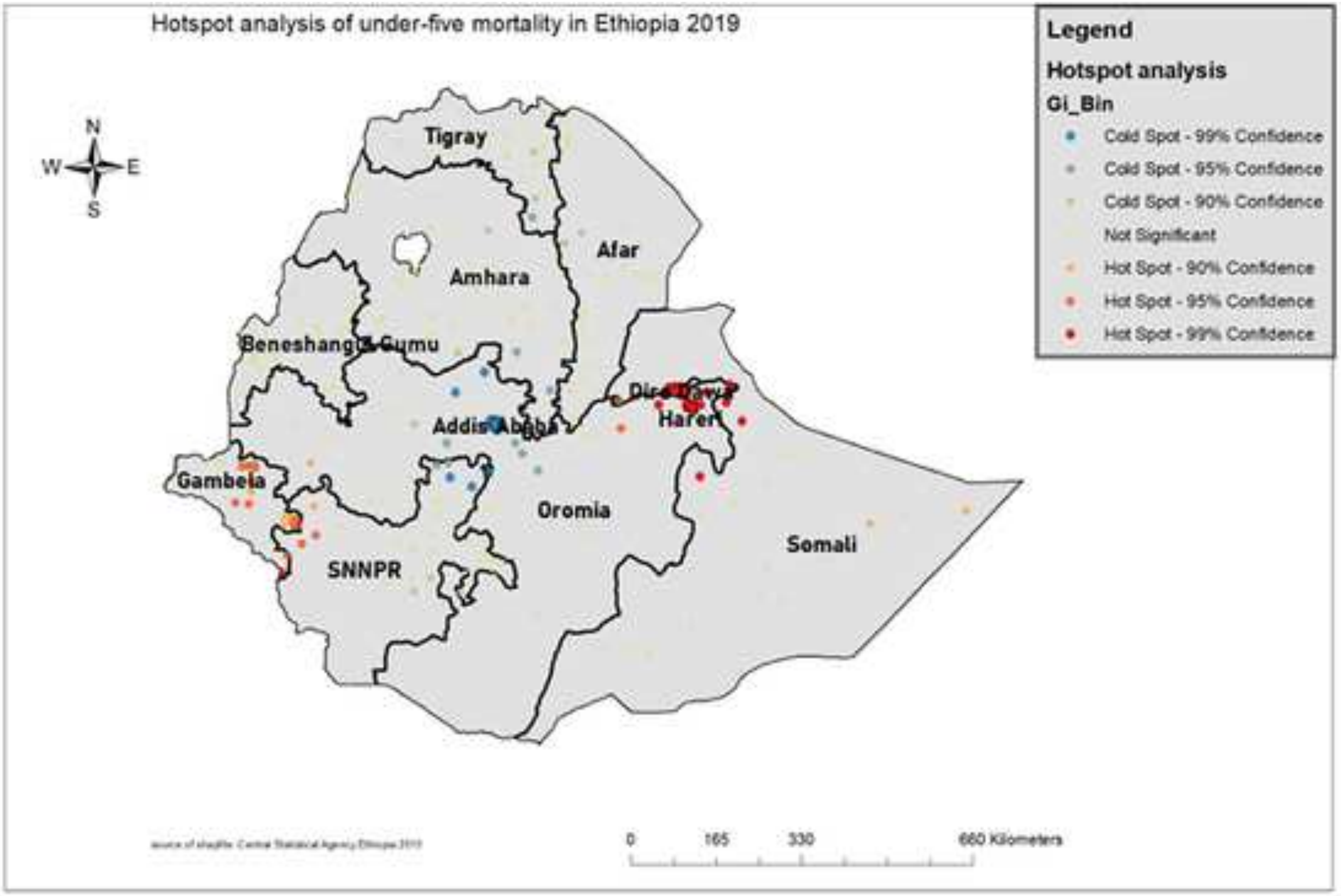

Click here to access/download;Figure;Figure 3.tif

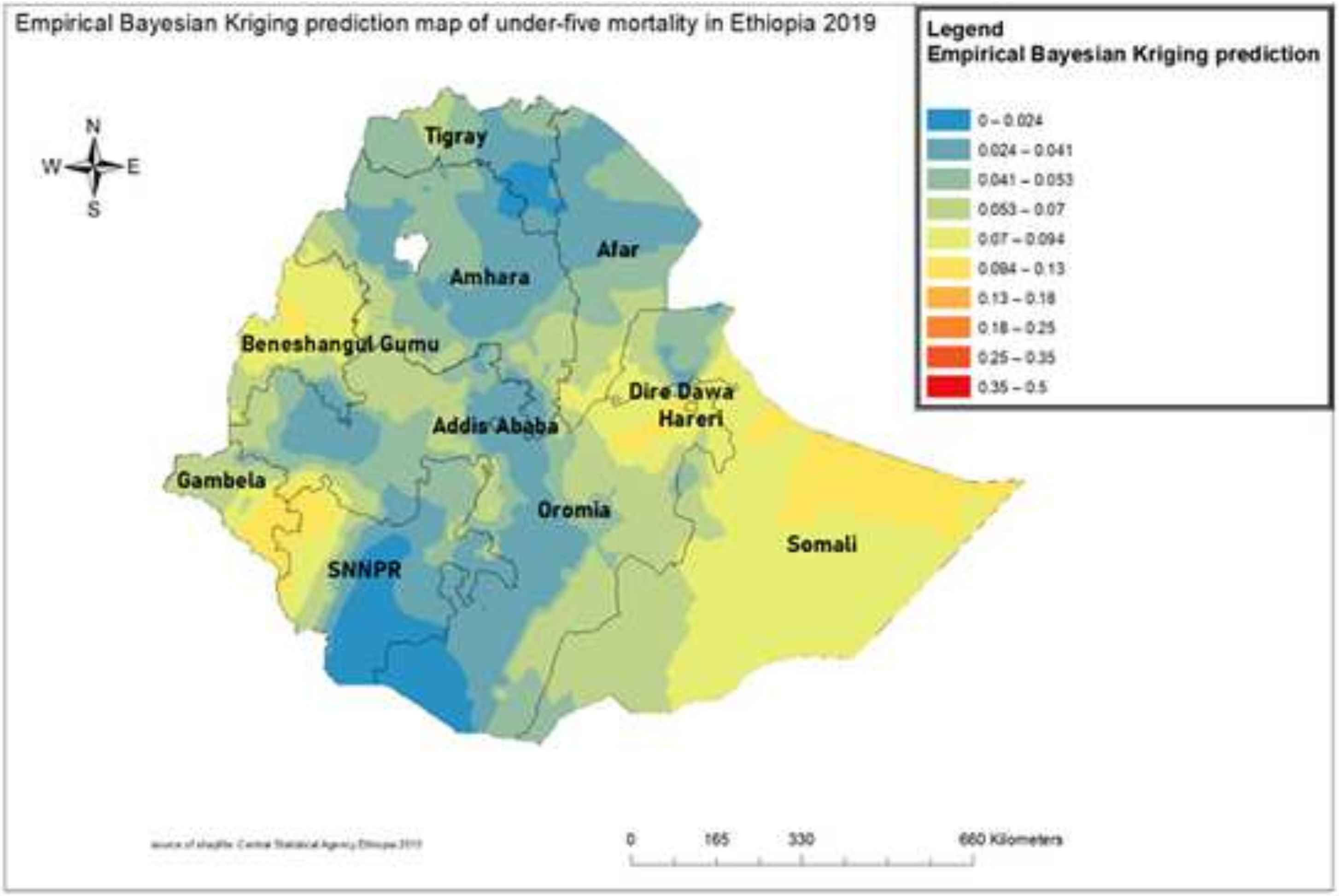

Figure

Click here to access/download;Figure;Figure 4.tif

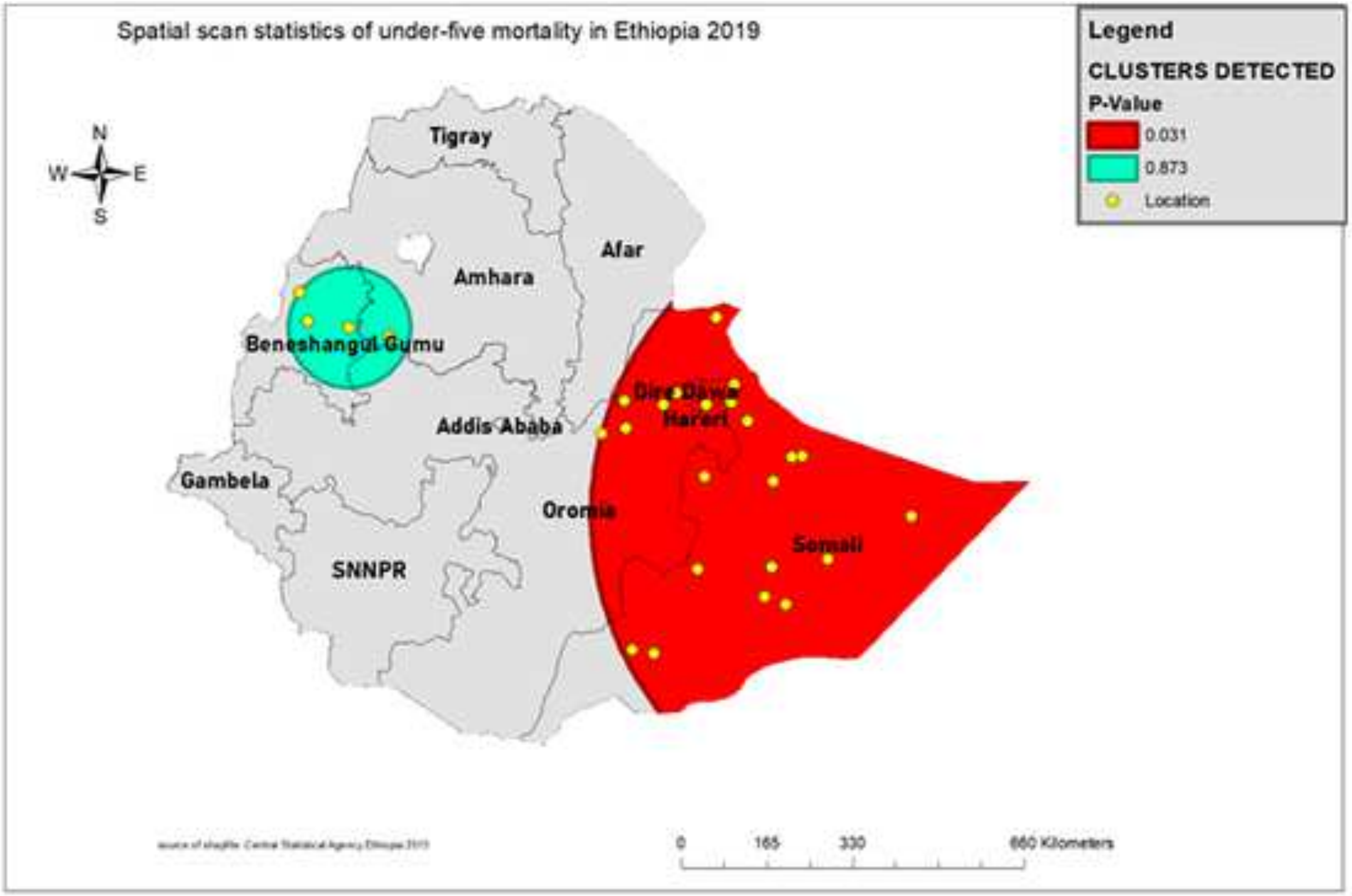

Supplement: Attachment — Submitted filename: PONE-D-21-20589_reviewer.pdf [file pone.0275586.s001.pdf]
