# Supplementary material for: Geographic variation and factors associated with under-five mortality in Ethiopia. A spatial and multilevel analysis of Ethiopian mini demographic and health survey 2019
Source: PLoS One. 2022 Oct 13;17(10):e0275586. doi: 10.1371/journal.pone.0275586 (PMC9560495; doi:10.1371/journal.pone.0275586)
Supplement: Attachment — Submitted filename: PONE-D-21-20589_AF.pdf [file pone.0275586.s004.pdf]

PLOS ONE

Geographic variation and factors associated with under-five mortality in Ethiopia. A spatial and multilevel analysis of Ethiopian Mini Demographic and Health Surveys 2019.

--Manuscript Draft--

Manuscript Number: PONE-D-21-20589R1

Article Type: Research Article

Full Title: Geographic variation and factors associated with under-five mortality in Ethiopia. A spatial and multilevel analysis of Ethiopian Mini Demographic and Health Surveys 2019.

Short Title: Geographic variation and factors associated with under-five mortality in Ethiopia

Corresponding Author: Zemenu Tadesse Tessema, MSC Biostatistics
University of Gondar
Gondar, ETHIOPIA

Keywords: Under five mortality; factors; spatial distribution; multilevel; Ethiopia

Abstract:

Background

The distribution of under-five mortality (U5M) worldwide is uneven and the burden is higher in Sub-Saharan African countries which account for more than 53% of the global under-five mortality. In Ethiopia, though U5M shows a substantial decrease from 1990 to 2019, it is still unacceptably high and unevenly distributed. Therefore, this study aimed to assess geographic variation and factors associated with under five mortality in Ethiopia.

Method

We sourced data from the most recent nationally representative 2019 Ethiopian mini Demographic and Health Survey ~~data~~ for this study. A sample size of 5,695 ~~live~~ births were considered for this study. Descriptive and analytical ~~Analysis was~~ conducted by using STATA version 16. Both multilevel and spatial analyses were employed to ascertain the factors associated with under-five mortality in Ethiopia.

Result

The under-five mortality is 5.9% with its 95% CI 5.4% to 6.6%. Based on the multivariable multilevel logistic regression model ~~result;~~ family size (AOR=0.09, 95%CI:(0.84, 0.99)), number of under-five children in the family (AOR=0.17, 95%CI: 0.14, 0.21), multiple birth (AOR=14.4, 95%CI: (8.5, 24.3)), Children who breastfed for less than 6 months (AOR=5.04, 95%CI: (3.81, 6.67)), People whose main roof is palm (AOR=0.57, 95%CI: 0.34, 0.96), Under-five children who are at birth order sixth or more (AOR=2.46, 95%CI: (1.49, 4.06)), institutional delivery (AOR=0.57, 95%CI: (0.41, 0.81)), being resident of Somali and Afar region (AOR=3.46, 95%CI: (1.58, 7.55)) and (AOR=2.54, 95%CI: (1.10, 5.85)) respectively were factors associated with under-five mortality. A spatial analysis result revealed that hot spot areas of under-five mortality were located in Somali ~~region~~ and Dire Dawa.

Conclusion

Under-five mortality in Ethiopia is ~~59 per 1000 live births~~ which is high and unacceptable as compared to the 2030 ~~agenda for~~ sustainable development aimed at 25 per 1000 live births. ~~Breast feed~~ less than 6 month, twin birth, institutional delivery and high risk areas of under-five mortality (Somali and dire Dawa) ~~were~~ modifiable risk factors. Therefore maternal education on the advantage of breast feeding and institutional delivery is highly recommended. Women who deliver twins should be given special attention. An effective strategy should be designed for ~~intervention for~~ hot spot areas of under-five mortality that ~~is~~ Somali and Dire Dawa.

Order of Authors: Zemenu Tadesse Tessema, MSC Biostatistics

	Tsion Mulat Tebeje
	Lewi Goytom Gebrehewet
**Opposed Reviewers:**	
**Response to Reviewers:**	PLOS ONE<br>Point by point response for editors/reviewers' comments<br>The manuscript title "Geographic variation and factors associated with under-five mortality in 2 Ethiopia. A spatial and multilevel analysis of Ethiopian Mini Demographic and 3 Health Surveys 2019"<br>Manuscript number: PONE-D-21-20589<br>Dear editor/reviewer,<br>I would like to thank you for these constructive, building, and improvable comments on this manuscript that would improve the substance and content of the manuscript. We considered each comment and clarification question of reviewers on the manuscript thoroughly. My point-by-point responses for each comment and question are described in detail on the following pages. Further, the details of changes were shown by track changes in the supplementary document attached.<br><br>Reviewer comment and authors response<br>Reviewer #1:<br>General comments:<br>Reviewer's comment: - Overall authors have explained regional variation in under five mortality and the key predictors of U5M in Ethiopia. I have few comments to improve this paper further.<br>Reviewer's comment:<br>1. Authors have used random intercept model for binary multilevel logistic regression. Authors can also do some more testing of their model by including random slopes for their significant predictors in this model to check if the effect of their key predictors remains the same for each cluster or is it different. If they don't find any major difference by including the random slopes, then they can provide the current results as it is.<br>Authors response: thanks for the detailed comment we have tested using the random slop in checking its effect and didn't find noticeable difference from the random intercept model.<br><br>Reviewer's comment:<br>2. Authors have used different household condition indicators such as toilet clean fuel main roof etc. than wealth index which captures the economic condition of the household better in the analysis. Is there any specific reason to adapt this strategy?<br>Authors response: Thank you very much for your comment. We have considered using the wealth index as an economic condition of the household in our analysis, and the wealth index variable failed to enter the multivariant multilevel mixed model since the significant level was high during the univariable multilevel mixed model.<br>Reviewer's comment:<br>3. Authors should also provide the residuals plots for their final multilevel model in the appendix.<br>Authors response: thanks again for the detailed comment, we have provided the residual plots for the final model in figure 6 and on page 10, line 248-249, kindly refer to the revised manuscript.<br>Reviewer's comment:<br>4. Authors have shown that there is a significant large cluster of population where U5MR is significantly higher than other areas in Figure 4. Authors can include a table with basic socio demographic profile of this high cluster region. This can be helpful to understand which population mostly suffers with high U5M in that region so that specific interventions and programmes can be targeted for those subgroups of population.<br>Author's response: Thank you very much for you very constructive comment. In the descriptive statistics section example in table one already reported. We believe that no need to include the specific socio-demographic characteristics of the hot sport areas. If there any help for us we will do it in next round revision.<br>Reviewer's comment:<br>5. Authors should include a short paragraph in the discussion on the implications of their findings in context of the SDG goals.

Powered by Editorial Manager® and ProduXion Manager® from Aries Systems Corporation

Authors response: Thank you very much for your comment. We have accepted the comment and kindly refer to the revised manuscript specified on page 10, line 258 up to 260.

Reviewer's comment:
6. Please see the flow of your texts in the discussion once more and edit the sentences wherever required for more clarity.
Authors response: Thank you very much for your comment. We have accepted the comment and kindly refer to the description part of the revised manuscript.
Reviewer #2:
General comments:
Reviewer's comment: - This publication is of high importance because it attempts to present sophisticated analyses of a legitimate population-based survey data to highlight key risk factors for under five mortalities in Ethiopia. However, there are two problems with the manuscript: the first is that it is poorly written in English, which makes understanding of all processed hard; secondly, there are some explanations about the statistics used that (partly due to the poor English) are difficult to understand. Double check both the English and the statistical methods used (binary, bivariable? multi-level logistic regression?).
Reviewer response: thank you for your comment. We accept and kindly find the revised manuscript. When we say binary, we want to explain that our outcome variable has only the possibility of failure or success, and also with regards to bivariable, we wanted to illustrate that we first run a multilevel logistic regression model consisting of the outcome variable and one predictor variable for each predictor variables then choosing those predictors that have passed our significant level (less than the P-value of 0.2) to the multivariable multilevel logistic regression model. We used multilevel logistic regression due to the hierarchical nature of the data.
Reviewer #3:
General comments:
Reviewer's comment: - This is an engaging, insightful paper and mostly methodologically sound paper.
There is one important thing to resolve. The authors need to explain why the geospatial analysis was pertinent. Sure, it produced great maps, but the inferences that made it to the Discussion and Conclusion sections could have been made with simple aggregation instead of the employed GIS methods. If this is not the case, the authors need to explain why their approach was used and discuss that in the Discussion section.
Authors response: thank you for your detailed comment, we accepted and the main reason we employed GIS is for cost minimization and to help the policymakers to focus on the arias noted by GIS and saTscan, we have also edited in the discussion section specifically on page 11, line 300-302 in the revised manuscript.
Reviewer's comment:
The paper should undergo professional proofreading as some formulations were strange.
There were some minor mistakes and suggestions, which were marked and commented on in the attached PDF.
Authors response: thank you for your comments. We accept and kindly find the revised manuscript.
Reviewer #4
Reviewer's comment: - I have reviewed the manuscript and it brings a perspective of spatial and multilevel analysis to understanding the variations in under-five mortality in Ethiopia. The multilevel analysis introduces community level variables not examined before enabling the study to consider factors beyond individual ones.
The study uses more recent data. The method of data analysis, that is, multivariate multilevel logistic regression is fine but its limitation is that it does not handle the aspect of censored data well.
Authors response: thanks for the comment, our data is cross-sectional hierarchical data with a binary outcome and for this type of data multilevel logistic regression is the best method of data analysis.
Reviewer's comment:
In performing the analysis, it is not clear in the manuscript if the data were weighted or not.
Authors response: thanks for the comment we have accepted and revised it kindly find

Powered by Editorial Manager® and ProduXion Manager® from Aries Systems Corporation

on page 6 line 166 up to line 167 in the revised manuscript.
Reviewer's comment:
The data source section (page 4, lines 91-108), could have been summarized further and high light the information collected by the 2019 EMDHS relevant to study.
Authors response: thanks for the comment we accepted and kindly refer to the revised manuscript specifically (page 4, lines 91- 97).
Reviewer's comment:
On study variables (page 5, lines 112-122), the presentation of the variables could have been efficiently done in a table with definitions and categorization. More especially for community level variables.
Authors response: thank you for your comment, we have considered using the table to illustrate the study variables and since the variables are presented in table 1 up to 3 additional tables, we feared that an additional table might be a bit redundant.
Reviewer's comment:
There are too many variables considered at individual level. Is it possible to reduce them to relevant ones only based on reviewed literature?
Authors response: thanks for your detailed comment, all of our variables are from literature reviews and we have reduced many not significant variables during the bivariable multilevel logistic regression.
Reviewer's comment:
For spatial analysis (page 5, lines 124-125), state what was actually done. Highlight the benefits and limitations of using multilevel analysis.
Authors response: thank you for the detailed comment the spatial analysis that was employed are reported in detail on page 5-6, line 127-145.
Reviewer's comment:
In the results section (pages 8-9), is possible to use 1 decimal place for the percentages in the text. Also, check the table titles and ensure they are named appropriately.
Authors response: thanks for the detailed comment, we have considered using one decimal place and since we wanted to be precise as possible that is why we used two decimal places.
Reviewer's comment:
For multilevel analysis results, the intra-cluster correlation (ICC) is not shown in the table.
Authors response: thanks for the detailed comment, we accept and kindly find the revised table on page 24 in the revised manuscript.
Reviewer's comment:
A theoretical framework work should have been considered by the study.
Authors response: thank you for the detailed comment, we have provided the theoretical framework kindly find the revised manuscript specifically in figure 1.
Reviewer's comment:
In the discussion section of the manuscript, emphasis should have been on discussing community level factors and the geographical variation in under-five mortality observed.
Authors' response: thanks for the detailed comment, we only discussed the community factors that are significantly associated with under-five mortality, and most of the community-level factors are not significantly associated with under-five mortality.
Reviewer's comment:
The manuscript should have discussed if the under-five mortality rates in the 2019 EMDHS were reproduced for benchmarking prior to deriving its own.
Authors response: thanks for your comment we have discussed benchmarking prior to EDHS, kindly find it on page 10 between the lines 258-261.
Reviewer's comment:
The conclusion and recommendations of the manuscript should have been based on the multilevel analysis (community level variables) results in addition to the spatial variation findings on under-five mortality.
Authors' response: thanks for the detailed comment, most of the community-level variables were not significantly associated with under-five mortality that is why we didn’t give focused on the community-level variables in the conclusion.
Reviewer #5
Reviewer's comment: This paper is well motivated and carries out a series of multivariate and spatial analysis on Ethiopian data on under 5 child mortality. The survey data appears to be of excellent quality from a rigorous sampling design. The spatial analysis is a strong point and allows subregional rates to be detected that are

Powered by Editorial Manager® and ProduXion Manager® from Aries Systems Corporation

higher or lower than the background. Spatial scan statistics provide a promising approach in this case and are good complements to the other approaches.
The authors should properly cite the SaTScan software, along with the author M.Kulldforf (see the user manual). I would encourage citing Stata properly as well (note spelling and case).
Authors response: thank you for your detailed comment, we accepted kindly refer to the revised manuscript specifically page 6, lines 139 and 143.
Reviewer's comment:
In my experience, SaTScan runs need quite a bit of tinkering of the parameter file to get good hot spot representation (i.e., like that in the spatial EB approach) . I think the cluster detection can be improved by selecting a smaller window size or radius in order to detect smaller secondary clusters other than the primary two presented here.
Authors response: thanks for the detailed comment, we agree that the cluster can be improved by selecting a smaller window size to detect smaller secondary clusters but we aim to identify the primary ones that is why we used the default maximum spatial cluster size of less than 50% of the population, which allows both small and large clusters to be detected.
Reviewer's comment:
Otherwise, this is an informative paper on an important topic. Table 4 could use better formatting. Please consider using coefficient plots. Additional proofing of the manuscript is also recommended to improve clarity.
Authors response: thank you for your detailed comment, we have improved the clarity kindly refer to the revised manuscript. It known that it possible to report using coefficient plot but we believe table four format appropriate in this regard.
Reviewer #6
Reviewer's comment:
This is a thoughtful and interesting analysis of under-five mortality in Ethiopia in the years preceding the current crisis and civil war. The analysis focusses on the major individual and area level variables that have been identified in the literature and the results are largely in line with previous findings. While my overall evaluation is positive there are a number of issues that need to be addressed before publication:
1. The style of writing is extremely telegraphic, to the point that at times the meaning is not strictly clear. For instance, your community level variables refer to place of delivery; toilet use; wealth and media exposure. I presume these are based on majority characteristics from your individual level analysis, but this should be made clearer. In particular, I see no reference to media exposure at the individual level, so what does this variable refer to?
Authors response: thank you for your detailed comment, we accepted and kindly refer to the revised manuscript specifically page 5, line 115-123.
Reviewer's comment:
2. A major contribution of this paper is the difference between the different areas of Ethiopia and the relation to community characteristics. It would be important, for non-Ethiopian readers, to provide a bit more background on the different regions, socially and historically. A comparison based on the community characteristics you describe would be particularly useful. You should also provide references for the spatial analysis you present (Moran's I and the spatial interpolation).
Authors response: thank you for your comment, we didn't find any significant community characteristics that's why we didn't compare based on the community characteristics. Also, we accepted and kindly find the revised manuscript for the edited reference on pages 5, line 130, and 135 respectively.

Reviewer's comment:
3. Your choice of model (multilevel binomial) is appropriate but the presentation is a bit overloaded. Many of the variables appear to be non-significant in the multivariate model, even if they were significant at the bivariate level. This may be due to collinearity between the variables. Removing the non-significant variables would bring out better the major covariates related to U5M.
Authors response: thanks for the detailed comment, we have checked for collinearity and there was no noticeable collinearity between variables.
Reviewer's comment:
It is also important to indicate that the coefficients are relative risks (eb). There is no need for both log-likelihood and deviance values (as you need, D = -2LL) but you should indicate the degrees of freedom and the variance of the random effects (clusters) as well as the number of individual cases (level 1) and clusters (level 2).

Powered by Editorial Manager® and ProduXion Manager® from Aries Systems Corporation

Authors response: thanks for the detailed comment, we accept we have edited out the deviance from the table kindly refer to the revised table 4 in the revised manuscript. As for the number of individual cases and clusters we thought it would be repetition since it has been mentioned in the data source page 4, lines 101 and 105, and also on the result section on page 8, line 194.
Reviewer's comment:
4. Your discussion focusses on the major factors reducing under 5 mortalities, but you do not discuss differences between the models. For instance, use of solid fuel increases U5M in model 2 and reduces it in model 4; Amhara has higher mortality in model 3, lower mortality in model 4. In both cases, the coefficients are non-significant (within the CI) but the reverse should still be mentioned (also see previous comment on removing non-significant comparisons).
Authors response: thanks for the detailed comment. Indeed it is advantageous to discuss the differences between the models, we feer that the paper might loos its pertinence.

Reviewer's comment:
5. Some of the comments in your discussion appear to be inconsistent. For instance, larger families have lower mortality, but so do higher birth orders. You mention mother’s age at first birth but not at current birth, which may be important in explaining this paradox. You rightly identify risk factors subject to intervention, such as short breast feeding or home delivery, but I fail to see how twin births fall into this category, except in the sense that greater attention should be given to mothers with multiple births.
Authors response: thanks for the comment, Since also multiple births are prone to develop short to long-term complications kindly refer to the citation (32). We accept and we edited in multiple births and compared it with singleton birth kindly refer to the revised manuscript specifically on page 12, line 313.
Reviewer's comment:
In sum, this is a valuable contribution which I hope to see published, and my comments are intended to strengthen what is, at heart, a sound analysis based on a rich and valuable dataset.
Reviewer #7
Reviewer's comment:
The paper is sound and generally well-written. The topic is very relevant and the analyses performed compatible and consistent with the research question. I have some minor remarks that I consider should be addressed before approving for publication:
Overall, please explain a bit further the difference between the mini-DHS survey and the main DHS survey. Why do you think this new data is helpful to track the trends in U5M? The authors extensively explain the sampling design, but do not explain the difference between the mini and the main DHS survey, which would be helpful in interpreting the results. Is the mini-DHS survey comparable to the main DHS survey? Can we use this to compare them in time?
Authors response: thanks for the detailed comment. Indeed it is advantageous to discuss the differences between the mini-DHS and main DHS, we feer that the paper might loos its pertinence.

Reviewer's comment:
In addition, it would be important to know whether any data quality assessment of deaths was performed before the regression analysis. Would any bias be added to the study due to misreporting of deaths in the survey or issues related to coverage areas? Did the authors correct the registry of deaths in any way or were deaths under 5 taken as it is from the survey? How do the authors feel about this data and do they trust the information?
Authors response: Thank you very much for your ideal comment. Frankly speaking, we authors had no any room to check death registry. Since is secondary data and there is no strong death registry in the country it is difficult to check death by any other means. Before the data is collected training was given for data collectors this may be one way data quality assurance. The second it large survey and conducted carefully with expertise and representative of the country under-five mortality. The information is trustful.
Reviewer's comment:

Powered by Editorial Manager® and ProduXion Manager® from Aries Systems Corporation

	In addition, some minor remarks/suggestions in the writing part that felt confusing:<br>lines 82- 84: the sentence is confusing. I would add here a period. and Say: "Though U5M has been declining, it is still high and is taking the lives of many children. Despite this fact, no research on U5M has been done with recent data available from the Ethiopian mini ... "<br>Authors response: thank you for your detailed comment, we accepted and kindly find the edited statements on pages 3 and 4, lines 81 – 84.<br>Reviewer's comment:<br>lines 85-87: similarly, the sentence is confusing. I would suggest: "...to show the current burden of U5M that is crucial for better planning different policies and interventions for U5M prevention. This allows for an efficient allocation of scarce resources according to spatial..."<br>Authors response: thank you for your detailed comment, we accepted and kindly find the edited statements on page 4 lines 85 – 88.<br>Reviewer's comment:<br>lines 188: suggestion to make the sentence clearer: "Sociodemographic background" and not "background socio-demographic"<br>Authors response: thank you for your detailed comment, we accepted and kindly find the edited statements on page 8, line 193.<br>Reviewer's comment:<br>line 195: correct to: "are of poor economic status"<br>Authors response: thank you for your detailed comment, we accepted and kindly find the edited statements on page 8, line 199.<br>Reviewer #8<br>Reviewer's comment:<br>This paper aims to examine geographic variation and factors attributable to Under-Five Mortality (UFM) in Ethiopia. Recognizing as an important marker for health equity and access, UFM is considered the best proxy measure or indicator for socioeconomic development. In addition, child mortality rate is also a useful marker of overall development and a Millennium Development Goal (MDG) indicator and its importance has been further emphasized in an ambitious target under the Sustainable Development Goals. Generally, literature search reveals numerous or countless studies on factors determining UFM conducted in the Sub-Saharan African countries, however, spatial distributions and geographic variations are less investigated compared to the UFM factors.<br>The data sources used, statistical tests and analysis performed systematically support, but NOT to the full extent, in answering the research questions and its intended aims or objectives. Results and conclusion made in relation to research aims and objectives or intentions are supported by data and analysis, in general. However, there needs further refinement and improvement for author(s) to take into the consideration to make this paper more added-value to the knowledge and literature.<br>I foresee two major drawbacks of this study, which are described below:<br>Reviewer's comment:<br>1. The Primary Investigator should not limit the independent variables to individual and community level predictors. It is suggested to take into the consideration of inclusion of health interventions, such as malaria, sanitation and hygiene (WASH), reproductive health (RH), vaccinations, micronutrient supplementation and treatments. Most studies have revealed that there are significant association between these health interventions and reduction of UFM. For example, BCG, OPV, Measles, TT, etc. vaccinations have significantly contributed to drastic reduction in deaths of children in most developing countries. Furthermore, measure DHS captures or collects information on health interventions; and it's worthwhile to examine its association with UFM in Ethiopia.<br>Authors response: thank you for the detailed comments, we have considered using the above-listed variables, and some we used like WASH variables (time to water), and contraceptive utilization, but still some variables like (water source and toilet type) were not able to pass the bivariable multilevel logistic regression and some variables like (BCG, OPV, measles, and TT) were no available in the EMDHS data.<br>Reviewer's comment:<br>2. Since the other known predictors of child morality are generally attributable to food security and accessibility in conflict areas thus, it is recommended to discuss how such conflicts or disruptions would influence or alter the geographic variations of UFM in Ethiopia? Other risk factors to take into considerations are: indoor air pollution (determined by type of cooking fuel used in household), nutrition, access to basic health services (ANC, PNC, FP, RH, etc.), poverty status by regions, fertility rates,

Powered by Editorial Manager® and ProduXion Manager® from Aries Systems Corporation

	among others.<br>Authors response: thank you for the detailed comments, the EMDHS data doesn't possess the data that is attributable to food security and whether the EA was a conflict area at the time of collecting the data. additionally, we have considered indoor air pollution and added the variable of cooking fuel to the final models but the results were insignificant and we didn't want to discuss its results. We have also considered basic health services and most were insignificant during the bivariable multilevel logistic regression model and were not added to the multivariable multilevel logistic regression model.
**Additional Information:**	
**Question**	**Response**
**Financial Disclosure**<br><br>Enter a financial disclosure statement that describes the sources of funding for the work included in this submission. Review the submission guidelines for detailed requirements. View published research articles from *PLOS ONE* for specific examples.<br><br>This statement is required for submission and **will appear in the published article** if the submission is accepted. Please make sure it is accurate.<br><br>**Unfunded studies**<br>Enter: *The author(s) received no specific funding for this work.*<br><br>**Funded studies**<br>Enter a statement with the following details:<br>• Initials of the authors who received each award<br>• Grant numbers awarded to each author<br>• The full name of each funder<br>• URL of each funder website<br>• Did the sponsors or funders play any role in the study design, data collection and analysis, decision to publish, or preparation of the manuscript?<br>• **NO** - Include this sentence at the end of your statement: *The funders had no role in study design, data collection and analysis, decision to publish, or preparation of the manuscript.*<br>• **YES** - Specify the role(s) played.<br><br>* typeset	The authors received no specific funding for this work.
**Competing Interests**<br><br>Use the instructions below to enter a	The authors have declared that no competing interests exist.

competing interest statement for this submission. On behalf of all authors, disclose any competing interests that could be perceived to bias this work—acknowledging all financial support and any other relevant financial or non-financial competing interests.<br><br>This statement is required for submission and **will appear in the published article** if the submission is accepted. Please make sure it is accurate and that any funding sources listed in your Funding Information later in the submission form are also declared in your Financial Disclosure statement.<br><br>View published research articles from *PLOS ONE* for specific examples.<br><br>**NO authors have competing interests**<br><br>Enter: *The authors have declared that no competing interests exist.*<br><br>**Authors with competing interests**<br><br>Enter competing interest details beginning with this statement:<br><br>*I have read the journal's policy and the authors of this manuscript have the following competing interests: [insert competing interests here]*<br><br>* typeset	
**Ethics Statement**<br><br>Enter an ethics statement for this submission. This statement is required if the study involved:<br><br>• Human participants<br>• Human specimens or tissue<br>• Vertebrate animals or cephalopods<br>• Vertebrate embryos or tissues<br>• Field research	Ethical clearance was obtained from measure DHS through filling requesting a form for accessing data. The data used in this study are publicly available, aggregated secondary data that hasn’t any personal identifying information that can be linked to study participants. The confidentiality of data was maintained anonymously.

Write "N/A" if the submission does not require an ethics statement.

General guidance is provided below. Consult the submission guidelines for detailed instructions. **Make sure that all information entered here is included in the Methods section of the manuscript.**

Format for specific study types

Human Subject Research (involving human participants and/or tissue)

- Give the name of the institutional review board or ethics committee that approved the study
- Include the approval number and/or a statement indicating approval of this research
- Indicate the form of consent obtained (written/oral) or the reason that consent was not obtained (e.g. the data were analyzed anonymously)

Animal Research (involving vertebrate animals, embryos or tissues)

- Provide the name of the Institutional Animal Care and Use Committee (IACUC) or other relevant ethics board that reviewed the study protocol, and indicate whether they approved this research or granted a formal waiver of ethical approval
- Include an approval number if one was obtained
- If the study involved *non-human primates*, add *additional details* about animal welfare and steps taken to ameliorate suffering
- If anesthesia, euthanasia, or any kind of animal sacrifice is part of the study, include briefly which substances and/or methods were applied

Field Research

Include the following details if this study involves the collection of plant, animal, or other materials from a natural setting:

- Field permit number
- Name of the institution or relevant body that granted permission

Powered by Editorial Manager® and ProduXion Manager® from Aries Systems Corporation

**Data Availability**<br><br>Authors are required to make all data underlying the findings described fully available, without restriction, and from the time of publication. PLOS allows rare exceptions to address legal and ethical concerns. See the PLOS Data Policy and FAQ for detailed information.<br><br>A Data Availability Statement describing where the data can be found is required at submission. Your answers to this question constitute the Data Availability Statement and **will be published in the article**, if accepted.<br><br>**Important:** Stating 'data available on request from the author' is not sufficient. If your data are only available upon request, select 'No' for the first question and explain your exceptional situation in the text box.<br><br>Do the authors confirm that all data underlying the findings described in their manuscript are fully available without restriction?	Yes - all data are fully available without restriction
**Describe where the data may be found in full sentences. If you are copying our sample text, replace any instances of XXX with the appropriate details.**<br><br>• If the data are **held or will be held in a public repository**, include URLs, accession numbers or DOIs. If this information will only be available after acceptance, indicate this by ticking the box below. For example: *All XXX files are available from the XXX database (accession number(s) XXX, XXX.*).<br>• If the data are all contained **within the manuscript and/or Supporting Information files**, enter the following: *All relevant data are within the manuscript and its Supporting Information files.*<br>• If neither of these applies but you are able to provide **details of access elsewhere**, with or without limitations, please do so. For example:	Third party data was obtained for this study from The DHS Program. Data may be requested from The DHS Program after creating an account and submitting a concept note. More access information can be found here: https://dhsprogram.com/data/Access-Instructions.cfm<br><br>The authors confirm that interested researchers would be able to access these data in the same manner as the authors. The authors also confirm that they had no special access privileges that others would not have.

Powered by Editorial Manager® and ProduXion Manager® from Aries Systems Corporation

Data cannot be shared publicly because of [XXX]. Data are available from the XXX Institutional Data Access / Ethics Committee (contact via XXX) for researchers who meet the criteria for access to confidential data.<br><br>*The data underlying the results presented in the study are available from (include the name of the third party and contact information or URL).*<br>• This text is appropriate if the data are owned by a third party and authors do not have permission to share the data.<br><br>* typeset	
Additional data availability information:	

March 23/ 2022

Gondar, Ethiopia

Subject: **Cover Letter**

Dear editors,

We intended to submit our research manuscript entitled **“Geographic variation and factors associated with under-five mortality in Ethiopia. A spatial and multilevel analysis of Ethiopian Mini Demographic and Health Surveys 2019. ”** in your journal **PLOS ONE** as a research article. The reason for considering is for its open access, the fast publication process, rigorous peer-review, and high visibility. The manuscript was not submitted elsewhere. In this regard, we request you to kindly consider our manuscript to publish in your esteemed journal.

Regarding

Editors Comment : Are these third party data (i.e., data not owned or collected by the author(s))?

Authors response:- Yes , the data is obtained from third pary that is fronm DHS program after a short project concept submitted. Therefore other authors can access this dataset from DHS program after create an account and prepare a concept note that they are interested. The program websiate is www.dhsprogram.com

Editors Comment:-Figures

Authors response:-The figures in the manuscript was obtained from WWW.OpenAfrica Shapfile free and obtained from this website

Best Regards:

Zemenu Tadesse Tessema,

March 23/ 2022

Gondar, Ethiopia

Subject: **Cover Letter**

Dear editors,

We intended to submit our research manuscript entitled **"Geographic variation and factors associated with under-five mortality in Ethiopia. A spatial and multilevel analysis of Ethiopian Mini Demographic and Health Surveys 2019. "** in your journal **PLOS ONE** as a research article. The reason for considering is for its open access, the fast publication process, rigorous peer-review, and high visibility. The manuscript was not submitted elsewhere. In this regard, we request you to kindly consider our manuscript to publish in your esteemed journal.

Regarding

Editors Comment : Are these third party data (i.e., data not owned or collected by the author(s))?

Authors response:- Yes , the data is obtained from third pary that is fronm DHS program after a short project concept submitted. Therefore other authors can access this dataset from DHS program after create an account and prepare a concept note that they are interested. The program websiate is www.dhsprogram.com

Editors Comment:-Figures

Authors response:-The figures in the manuscript was obtained from WWW.OpenAfrica Shapfile free and obtained from this website

Best Regards:

Zemenu Tadesse Tessema,

Manuscript Click here to access/download;Manuscript;revised final manuscript.docx

Geographic variation and factors associated with under-five mortality in Ethiopia. A spatial and multilevel analysis of Ethiopian Mini Demographic and Health Surveys 2019.

Zemenu Tadesse Tessema [1*], Tsion Mulat Tebeje[2], Lewi Goytom Gebrehewet[1]

[1] Department of Epidemiology and Biostatistics, Institute of Public Health, College of Medicine and Health Sciences, University of Gondar, Gondar, Ethiopia

[2] Epidemiology and Biostatistics Unit, School of public health, Dilla University, Dilla, Ethiopia

E-mail : ZTT zemenut1979@gmail.com

TMT yemarina12@gmail.com

LGG lewigt369@gmail.com

***= Corresponding author**

Abstract

Background: The distribution of under-five mortality (U5M) worldwide is uneven and the burden is higher in Sub-Saharan African countries which account for more than 53% of the global under-five mortality. In Ethiopia, though U5M shows a substantial decrease from 1990 to 2019, it is still excessively high and unevenly distributed. Therefore, this study aimed to assess geographic variation and factors associated with under-five mortality in Ethiopia.

Method: We sourced data from the most recent nationally representative 2019 Ethiopian Mini-Demographic and Health Survey data for this study. A sample size of 5,695 live births was considered for this study. Descriptive and analytical Analysis was conducted by using STATA version 16. Both multilevel and spatial analyses were employed to ascertain the factors associated with under-five mortality in Ethiopia.

Result: The under-five mortality is 5.9% with its 95% CI 5.4% to 6.6%. Based on the multivariable multilevel logistic regression model result; family size (AOR=0.09, 95%CI :(0.84, 0.99)), number of under-five children in the family (AOR=0.17, 95%CI: 0.14, 0.21), multiple birth (AOR=14.4, 95%CI: (8.5, 24.3)), Children who breastfed for less than 6 months (AOR=5.04, 95%CI: (3.81, 6.67)), People whose main roof is palm (AOR=0.57, 95%CI: 0.34, 0.96), Under-five children who are at birth order sixth or more (AOR=2.46, 95%CI: (1.49, 4.06)), institutional delivery (AOR=0.57, 95%CI: (0.41, 0.81)), being resident of Somali and Afar region (AOR=3.46, 95%CI: (1.58, 7.55)) and (AOR=2.54, 95%CI: (1.10, 5.85)) respectively were factors associated with under-five mortality. A spatial analysis result revealed that hot spot areas of under-five mortality were located in the Somali region and Dire Dawa.

Conclusion: Under-five mortality in Ethiopia is 59 per 1000 live births which are high and unacceptable as compared to the 2030 agenda for sustainable development aimed at 25 per 1000 live births. Breastfeeding less than 6-month, twin birth, institutional delivery and high-risk areas of under-five mortality (Somali and dire Dawa) were modifiable risk factors. Therefore, maternal education on the advantage of breastfeeding and institutional delivery is highly recommended.

Women who deliver twins should be given special attention. An effective strategy should be designed for intervention for hot spot areas of under-five mortality that is Somali and Dire Dawa.

Keywords: Under-five mortality, factors, spatial distribution, multilevel, Ethiopia

Background

Under-five mortality is defined as deaths reported at ages 0 to 59 months and it contains neonatal, post-neonatal, and child deaths (1). Under-five mortality rate (U5MR) is the probability of a child dying before celebrating the fifth birthday(2). It is a significant indicator of the socioeconomic, health, environmental conditions, and national development of health equity and access(3).

Globally, in 1990, under-five deaths were 12,494,000 (93 deaths per 1000 live births). By showing an average annual decline of 3.1%, in 2019 it was significantly reduced to 5,189,000 deaths (38 deaths per 1000 live births)~~ with~~ a 58% reduction(4). The 2030 ~~agenda for~~ sustainable development is ~~aimed at~~ ending the preventable deaths of newborns and children under 5 years of age, with all countries aiming to reduce under-5 mortality to make it at least ~~less than~~ 25 per 1000 live births(5).

The distribution of U5MR worldwide is uneven~~ and has wider disparity and~~ the global burden of U5MR is concentrated ~~at~~ two ~~sustainable development goals (SDG)~~ regions, Sub-Saharan Africa and Central and Southern Asia~~ with~~ more than 80% of U5MR ~~accounted for~~(6). The burden is higher in Sub-Saharan African countries with 74 deaths per 1000 live births, which accounts for more than 53% of the global under-five mortality(7).

In Ethiopia, U5MR ~~shows~~ a substantial decrease from 200 per 1000 live births in 2019 to 55 per 1000 live births in 2019 but still couldn't ~~achieve~~ the SDG ~~aim~~(4), additionally different studies conducted in Ethiopia revealed several ~~S~~ocioeconomic, demographic, and geographic or spatial variation ~~is considerable, and~~ Afar, Somali, and Benishangul Gumuz are the three high-risk regions of Ethiopia for U5MR(8).

Different studies conducted on U5M previously showed that source of water(9), multiple births (7, 8), region (10, 11), age and sex of household head (11), maternal education status (12, 13), place of delivery(10, 11, 14), maternal age(15-17), sex of the child(9, 14, 17), residence (18, 19), family size (10, 16), number of children under five(19, 20), birth order (10, 16), and breastfeeding status (19, 21) have a significant association with under-five mortality.

Though U5M has been declining it is still high and is taking the lives of many children, and no research on U5M has been made ~~on the recent survey data~~, Ethiopian mini–Demographic Health Survey (EMDHS) 2019. Therefore, to make different policies and interventions ~~of~~ U5M prevention with the efficient allocation of scarce resources ~~according to spatial variability~~ based on our findings and to achieve the SDG by understanding the determinants, identifying areas and factors with the highest burden of U5M in Ethiopia is helpful to show the current burden of U5M, ~~hence~~ this study is aimed at assessing the geographic variation and factors associated with ~~U~~nder-five mortality in Ethiopia.

Method

Data Source

Our source was the 2019 EMDHS is the second mini demographic health survey (DHS) conducted in Ethiopia, ~~which is~~ a land-locked country located in the Horn of Africa that lies between the 30 N and 150N Latitude or 330 E and 480 E Longitude(22). Data collection was conducted from March 21, 2019, to June 28, 2019, from the nine regions (Tigray, Afar, Amhara, Oromia, Somali, Benishangul Gumuz, SNNPR, Harari, and Gambella) and two administrative cities (Addis Ababa and Dire Dawa). The study design was a population-based cross-sectional study. Kids Record (KR) format was the data of interest to conduct the study. A frame of all census Enumeration areas (EAs) was used as a sampling frame for the 2019 EMDHS. 149,093 EAs were created which covers an average of 131 Households (HHS). A two-stage stratified cluster sampling technique was employed and each region was stratified into urban and rural areas, yielding 21 sampling strata. ~~Enumeration areas (~~EAs~~)~~ were selected independently in each stratum. In the first stage, 305 clusters (93 urban and 212 rural) were selected with probability proportional to EAs size and with independent selection in each sampling stratum. In the second stage, a fixed number of 30 households per cluster were selected. Finally, women aged 15–49 in 9150 (2790 urban and 6360 rural) households from 305 clusters were selected. The whole procedure of sampling is found in the full 2019 EMDHS report(22).

Study variables

The response variable was under-five mortality status, which was categorized as (child alive: Yes= 0 and No = 1). The age was recorded in months. Independent variables were classified as individual level and community level predictors (Place of residence, Region, Community place of delivery, Community wealth, Community media exposure, and Community toilet facility), some of this community-level predictors are driven from individual-level predictors. The individual-level predictors were further categorized as socio-demographic and economic factors (Educational level of the mother, Sex of household head, age of household head, Number of household members, Number of a child under the age of five, Marital status of the mother, Source of water, Time to get water, Type of toilet facility, Household electricity, Types of cooking fuel, Main floor material, Main wall material, and Main roof material) and Maternal and child factors (Maternal age at first birth, Sex of the child, Utilization of contraception, Order of birth, Mode of delivery, duration of breastfeeding and Multiple births) (**Figure 1)**.

Spatial analysis

The data for spatial analysis was cleaned and merged by using STATA version 16 and excel soft wares. ArcGIS version 10.8 and saTScan version 9.7 were used for the spatial analysis.

Spatial autocorrelation

Spatial autocorrelation (Global Moran's I) was conducted to see if under-five mortality was measured as dispersed (Moran's I value closer to -1), clustered (Moran's I value closer to 1), or randomly distributed (Moran's I value of 0) in Ethiopia (23).

Spatial interpolation

Though all enumeration areas have known under-five mortality, areas that are not selected were predicted. Spatial interpolation was applied by using the geostatistical ordinary Kriging spatial interpolation technique to predict under-five mortality from a limited number of sample data points (EAs) to un-sampled areas (24).

Spatial scan statistics

SaTscan was used based on the Bernoulli test for cases (child is not alive) and controls (child is alive) to conduct the scan analysis. The upper limit used was the default maximum spatial cluster size of less than 50% of the population, allowing both small and large clusters to be detected, and clusters that contained more than the maximum limit with the circular shape of the window were avoided(25). Most likely clusters were identified using p-values and likelihood ratio tests, which is the ratio of the likelihood of the alternative hypothesis (higher activity level inside the window) over the likelihood of the null hypothesis (same activity level inside and outside).

Data management and analysis

We used STATA version 16 and R statistical software version 4.0.5 to analyze the data. Data (K[illegible] was cleaned and 31 variables were kept that were needed for the analysis. Residents who are not De jure (included in the study if they ~~lasted~~ the night before the study) were excluded ~~which~~ affect under-five mortality, as they ~~couldn't~~ respond to most of the socio-demographic and economic characteristics though they ~~can~~ answer the maternal and child characteristics~~, which has~~ ~~changed~~ our sample size from 5753 to 5695.

The outcome variable was re-coded to (child alive: Yes= 1 and No = 0). ~~The variables were further recording, renaming, labeling, and generating new variables from the original variables was done. These additional~~ four [illegible]mmunity variables were generated by taking the individual variable, calculating their proportion, and dichotomizing them based on their mean or median according to their distribution ~~and~~ merging variables.

In the end, we had 29 predictor variables out of which 6 were community-level predictor variables and 23 individual-level predictor variables.

As we have a binary outcome variable (child alive = yes/no), to analyze factors associated with under-five mortality, binary logistic regression is appropriate. But based on EMDHS; respondents ~~with~~ the same cluster show similar findings or functions at the same level and the data has a hierarchical structure. This makes binary logistic regression not ~~to be used~~ as it violates the assumption of independence of the residuals; rather a model which considers clustering effect should be used(8, 11). Multilevel logistic modeling separates the within-cluster effects from the between-cluster effects (26). Therefore, to assess the predictors associated with U5M, a non-weighted multilevel logistic regression model was used.

Bivariable multilevel logistic regression was used for each predictor variable to screen out the significant variables (p-value less than 0.2) for multivariable multilevel logistic models. Twenty predictor variables (3 of them were community-level predictor variables) passed to multivariable analysis. In multivariable analysis, a p-value less than 0.05 was considered a factor associated with U5M.

The first model fitted was the null model (intercept model) which contains the outcome variable only (under-five mortality status) with the cluster number. The intra-cluster correlation (ICC) was used to assess whether there was a random effect. ICC of 0.130 which means there is a 13% under-five mortality which is explained by between cluster difference and around 87% of the under-five mortality is explained by within-cluster difference, which was not negligible. The second model was fitted by using the outcome, the cluster and as a predictor variable the individual level only. The probability of U5M was predicted as a function of individual-level predictors.

For the third model the outcome variable, the cluster number, and the community level predictor variables were accounted for. Then the final model was fitted with both the individual level and the community level predictor variables taken into account.

The models were compared by using a log-likelihood statistic, where the fitted model had the smallest -2log likelihood value that is deviance.

Model performance was checked by using residual diagnostic plot in R software package.

Ethical consideration

Permission for data access was obtained from a major demographic and health survey through an online request from http://www.dhsprogram.com. The data used for this study were publicly available with no personal identifier.

Result

Socio-demographic ~~B~~ackground and maternal and child health factor

An overall ~~of~~ 5,695 live births ~~was encompassed~~ in the study, of which 2,942 (51.66%) were male, 5,528 (97.07%) were single children, 2,937 (51.57%) were delivered at home and around 6.04% of the children were born via cesarean section (**Table 1**)

Out of the total children's mothers, most were married 5,346 (93.87 %) and 3,135 (55.05%) have no education, only 291(5.11%) attain higher education, and ~~Greater than~~ 51.62% ~~of the respondents~~ are of poor economic status. The majority of the respondents 64.27% ~~are utilizers of~~ improved water ~~and~~ 30.99 % travel more than 30 minutes, 50.85% travel less than 30 min to get water unlike those who have water on-premises. The majority of sex of household head is male 4,561 (80.09%) and the commonest age of household head is 35-50 ~~with~~ 45.58%. ~~Background of community factors~~ (**Table 1**)

Among the population, the majority ~~of the participants~~ were rural residents ~~with~~ 4389(77.07%). And 3457 (60.70%) of the community practiced home delivery. The number of communities that utilize unimproved toilets is 3175(55.75%). 3391 of the population (59.54%) are not exposed to the media. ~~And the majority of the children reside in rural areas~~ (**Table 2**)

Prevalence of under-five mortality

The under-five mortality is 5.9% ~~with its 95%~~ (CI 5.4%, 6.6%) and it varies across regions ranging from 2.13% in Addis Ababa to 9.82 in the Somali region. Out of the total 918(15.79%) ~~of~~ children who had a history of lower than 6 months of breastfeeding died (Table 3)

Spatial autocorrelation

The global Moran's index was 0.09, z-score 3.5, p-value <0.001 suggesting that the spatial distribution under-five mortality is not at random in the 2019 EMDHS (**Figure 2**)(27)

Hotspot analysis

A high proportion of U5M was observed around the regions of Somali and Dire Dawa (**Figure 3**)(27)

Spatial Interpolation

The spatial interpolation or prediction analysis result showed that the under-five mortality ranges from 0% to 50%. The high risk predicted areas were located in Somali, Dire Dawa, Benishangul Gumuz, Gambella, and SNNPR (**Figure 4**)(27)

Spatial scan statistics analysis

As observed in the figure 5 significant clusters were identified in the Somali region. Located within 7.384788 N, 45.908936 E with 612.75 km radius. Children living in the primary cluster were 62% more likely to die before they celebrate their fifth birthday when compared to those who lived outside the window (relative risk (1.62), Likelihood ratio (7.36), and p-value (0.03)) (**Figure 5**)(27)

Factors associated with Under-five mortality

~~By using multilevel logistic regression,~~ after filtering all variables in bivariable multilevel logistic regression, 4 models were fitted (null model, individual-level model, community-level model, and both individual and community level). The ICC was found to be 0.13 (95% CI: (0.08, 0.2)) which is not negligible. The 4th model was the best-fitted model with the lowest deviance. When the number of household members increases by one, the odds of under-five mortality will be decreased by 8.5% (AOR=0.09, 95%CI: (0.84, 0.99)). The odds of under-five death will be decreased by 82.8% (AOR=0.17, 95%CI: 0.14, 0.21) as the number of under-five children in the family increases by one. Children who ~~have~~ multiple births have 14 times higher odds of under-five mortality (AOR=14.4, 95%CI: (8.5, 24.3)) than singletons. Children who breastfed for less than 6months (AOR=5.04, 95%CI: (3.81, 6.67)) have 5 times higher odds of experiencing under-five mortality than those who have breastfed. People whose main roof is palm/bamboo have 43% (AOR=0.57, 95%CI: (0.34, 0.96)) decreased odds of having under-five deaths in the family than that of corrugated iron. Under-five children who are in the sixth birth order and more ~~are~~ 2.5 (AOR=2.46, 95%CI: (1.49, 4.06)) times higher odds ~~to have~~ under-five mortality as compared with second and third birth order. Place of delivery was found to have a significant association with U5M, those who have delivered in institutions have 43% less chance of experiencing U5M (AOR=0.57, 95%CI: (0.41, 0.81)) as compared with those delivered at home. Being a resident of

the Somali and Afar region increases the odds of under-five death by 3.5 (AOR=3.46, 95%CI: ~~(~~1.58, 7.55~~)~~) and 2.5 (AOR=2.54, 95%CI: ~~(~~1.10, 5.85~~)~~) times respectively as compared with the Tigray region (Table 4).

The residual plot shows that the model performs adequately, with the KS test being insignificant (p-value = 0.29), and also the deviation was not significant (**Figure 6**).

Discussion

The study mainly focused on the spatial distribution of under-five mortality and on factors that influence it. The findings of this study revealed that the prevalence of under-five mortality was ~~59 per 1000 live births with (95% CI: (54, 66)).~~ The multivariable multilevel logistic regression model result showed that family size, number of under-five children, birth order, breastfeeding, multiple birth, region, and institutional delivery were associated with under-five mortality in Ethiopia. The spatial analysis result revealed that Somali, Dire Dawa and Eastern Oromia were high-risk areas for under five mortalities in Ethiopia.

~~The study showed that under-five mortality in Ethiopia is 59 per 1000 live births with (95% CI: (5.4%, 6.6%)). This finding is still below the sustainable development goal target of 25 or low deaths per 1000 live births also it is not consistent with studies conducted in Ethiopia(8, 16), this difference might be due to the survey time of the data used and the sample size.~~

Increased number of ~~households~~ (family size) resulted in decreased odds of under-five death. It is in line with a study in Ethiopia(17), and Eastern Nigeria(28). This can be explained by, as the family size increases, the mother of the children will be more experienced in taking good and adequate care of the children and as well as there will be many family members that can care for under-five children.

As the number of under-five children in a family increase, the odds of under-five mortality will be decreased. This is in agreement with a study in Ghana (29). This is because the mother will be more experienced and she will learn from her previous mistakes if there is one. But there is a contradiction with another study result in Ethiopia, which demonstrated that the odds of under-five mortality increase as the number of under-five children increases(19). ~~This can be justified~~ as

the number of under-five children increases it will be difficult to fulfill their basic needs and their family (mother) will be exhausted to look after the children.

According to our study results being fourth and fifth or sixth and above-born children increase the odds of under-five mortality. This is in line with a study conducted in rural parts of Ethiopia (10). But it is not supported by a study conducted in Ethiopia which pointed out that being firstborn increases the odds of under-five mortality by two folds when compared to those with five and above birth orders(19). This can be because firstborns are more likely to be hospitalized as a result of congenital malformations and perinatal conditions in their early childhood. But, this changes for firstborn children as they get older, when younger brothers and sisters are more likely to be hospitalized for injuries and avoidable conditions, which shows less parental care (30).

Breastfeeding less than six months increases the odds of under-five mortality compared with those who breastfed for six and more months. ~~Which~~ is consistent with a finding in Ethiopia(19). This ~~can be justified by~~ breastfeeding ~~being~~ one of the prevention methods for reducing child mortality and it is a means of prevention of delayed growth (3[illegible]).

Being multiple birth children increases the odds of under-five mortality ~~when~~ in comparison to singleton children. This finding is in line with different studies conducted in Ethiopia at different times, a study conducted in rural parts of Ethiopia(7, 8, 10, 17, 19). This can be due to multiple births imposing short- and long-term medical complications (32).

Facility or institutional delivery reduces the odds of under-five mortality by 43% as compared to home delivery. This finding is consistent with studies conducted in Ethiopia(10, 11) and the Democratic Republic of Congo(14). This is ~~because; Home~~ delivery increases the death of under-five children due to a higher risk of postpartum complications ~~after the intrapartum~~, exposure to common vaccine-preventable diseases ~~as a result of~~ an absence of vaccination, and less likely to attend prenatal and postpartum visits (11, 33).

The odds of under-five mortality are higher for residents of Afar and Somali regions as compared to the Tigray region. Somali and Afar ~~have~~ the first and second lowest coverage of children with all basic vaccinations. ~~Children under five with symptoms of Acute Respiratory Infection and diarrhea are below the national average~~. There is serious child malnutrition with the first and second highest prevalence of wasting ~~for~~ Somali and Afar respectively(34, 35)~~ and~~ the Somali

region was one of the regions that were in the primary cluster from our spatial scan statistics analysis where under-five mortality is more likely to occur. This is not supported by studies conducted in Ethiopia based on the 2011 and 2016 EDHS(10, 11) where other regions are found to have a significant association with under-five mortality. This discrepancy can be a result of the different number of enumeration areas (clusters) which led to different samples, different statistical models used for analysis ~~(hurdle negative binomial mode1, multilevel negative binomial regression model, and binary logistic regression).~~

Conclusion: Under-five mortality in Ethiopia is ~~59 per 1000 live~~ births, which ~~are~~ high and unacceptable ~~as compared to the 2030 agenda for sustainable development aimed at 25 per 1000 live births.~~ Breastfeeding more than 6-month, twin birth, institutional delivery and high-risk areas of U5M (Somali and dire Dawa) were modifiable risk factors. Therefore, maternal education on the advantage of breastfeeding and institutional delivery is highly recommended. Women who deliver multiple births should be given special attention. An effective strategy should be designed for intervention for hot spot areas of U5M that ~~is~~ Somali and Dire Dawa.

Strength and limitation of the study

We used EMDHS 2019 data which is more inclusive and generalizable as EAs are taken from most parts of the country. The larger sample size increases the power of the study and helps to make geographical comparisons. As it is cross-sectional it does not show a temporal relationship ~~(chicken egg dilemma)~~, ~~important variables:~~ antenatal care ~~follow-up~~, postnatal care, and preceding birth interval were excluded from the study due to their ~~highest~~ rate of missing values.

Abbreviation

AOR: Adjusted Odds Ratio, CI: Confidence Interval, CS: Cesarean Section, EAs: Enumeration Areas, DHS: Demography and Health Survey, EMDHS: Ethiopia Mini Demography and Health Survey, HHs: households, ICC: Intraclass Correlation, KR: kids record, SGD: Sustainable Development Goal, U5M: under-five mortality, U5MR: under-five mortality rate

Declaration

Ethics approval and consent to participate

Ethical clearance was obtained from measure DHS through filling requesting a form for accessing data. The data used in this study are publicly available, aggregated secondary data that hasn't any personal identifying information that can be linked to study participants. The confidentiality of data was maintained anonymously.

Availability of data and materials

The datasets used during the current study are publicly available ~~data~~ from measure DHS program found at www.dhsprogram.com

Funding

The authors received no specific funding for this work

Acknowledgment

We acknowledge the DHS program for providing us with the dataset.

Consent for publication

Not applicable as there are no image or other confidentiality-related issues.

Competing interests

The authors declared that they have no competing interests.

Authors' contributions

LGG, TMT, and ZTT conceived the study, data analysis, drafted the manuscript, and critically reviewed the manuscript. Read and approve the final version of the manuscript.

Reference

1. Croft T, Marshall A, Allen C. Guide to DHS Statistics. Rockville, Maryland, USA: ICF; 2018. 2018.

2. Vidak I. OLDER PEOPLE AND SOCIAL MEDIA USAGE. Strategic Approach to Aging Population: Experiences and Challenges: Sveučilište Josipa Jurja Strossmayera u Osijeku, Ekonomski fakultet u Osijeku. p. 325-40.
3. Woldeamanuel BT, Aga MA. Count models analysis of factors associated with under-five mortality in Ethiopia. Global Pediatric Health. 2021;8:2333794X21989538.
4. Ezbakhe F, Pérez-Foguet A. Child mortality levels and trends. Demographic Research. 2020;43:1263-96.
5. Johnston R. Arsenic and the 2030 Agenda for sustainable development. Arsen Res Glob Sustain-Proc 6th Int Congr Arsen Environ AS. 2016;2016:12-4.
6. Li Z, Hsiao Y, Godwin J, Martin BD, Wakefield J, Clark SJ, et al. Changes in the spatial distribution of the under-five mortality rate: Small-area analysis of 122 DHS surveys in 262 subregions of 35 countries in Africa. PloS one. 2019;14(1):e0210645.
7. Fenta SM, Fenta HM, Ayenew GM. The best statistical model to estimate predictors of under-five mortality in Ethiopia. Journal of Big Data. 2020;7(1):1-14.
8. Worku MG, Teshale AB, Tesema GA. Determinants of under-five mortality in the high mortality regions of Ethiopia: mixed-effect logistic regression analysis. Archives of Public Health. 2021;79(1):1-9.
9. Ayele DG, Zewotir TT, Mwambi HG. Structured additive regression models with spatial correlation to estimate under-five mortality risk factors in Ethiopia. BMC public health. 2015;15(1):1-12.
10. Gebremichael SG, Fenta SM. Under-Five Mortality and Associated Risk Factors in Rural Settings of Ethiopia: Evidences from 2016 Ethiopian Demographic and Health Survey. Advances in Public Health. 2020;2020.
11. Geremew BM, Gelaye KA, Melesse AW, Akalu TY, Baraki AG. Factors Affecting Under-Five Mortality in Ethiopia: A Multilevel Negative Binomial Model. Pediatric Health, Medicine and Therapeutics. 2020;11:525.
12. Ghilagaber G, Antai D, Kandala N-B. Modeling spatial effects on childhood mortality via geo-additive Bayesian discrete-time survival model: A case study from Nigeria. Advanced techniques for modelling maternal and child health in Africa: Springer; 2014. p. 29-48.
13. Singh A, Masquelier B. Continuities and changes in spatial patterns of under-five mortality at the district level in India (1991–2011). International journal of health geographics. 2018;17(1):1-18.
14. Kandala N-B, Mandungu TP, Mbela K, Nzita KP, Kalambayi BB, Kayembe KP, et al. Child mortality in the Democratic Republic of Congo: cross-sectional evidence of the effect of geographic location and prolonged conflict from a national household survey. BMC public health. 2014;14(1):1-9.
15. Anafcheh T, Yaghoubi Doust M, Mojadam M, Mirkazemi R, Khafaie MA. Temporal and spatial distribution of under-five mortality and factors associated with multiple cases of under-five deaths within a family in the rural area of Khuzestan, Southern Iran. Scientific reports. 2018;8(1):1-8.
16. Ayele DG, Zewotir TT. Childhood mortality spatial distribution in Ethiopia. Journal of Applied Statistics. 2016;43(15):2813-28.
17. Dejene T, Girma E. Social determinants of under-five mortality in Ethiopia: Event history analysis using evidence from Ethiopian Demographic and Health Survey (EDHS). 2013.
18. Fikru C, Getnet M, Shaweno T. Proximate determinants of under-five mortality in Ethiopia: using 2016 nationwide survey data. Pediatric health, medicine and therapeutics. 2019;10:169.
19. Zewudie AT, Gelagay AA, Enyew EF. Determinants of under-five child mortality in Ethiopia: analysis using Ethiopian demographic health survey, 2016. International Journal of Pediatrics. 2020;2020.
20. Abir T, Agho KE, Page AN, Milton AH, Dibley MJ. Risk factors for under-5 mortality: evidence from Bangladesh Demographic and Health Survey, 2004–2011. BMJ open. 2015;5(8):e006722.
21. Pretorius CE, Asare H, Genuneit J, Kruger HS, Ricci C. Impact of breastfeeding on mortality in sub-Saharan Africa: a systematic review, meta-analysis, and cost-evaluation. European Journal of Pediatrics. 2020;179(8):1213-25.

22. Ethiopian Public Health Institute - EPHI, Federal Ministry of Health - FMoH, ICF. Ethiopia Mini Demographic and Health Survey 2019. Addis Ababa, Ethiopia: EPHI/FMoH/ICF; 2021.
23. Fotheringham ASRP. The SAGE handbook of spatial analysis. Los Angeles; London: SAGE Publications; 2009.
24. Bhunia G, Shit P, Maiti R. Comparison of GIS-based interpolation methods for spatial distribution of soil organic carbon (SOC). J Saudi Soc Agric Sci 17: 114–126. 2018.
25. Kulldorff M. A spatial scan statistic. Communications in Statistics-Theory and methods. 1997;26(6):1481-96.
26. Sommet N, Morselli D. Keep calm and learn multilevel logistic modeling: A simplified three-step procedure using stata, R, Mplus, and SPSS. International Review of Social Psychology. 2017;30:203-18.
27. GeoPortal I. OpenAfrica shapfile 2019 [Available from: https://open.africa/dataset/africa-shapefiles.
28. Azuike E, Onyemachi P, Amah C, Okafor K, Anene J. Determinants of under-five mortality in South-Eastern Nigeria. J Community Med Public Health Care. 2019;6:049.
29. Aheto JMK. Predictive model and determinants of under-five child mortality: evidence from the 2014 Ghana demographic and health survey. BMC public health. 2019;19(1):1-10.
30. Julihn A, Soares FC, Hammarfjord U, Hjern A, Dahllöf G. Birth order is associated with caries development in young children: a register-based cohort study. BMC Public Health. 2020;20(1):1-8.
31. Ahmed AH. The International Code of Marketing of Breast-milk Substitutes: Update on the Global Implementation. Journal of Human Lactation. 2020;36(4):803-7.
32. Elster N. Less is more: the risks of multiple births. Fertility and Sterility. 2000;74(4):617-23.
33. Goyal S, Kortsmit K, Cox S, D'Angelo DV, Romero L, Henderson ZT, et al. Prevalence of Home Births and Associated Risk Profile and Maternal Characteristics, 2016–2018. Obstetrics & Gynecology. 2020;136(6):1195-203.
34. Dessalegn M, Ayele M, Hailu Y, Addisu G, Abebe S, Solomon H, et al. Gender inequality and the sexual and reproductive health status of young and older women in the Afar region of Ethiopia. International Journal of Environmental Research and Public Health. 2020;17(12):4592.
35. Oladeji O, Oladeji B, Omer MD, Farah AE, Ameda IM, Gera R, et al. Exploring opportunities to enhance effectiveness of mobile health and nutrition strategy for providing health and nutrition services amongst pastoralists in Somali region, Ethiopia. African Journal of Primary Health Care & Family Medicine. 2021;13(1):1-7.

List of Figures

Figure 1:- conceptual framework of under-five mortality in Ethiopia 2019(27).

Figure 2:-Spatial autocorrelation of under-five mortality in Ethiopia 2019(27).

Figure 3:- Hotspot analysis of under-five mortality in Ethiopia 2019(27).

Figure 4:- Empirical Bayesian Kriging prediction map of under-five mortality in Ethiopia 2019(27).

Figure 5:- Spatial scan statistics of under-five mortality in Ethiopia 2019(27).

Figure 6:- residual diagnostic plot for the final model of under-five mortality in Ethiopia 2019.

Table 1 Sociodemographic, maternal, and child health factors

Sociodemographic, Maternal and child health factors		Frequency	Percentage
Age of the mother at first birth	Less than 18	3,024	53.10%
	19 upto 34	2,650	46.53%
	35 and more	21	0.37%
Sex of child	Male	2,942	51.66%
	Female	2,753	48.34%
Contraceptive utilization	not use	3,875	68.04%
	use	1,820	31.96%
Multiple births	Singleton	5,528	97.07%
	Multiple	167	2.93%
Birth order	1st order	1,228	21.56%
	2nd and 3rd order	1,883	33.06%
	4th and 5th order	1,298	22.79%
	6th order	1,286	22.58%
Place where the mother delivered	home delivery	2,937	51.57%
	Facility delivery	2,758	48.43%
Delivery by cesarean section	No	5,351	93.96%
	yes	344	6.04%
Months of Breastfeed	Less than 6month	918	16.12%
	6 month and grater	4,777	83.88%
Sex of household head	Male	4,561	80.09%
	Female	1,134	19.91%
Age of household head	less than 34	2,479	43.53%
	35 up to 50	2,596	45.58%
	50 and more	620	10.89%

Marital Status	Married	5,346	93.87%
	Unmarried	349	6.13%
Highest educational level	No education	3,135	55.05%
	Primary	1,802	31.64%
	Secondary	467	8.20%
	Higher	291	5.11%
Wealth index	Poor	2,940	51.62%
	Middle	797	13.99%
	Rich	1,958	34.38%
Water source	Improved	3,660	64.27%
	Unimproved	2,035	35.73%
Time to fetch water	more than 30 min	1,765	30.99%
	less than 30 min	2,896	50.85%
	On premises	1,034	18.16%
Type of latrine used	Improved	1,164	20.44%
	Not improved	4,531	79.56%
Household has electricity	No	4,073	71.52%
	Yes	1,622	28.48%
Cooking fuel in use	Clean fuel	432	7.59%
	Solid fuel	5,263	92.41%
Type of floor of the house	Earth/sand/dung	4,384	76.98%
	Wood	49	0.86%
	Cement	799	14.03%
	Carpet	463	8.13 %
Type of the house wall	Cane/Palm/Trunk/Reed	1,261	22.14%
	Bamboo/wood	3,531	62.00%
	Cement/stone with lime/bricks/covered adobe	903	15.86%

Type of ~~the~~ house roof	Thatch	1,488	26.13%
	Palm/bam	995	17.47%
	Cement	198	3.48%
	corrugated iron	3,014	52.92 %

Table 2 Community-level ~~factors~~

Community level ~~factors~~		frequency	Percentage
Place of residence	Urban	1,306	22.93%
	Rural	4,389	77.07%
Community place of delivery	Home delivery	3,457	60.70%
	Facility delivery	2,238	39.30%
Community toilet utilization	Improved	2,520	44.25%
	Not improved	3,175	55.75%
Community wealth status	Poor	2,776	48.74%
	Rich	2,919	51.26%
Community media exposure	Not exposed to media	3,391	59.54%
	Exposed to media	2,304	40.46%

Table 3 Under-five mortality among predictors

Characteristics		Is the child alive?		total
		Yes 5357(94.06%)	No 338(5.94%)	
Is child twin?	Yes	123	44	167
	No	5234	294	5,528
Region	Tigray	436	14	450

	Afar	616	35	651
	Amhara	478	23	501
	Oromia	674	41	715
	Somali	580	57	637
	Benishangul	477	45	522
	SNNPR	631	26	657
	Gambella	409	35	444
	Harari	408	29	437
	Addis Ababa	281	6	287
	Dire Dawa	367	27	394
Breastfeeding	Less than 6 months	773	145	918
	6 months and more	4,584	193	4,777
Birth order	1st order	1,142	86	1,228
	2-3rd order	1,793	90	1,883
	4-5th order	1,231	67	1,298
	6th + order	1,191	95	1,286
Place of delivery	Home delivery	2,733	204	2,937
	Facility delivery	2,624	134	2,758

Table 4 Multivariable multilevel logistic regression analysis of both individual and community-level factors associated with under-five mortality in Ethiopia, EMDHS 2019.

Variable	Null model	Model2 AOR(95%CI)	Model3 AOR(95%CI)	Model4 AOR(95%CI)
Education				
No education		1		1
Primary		1.14 (0.83,1.56)		1.14 (0.83,1.58)
Secondary		0. .59 (0.31, 1.13)		0.58(0.30,1.12)

Higher		0. .65(0. .28, 1.55)		0.60(0.25,1.44)
Household members		0.922(0. 85, 1.01)		0.92(0.83,0.99) *
Number of under-five children		0.17 (0.14, 0 .22)		0.17(0.14, 0.21) ***
Sex				
Male		1		1
female		0 .83(0 .64, 1.07)		0.84(0.64,1.09)
Mode of delivery by C/S				
No		1		1
Yes		1.36 (0. 79, 2.35)		1.37(0.79,2.39)
Preceding birth order				
1st		1		1
2-3rd		1.32(0.92, 1.91)		1.34(0.93,1.94)

446

4-5th		1.52(0.97, 2.39)		1.51(0.96,2.38)
6+		2.44(1.48, 4.02)		2.46(1.49,4.06) ***
Time to water				
>30min		1		1
<30min		0.77(0.56, 1.04)		0.89(0.64,1.23)
On-premises		0.66(0.38, 1.14)		0.76(0.43,1.34)
Cooking fuel				
Clean fuel		1		1
Solid fuel		1.27(0.62, 2.58)		0.93(0.45,1.94)

Age at 1st birth				
19-34		1		1
<=18		0.91(0.70, 1.20)		0.90(0.68,1.18)
>=35		2.58(0.55, 12.01)		2.72(0.55,13.59)
Breastfeeding				
< 6 months		1		1
>= 6 months		5.09(3.85, 6.72)		5.05(3.82,6.67) ***
Place of delivery				
Home delivery		1		1
Facility delivery		0.54 (0.40,0.75)		0.57(0.41,0.81) **
Main floor				
earth/sand/dung		1		1
Wood		1.71(0.53, 5.52)		2.14(0.66,6.95)
Cement		0.89(0.50, 1.59)		0.86(0.48,1.58)
Carpet		1.56(0.85, 2.86)		1.28(0.67,2.46)
Main wall				
bamboo/wood		1		1
cane/palm/trunks/reed		1.42(0 .97, 2.07)		1.06(0.70,1.58)
cement/stone		1.53(0.89,2.61)		1.34(0.76,2.34)
Main roof				
Corrugated iron		1		
thatch		1.25(0.87,1.79)		1.14(0.77,1.67)
palm/bamboo		0.71(0.44,1.15)		0.57(0.34,0.96)*
calamine/cement		1.14(0.54,2.44)		1.04(0.47,2.29)
Contraceptive utilization				

No		1		1
Yes		0.72(0.53,0.98)		0.82(0.60,1.13)
Marital status				
Currently Married		1		1
Currently Unmarried		1.19(0.74,1.92)		1.22(0.75,1.97)
Multiple Twin child				
No		1		1
Yes		15.63(9.33,26.20)		14.39(8.51,24.33)***
Region				
Tigray			1	1
Afar			1.60(0.76,3.41)	2.55(1.10,5.85)*
Amhara			1.47(0.68,3.19)	0.97(0.44,2.15)
Oromia			1.73(0.84,3.55)	1.52(0.73,3.15)
Somali			2.86(1.39,5.89)	3.46(1.59,7.55)**
Benishangul gumuz			2.74(1.32,5.68)	1.95(0.91,4.20)
SNNPR			1.17(.55,2.47)	0.94(0.44,2.03)
Gambella			2.71(1.29,5.69)	1.94(0.90,4.21)
Harari			2.37(1.12,5.04)	1.95(0.87,4.36)

Addis Ababa			0.71(0.25,2.03)	0.64(0.21,2.02)
Dire Dawa			2.18(1.01,4.70)	2.02(0.91,4.50)
Community delivery place				
Home delivery			1	1
Facility delivery			0.75(0.52, 1.09)	0.94(0.62,1.43)
Community media				
Not exposed			1	1
exposed			1.09(0.75,1.58)	0.94(0.64,1.40)
Intercept	0.49(0.30,0.81)	0.22(0.08,0.63)	0.35(0.19,0.64)	0.16(0.04,0.57)
ICC	0.130	0.036	0.091	0.025
Log-likelihood	-1263.56	-918.15	-1247.40	-903.72

*** P-value<0.001, ** p-value<0.01, * p-value<0.05

Figure

Click here to access/download;Figure;Figure 1.tif

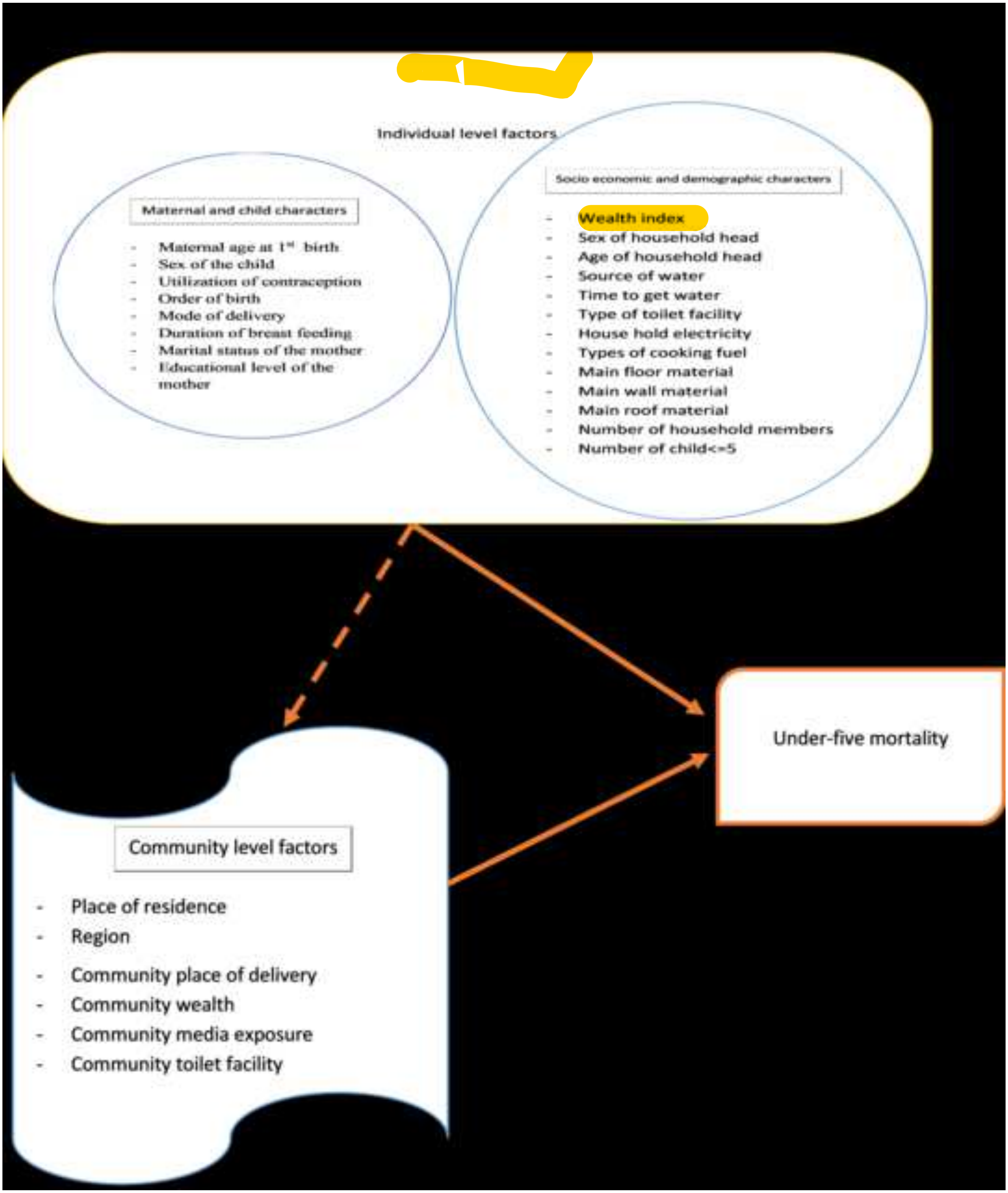

Figure

Click here to access/download;Figure;Figure 2.tif

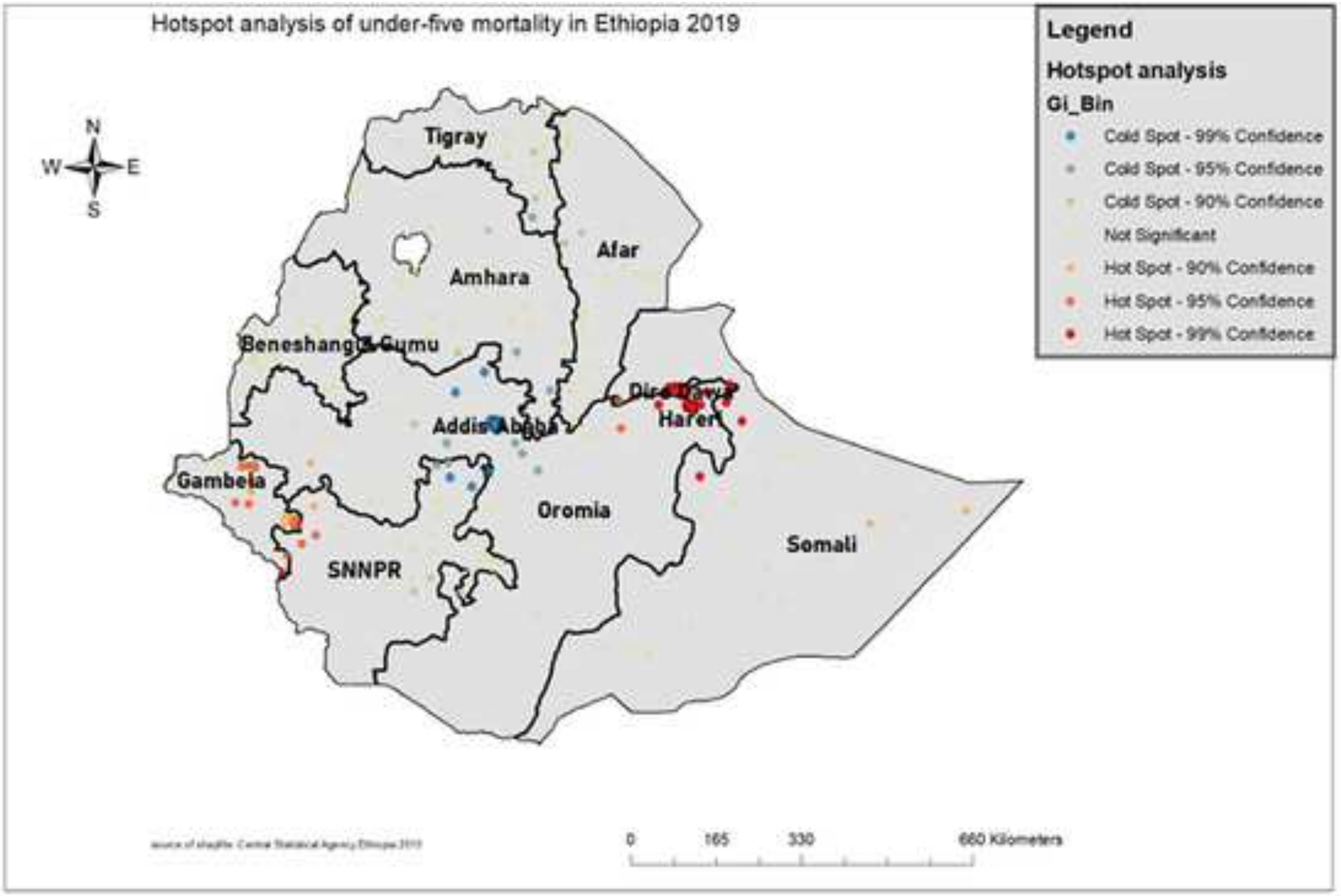

Figure

Click here to access/download;Figure;Figure 3.tif

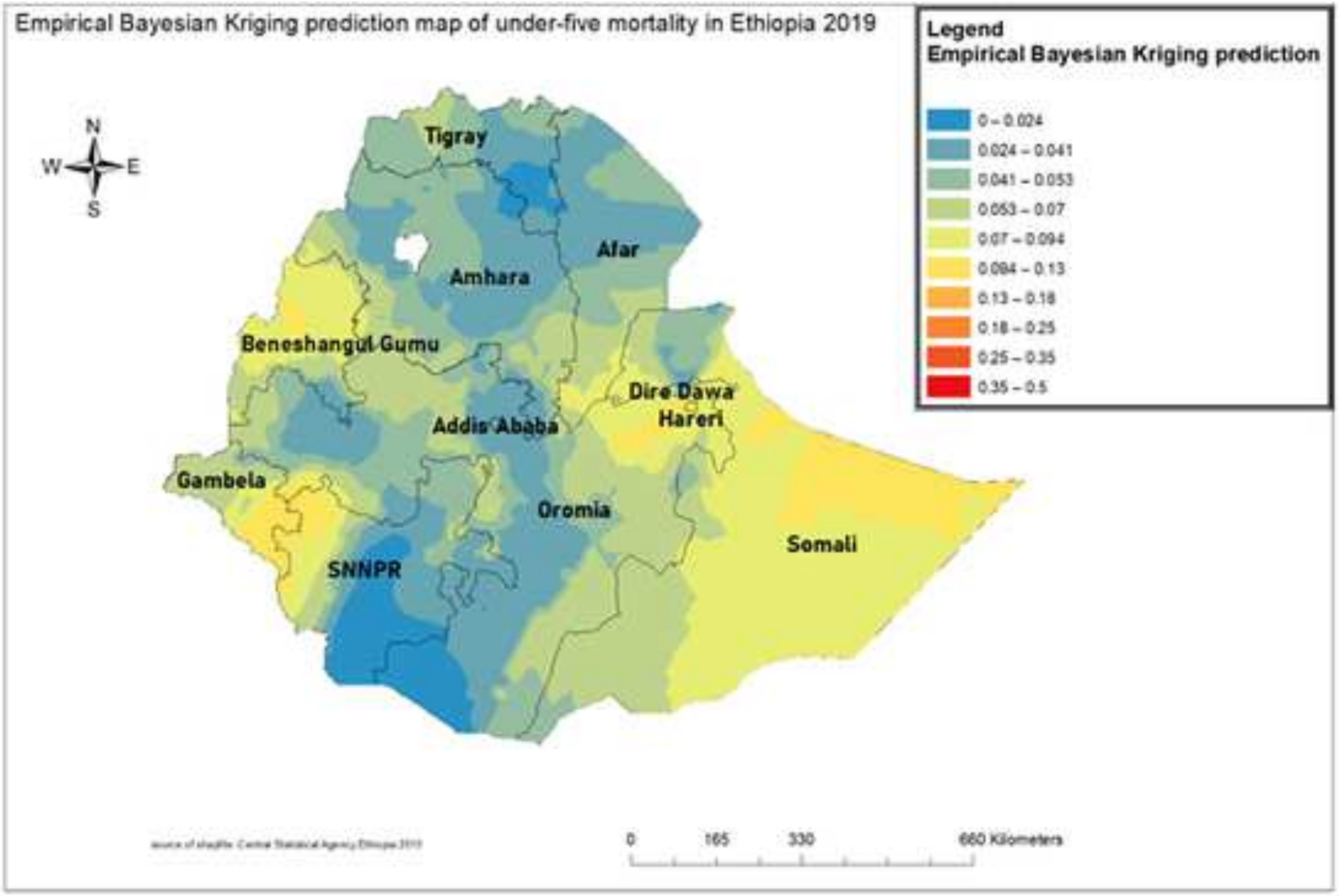

Figure

Click here to access/download;Figure;Figure 4.tif

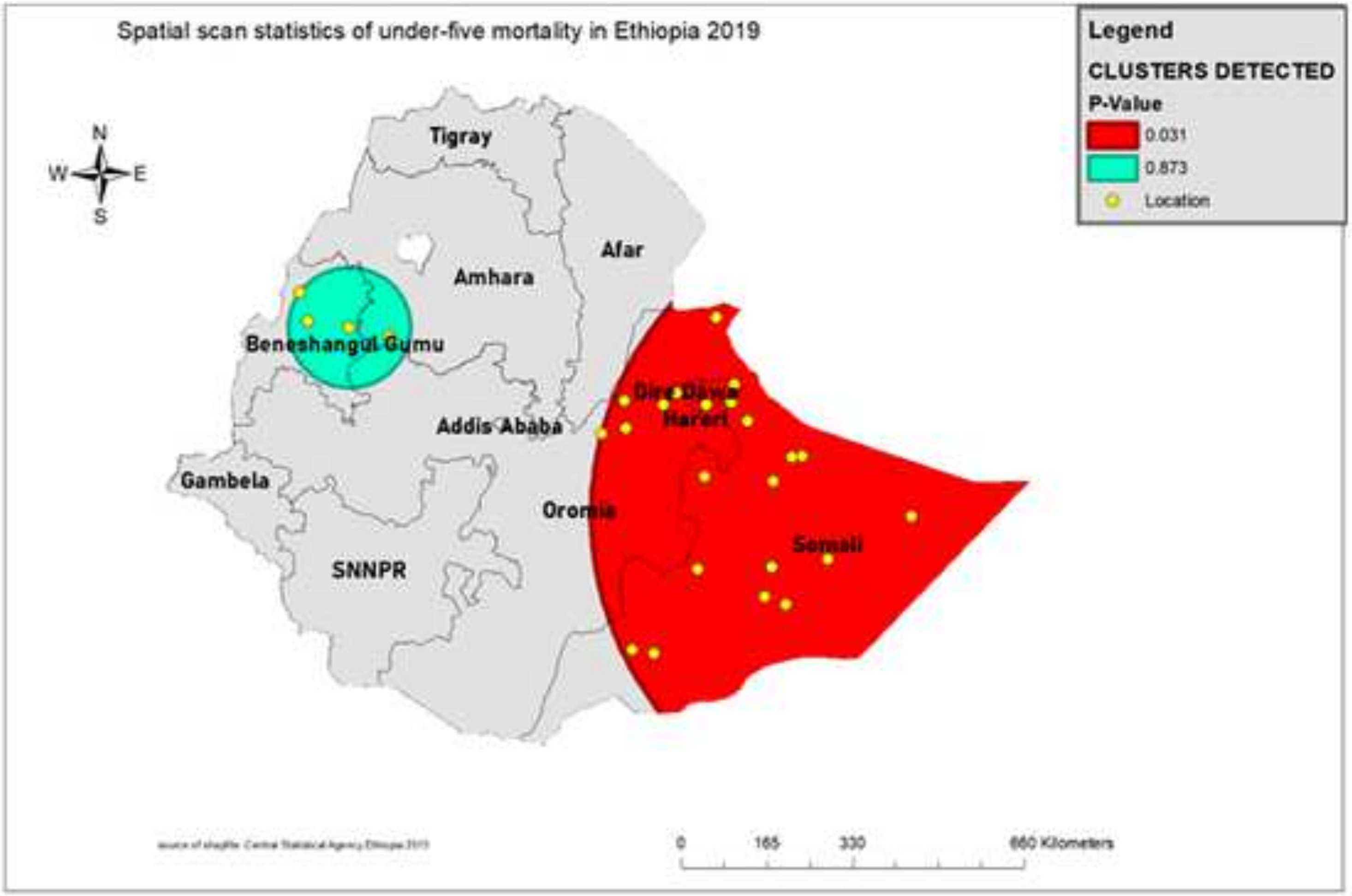

Figure

Click here to access/download;Figure;Figure 1.tif

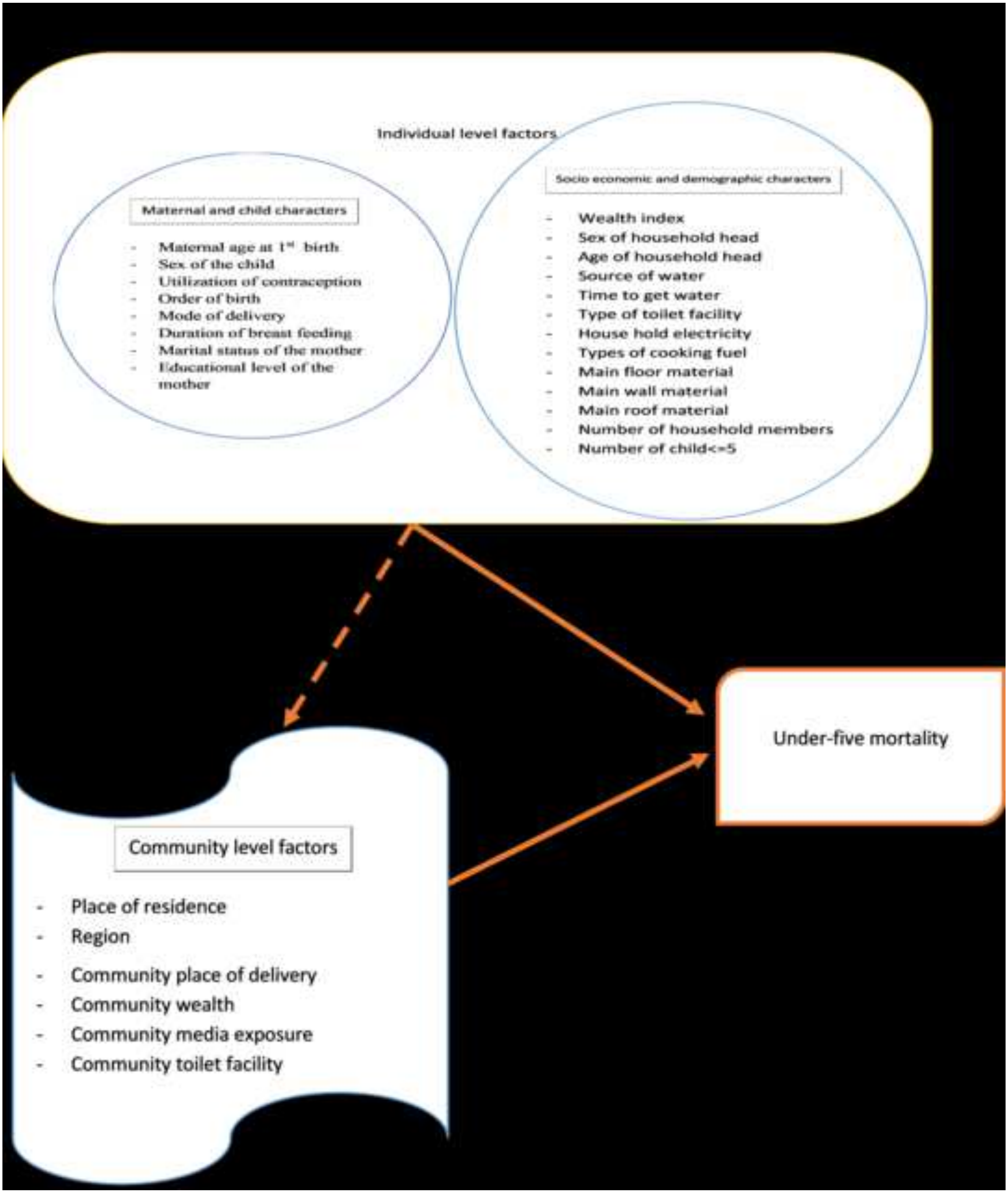

 Click here to access/download;Figure;Figure 2 (2).tif

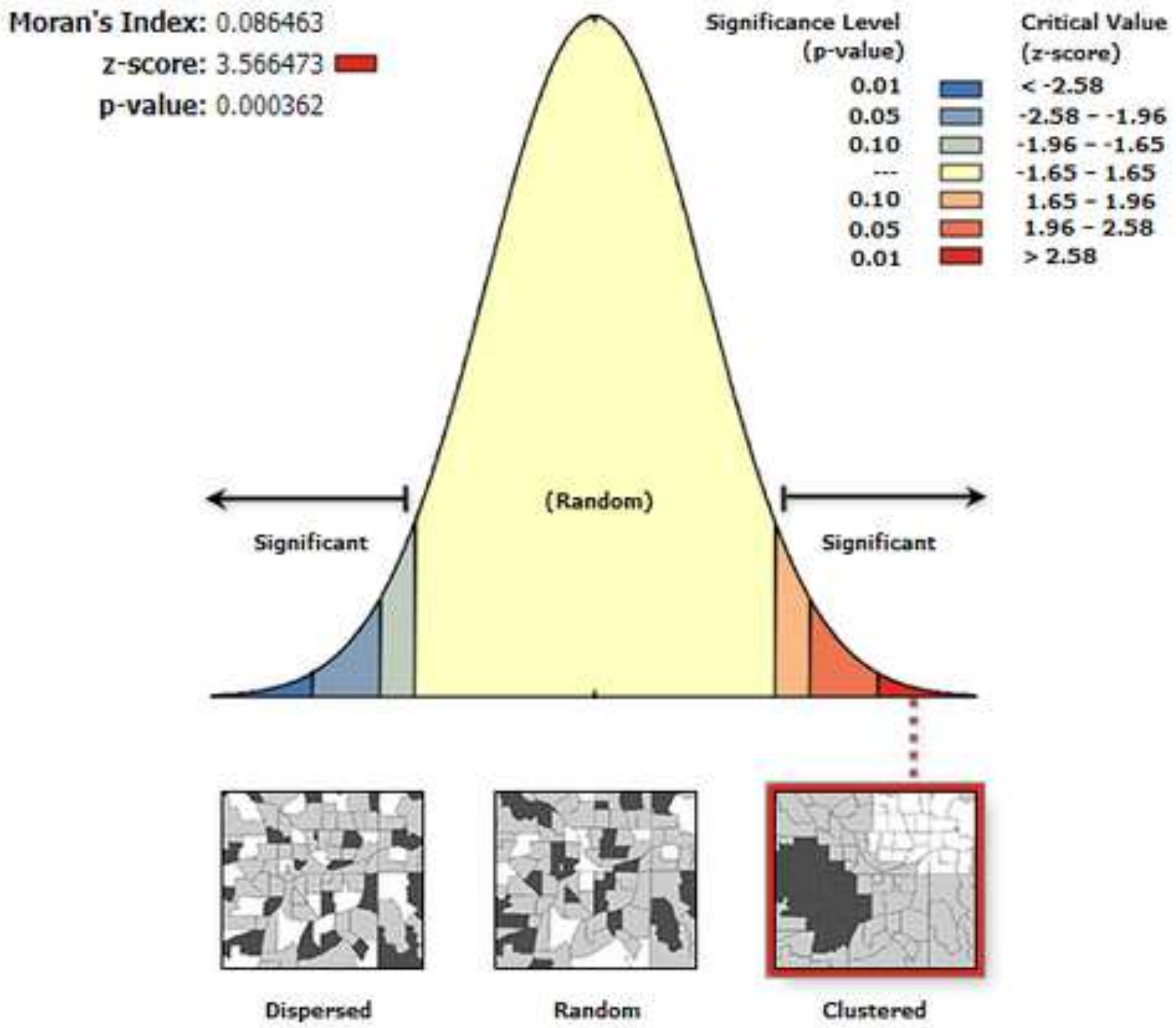

Figure

Click here to access/download;Figure;Figure 3 (2).tif

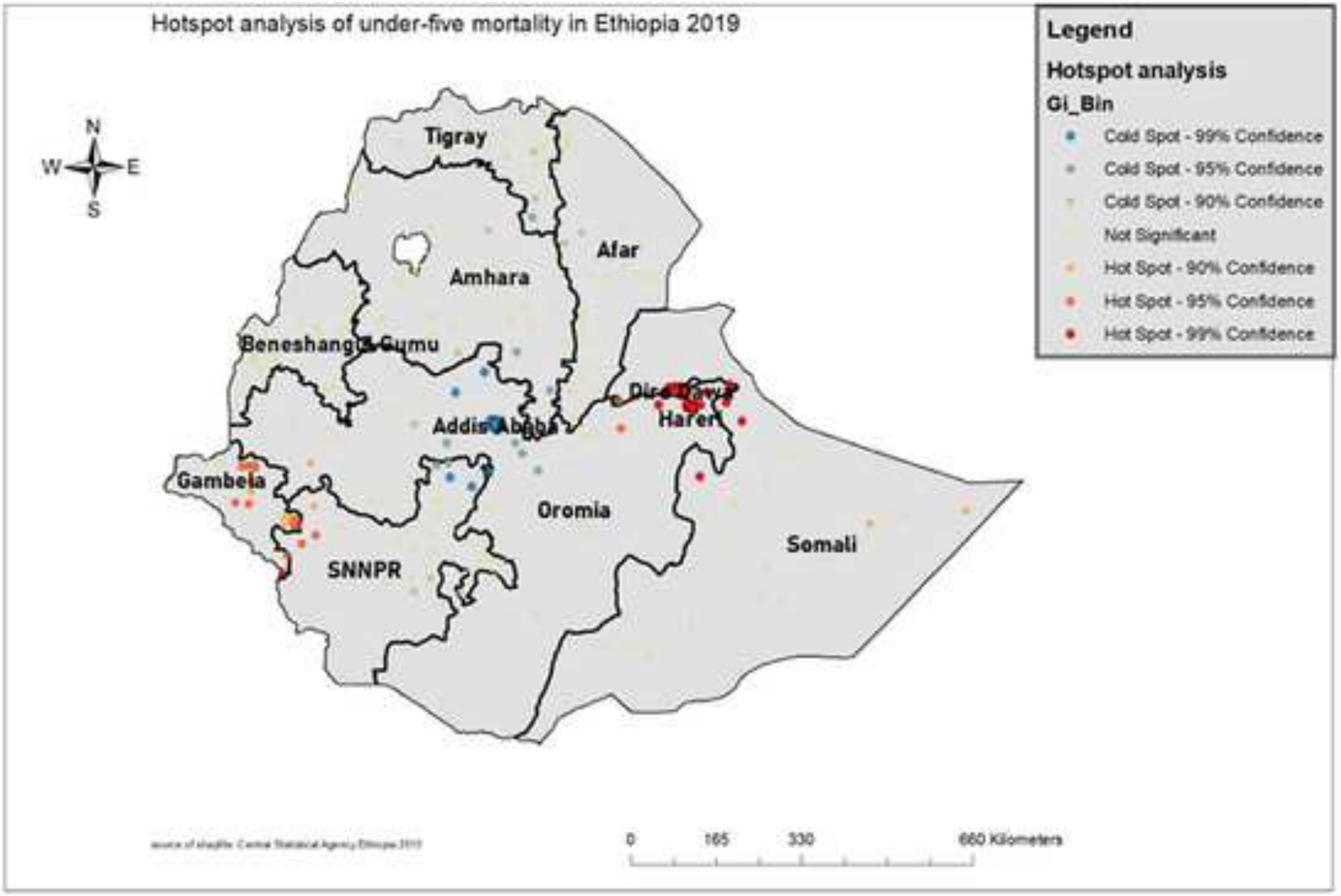

Figure

Click here to access/download;Figure;Figure 4 (2).tif

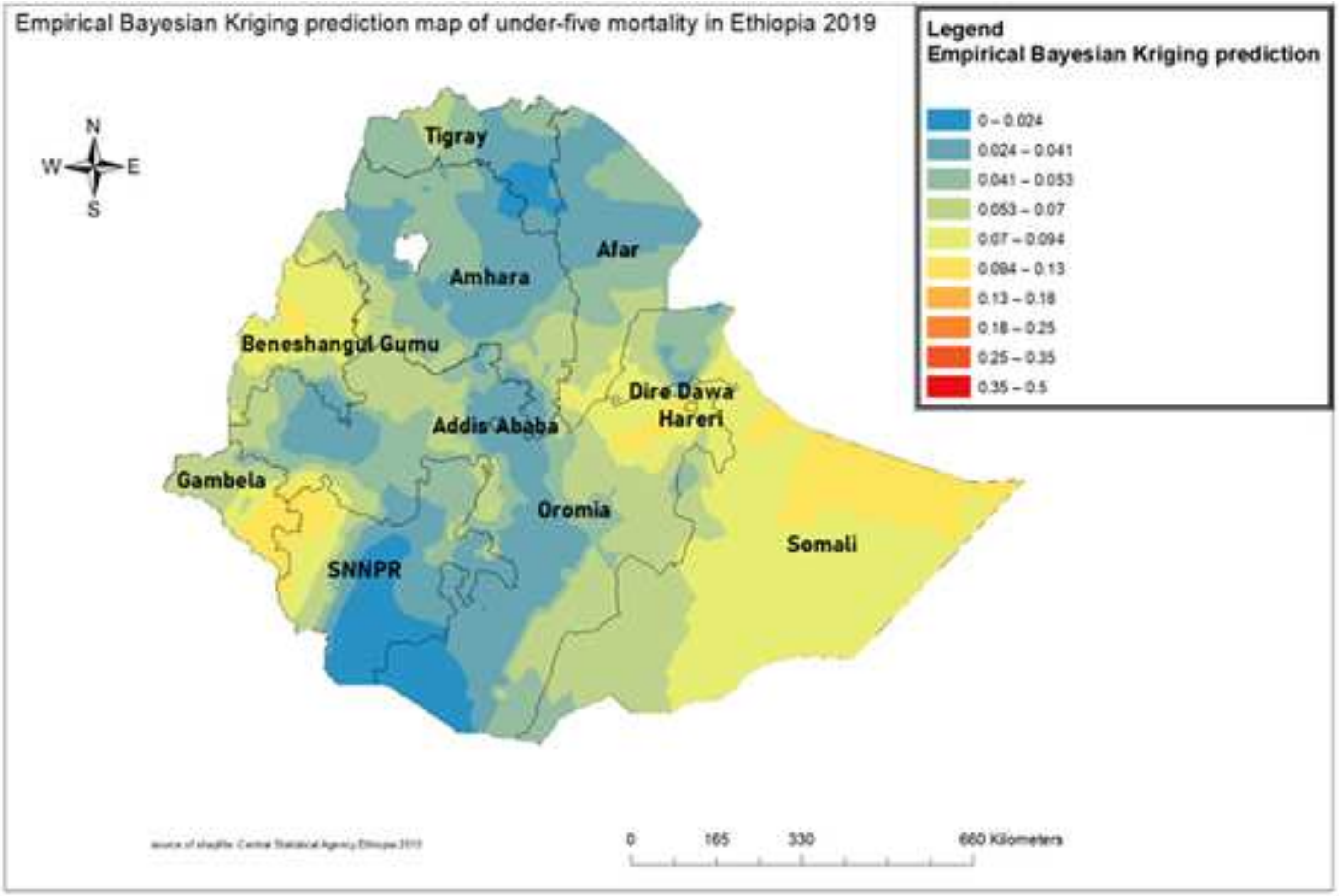

Figure

Click here to access/download;Figure;Figure 5 (2).tif

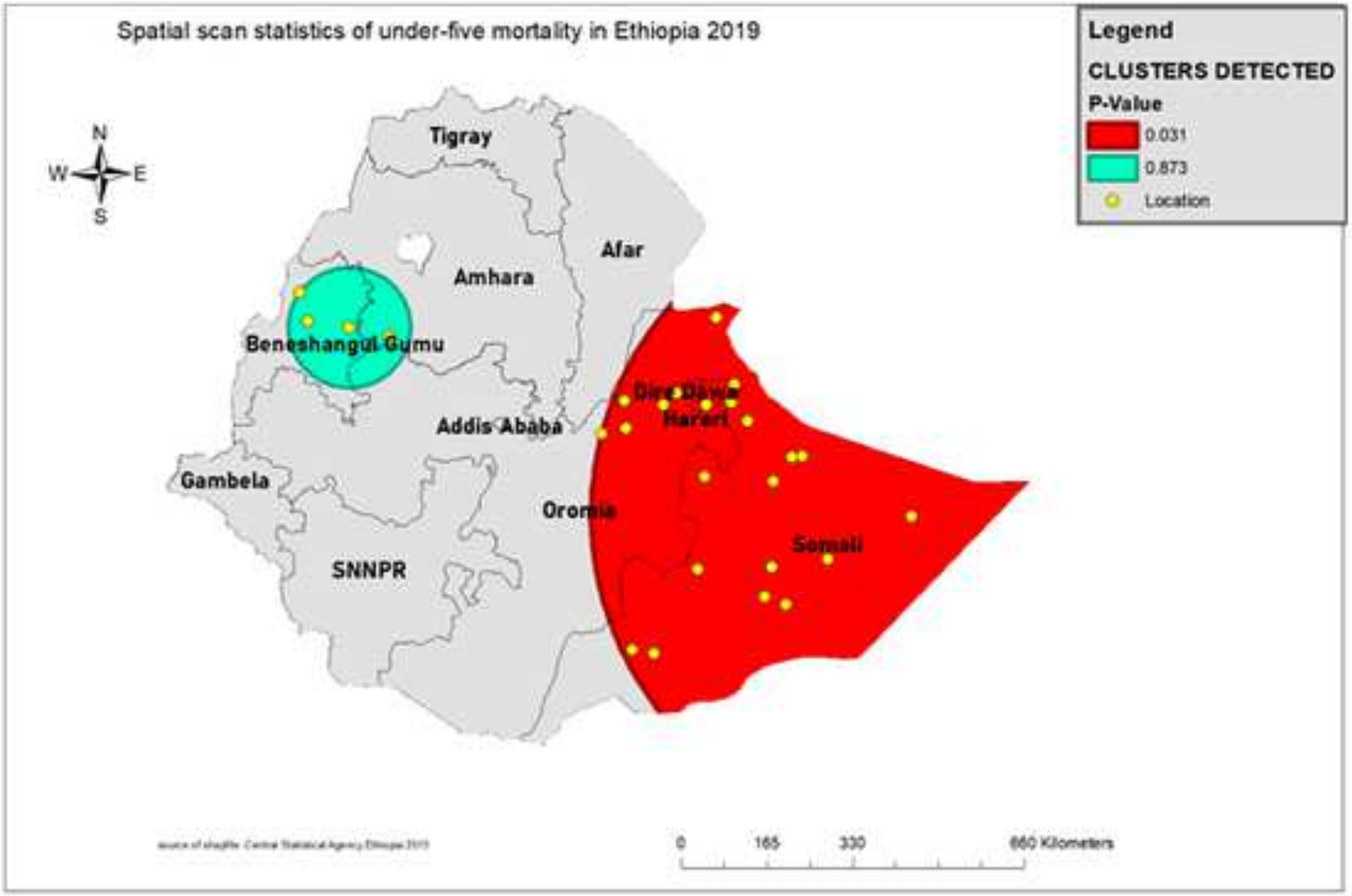

 Click here to access/download;Figure;Figure 6 (2).tif

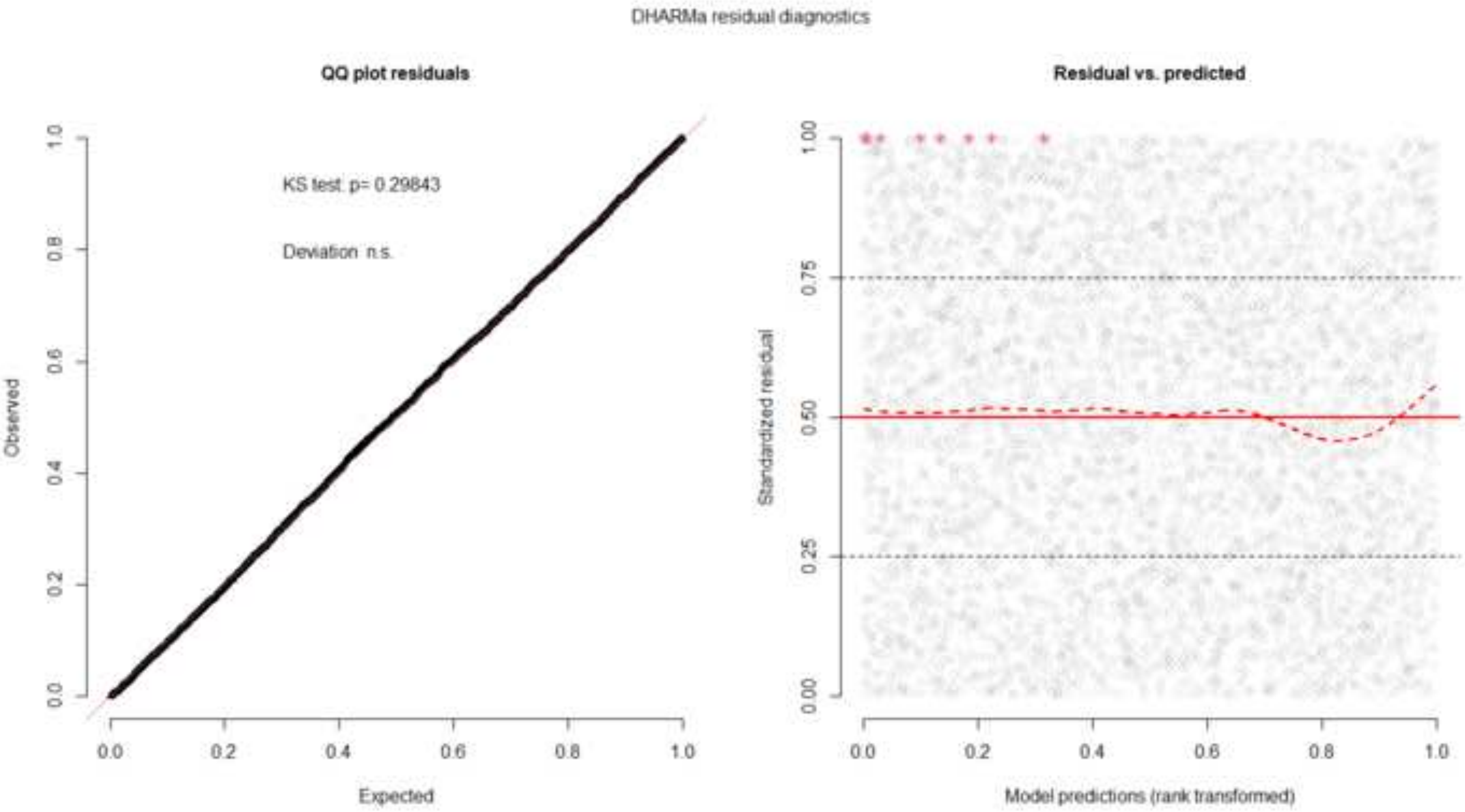

Revised Manuscript with Track Changes

Geographic variation and factors associated with under-five mortality in Ethiopia. A spatial and multilevel analysis of Ethiopian Mini Demographic and Health Surveys 2019.

Zemenu Tadesse Tessema [1*], Tsion Mulat Tebeje[2], Lewi Goytom Gebrehewet[1]

[1] Department of Epidemiology and Biostatistics, Institute of Public Health, College of Medicine and Health Sciences, University of Gondar, Gondar, Ethiopia

[2] Epidemiology and Biostatistics unitUnit, School of public health, Dilla university,University, Dilla, Ethiopia

E-mail : E-mail : ZTT zemenut1979@gmail.com

LGG lewigt369@gmail.com

TMT yemarina12@gmail.com

LGG lewigt369@gmail.com

ZTT zemenut1979@gmail.com

***= Corresponding author**

Formatted: Underline, Font color: Custom Color(RGB(17,85,204))

Formatted: Underline, Font color: Custom Color(RGB(17,85,204))

Abstract

Background: The distribution of under-five mortality (U5M) worldwide is uneven and the burden is higher in Sub-Saharan African countries which account for more than 53% of the global under-five mortality. In Ethiopia, though U5M shows a substantial decrease from 1990 to 2019, it is still ~~unacceptably~~excessively high and unevenly distributed. Therefore, this study aimed to assess geographic variation and factors associated with under-~~-~~five mortality in Ethiopia.

Method: We sourced data from the most recent nationally representative 2019 Ethiopian ~~mini~~ Mini-Demographic and Health Survey data for this study. A sample size of 5,695 live births ~~were~~was considered for this study. Descriptive and analytical Analysis was conducted by using STATA version 16. Both multilevel and spatial analyses were employed to ascertain the factors associated with under-five mortality in Ethiopia.

Result: The under-five mortality is 5.9% with its 95% CI 5.4% to 6.6%. Based on the multivariable multilevel logistic regression model result; family size (AOR=0.09, 95%CI :(0.84, 0.99)), number of under-five children in the family (AOR=0.17, 95%CI: 0.14, 0.21), multiple birth (AOR=14.4, 95%CI: (8.5, 24.3)), Children who breastfed for less than 6 months (AOR=5.04, 95%CI: (3.81, 6.67)), People whose main roof is palm (AOR=0.57, 95%CI: 0.34, 0.96), Under-five children who are at birth order sixth or more (AOR=2.46, 95%CI: (1.49, 4.06)), institutional delivery (AOR=0.57, 95%CI: (0.41, 0.81)), being resident of Somali and Afar region (AOR=3.46, 95%CI: (1.58, 7.55)) and (AOR=2.54, 95%CI: (1.10, 5.85)) respectively were factors associated with under-five mortality. A spatial analysis result revealed that hot spot areas of under-five mortality were located in the Somali region and Dire Dawa.

Conclusion: Under-five mortality in Ethiopia is 59 per 1000 live births which ~~is~~are high and unacceptable as compared to the 2030 agenda for sustainable development aimed at 25 per 1000

Formatted: Justified, Line spacing: 1.5 lines

Formatted: Font: Not Bold

Formatted: Font: Not Bold

Formatted: Font: Times New Roman, 12 pt

Formatted: Font: Not Bold

Formatted: Font: Not Bold

live births. ~~Breast feed~~Breastfeeding less than 6~~-~~-month, twin birth, institutional delivery and high -risk areas of under-five mortality (Somali and dire Dawa) were modifiable risk factors. Therefore, maternal education on the advantage of ~~breast feeding~~breastfeeding and institutional delivery is highly recommended. Women who deliver twins should be given special attention. An effective strategy should be designed for intervention for hot spot areas of under-five mortality that is Somali and Dire Dawa.

Keywords: Under~~-~~-five mortality, factors, spatial distribution, multilevel, Ethiopia

Formatted: Font: Not Bold

Background

Under-five mortality is defined as deaths reported at ages 0 to 59 months and it contains neonatal, post-neonatal, and child deaths (1). Under-five mortality rate (U5MR) is the probability of a child dying before celebrating the fifth birthday (2). It is a significant indicator of the socioeconomic, health, environmental conditions, and national development of health equity and access (3).

Globally, in 1990, under-five deaths were 12,494,000 (93 deaths per 1000 live births). By showing an average annual decline of 3.1%, in 2019 it was significantly reduced to 5,189,000 deaths (38 deaths per 1000 live births) with a 58% reduction (4). The 2030 agenda for sustainable development is aimed at ending the preventable deaths of newborns and children under 5 years of age, with all countries aiming to reduce under-5 mortality to make it at least less than 25 per 1000 live births (5).

The distribution of U5MR worldwide is uneven and has wider disparity and the global burden of U5MR is concentrated at two sustainable development goals (SDG) regions~~:~~, Sub-Saharan Africa and Central and Southern Asia with more than 80% of U5MR accounted for (6). The burden is higher in Sub-Saharan African countries with 74 deaths per 1000 live births, which accounts for more than 53% of the global under-five mortality (7).

In Ethiopia, U5MR shows a substantial decrease from 200 per 1000 live births ~~it was~~ in 1990 to 55 per 1000 live births in 2019 but still couldn't achieve the SDG aim (4~~). But a difference in administrative levels~~), additionally different studies conducted in Ethiopia revealed several Socioeconomic, demographic, and geographic or ~~regions and~~ spatial variation is considerable~~.~~, and Afar, Somali, and Benishangul Gumuz are the three high-~~-~~risk regions of Ethiopia for U5MR (8). ~~Different studies conducted in Ethiopia revealed several Socioeconomic, demographic, and geographic/spatial factors.~~

Different studies conducted on U5M previously showed that~~:~~ source of water (9), multiple ~~birth~~births (7)(8), region (10)(11), age and sex of household head (10), maternal education status (12)(13), place of delivery (10)(11)(14), maternal age (15)(16)(17), sex of the child (9)(14)(16), residence (18)(19), family size (17)(11), number of children under five (18)(20), birth order

Formatted: Font color: Black

Formatted: Normal, Border: Top: (No border), Bottom: (No border), Left: (No border), Right: (No border), Between : (No border), Tab stops: 3.25", Centered + 6.5", Right

Formatted: Justified

Formatted: Font color: Auto

Formatted: Font color: Black

Formatted: Normal, Border: Top: (No border), Bottom: (No border), Left: (No border), Right: (No border), Between : (No border), Tab stops: 3.25", Centered + 6.5", Right

(11)(18), and ~~breast feeding~~breastfeeding status (18)(21) have a significant association with under-five mortality.

Though U5M has been declining it is still high and is taking the lives of many children, and no research on U5M has been made on the recent survey data, Ethiopian mini~~-~~-Demographic Health Survey (EMDHS) 2019. ~~Identifying areas and factors with the highest burden of U5M in Ethiopia is helpful to show the current burden of U5M~~Therefore, to make different policies and interventions of U5M prevention with the efficient allocation of scarce resources according to spatial variability based on our findings and to achieve the SDG by understanding the determinants~~. Therefore,~~, identifying areas and factors with the highest burden of U5M in Ethiopia is helpful to show the current burden of U5M, hence this study is aimed at assessing the geographic variation and factors associated with Under-five mortality in Ethiopia.

Method

Data Source

~~Based on~~ Our source was the 2019 EMDHS~~, which~~ is the second mini demographic health survey (DHS) conducted ~~in Ethiopia next to the 2014 EMDHS, secondary data analysis was accompanied. Kids Record (KR) format was the data of interest to conduct the study. The study took place~~ in Ethiopia, which is a land-locked country located in the Horn of Africa that lies between the 30 N and 150N Latitude or 330 E and 480 E Longitude (22). Data collection was conducted from March 21, 2019, to June 28, 2019, from the nine regions (Tigray, Afar, Amhara, Oromia, Somali, Benishangul Gumuz, SNNPR, Harari, and Gambella) and two administrative cities (Addis Ababa and Dire Dawa). The study design was a population-based cross-sectional study. Kids Record (KR) format was the data of interest to conduct the study. A frame of all census Enumeration areas (EAs) ~~were~~was used as a sampling frame for the 2019 EMDHS. 149,093 EAs were created which covers an average of 131 Households (HHS). A two-stage stratified cluster sampling technique was employed and each region was stratified into urban and rural areas, yielding 21 sampling strata. Enumeration areas (EAs) were selected independently in each stratum. In the first stage, 305 clusters (93 urban and 212 rural) were selected with probability proportional to EAs size and with independent selection in each sampling stratum. In the second stage, a fixed number of 30 households per cluster were selected. Finally, women aged 15–49 in 9150 (2790 urban and 6360

Formatted: Font color: Black

Formatted: Normal, Border: Top: (No border), Bottom: (No border), Left: (No border), Right: (No border), Between : (No border), Tab stops: 3.25", Centered + 6.5", Right

Formatted: Font color: Black

Formatted: Normal, Border: Top: (No border), Bottom: (No border), Left: (No border), Right: (No border), Between : (No border), Tab stops: 3.25", Centered + 6.5", Right

rural) households from 305 clusters were selected. The whole procedure of sampling is found in the full 2019 EMDHS report (23).

Study variables

The response variable was under-five mortality status, which was categorized as (child alive: Yes= 0 and No = 1). The age was recorded in months. Independent variables were classified as individual level and community level predictors (Place of residence, Region, Community place of delivery, Community wealth, Community media exposure, and Community toilet facility~~).~~), some of this community-level predictors are driven from individual-level predictors. The individual-level predictors were further categorized as socio-demographic and economic factors (Educational level of the mother, Sex of household head, age of household head, Number of household members, Number of a child under the age of five, Marital status of the mother~~, Wealth index~~, Source of water, Time to get water, Type of toilet facility, Household electricity, Types of cooking fuel, Main floor material, Main wall material, and Main roof material~~, and Media exposure~~) and Maternal and child factors (Maternal age at first birth, Sex of the child, Utilization of contraception, Order of birth, Mode of delivery, duration of breastfeeding and Multiple births~~).~~) **(Figure 1)**.

Spatial analysis

The data for spatial analysis was cleaned and merged by using STATA version 16 and excel soft wares. ArcGIS version 10.8 and saTScan version 9.7 were used for the spatial analysis.

Spatial autocorrelation

Spatial autocorrelation (Global Moran's I) was conducted to see if under-five mortality was measured as dispersed (Moran's I value closer to -1), clustered (Moran's I value closer to 1), or randomly distributed (Moran's I value of 0) in Ethiopia~~.~~ (25).

Formatted: Font color: Black

Formatted: Normal, Border: Top: (No border), Bottom: (No border), Left: (No border), Right: (No border), Between : (No border), Tab stops: 3.25", Centered + 6.5", Right

Formatted: Font color: Black

Formatted: Normal, Border: Top: (No border), Bottom: (No border), Left: (No border), Right: (No border), Between : (No border), Tab stops: 3.25", Centered + 6.5", Right

Spatial interpolation

Though all enumeration areas have known under-five mortality, areas that are not selected were predicted. Spatial interpolation was applied by using the geostatistical ordinary Kriging spatial interpolation technique to predict under-five mortality from a limited number of sample data points (EAs) to un-sampled areas. (26).

Spatial scan statistics

Sat scanSaTscan was used based on the Bernoulli test for cases (child is not alive) and controls (child is alive) to conduct the scan analysis. The upper limit used was the default maximum spatial cluster size of less than 50% of the population, allowing both small and large clusters to be detected, and clusters that contained more than the maximum limit with the circular shape of the window were avoided. (27). Most likely clusters were identified using p-values and likelihood ratio tests, which is the ratio of the likelihood of the alternative hypothesis (higher activity level inside the window) over the likelihood of the null hypothesis (same activity level inside and outside).

Data management and analysis

We used STATA version 16 and R statistical software version 4.0.5 to analyze the data. Data (KR) was cleaned and 31 variables were kept that were needed for the analysis. Residents who are not De jure (included in the study if they lasted the night before the study) were excluded which affect under-five mortality, as they couldn't respond to most of the socio-demographic and economic characteristics though they can answer the maternal and child characteristics, which has changed our sample size from 5753 to 5695.

Formatted: Font color: Black

Formatted: Normal, Border: Top: (No border), Bottom: (No border), Left: (No border), Right: (No border), Between : (No border), Tab stops: 3.25", Centered + 6.5", Right

Formatted: Font color: Black

Formatted: Normal, Border: Top: (No border), Bottom: (No border), Left: (No border), Right: (No border), Between : (No border), Tab stops: 3.25", Centered + 6.5", Right

The outcome variable was re-coded to (child alive: Yes= 1 and No = 0). ~~Generating of other~~The variables~~,~~ were further recording, renaming, ~~and~~ labeling, and generating new variables from the original variables was done. ~~New 4~~These additional four community variables were generated by taking the individual variable, calculating their proportion, and dichotomizing them based on their mean or median according to their distribution and merging variables.

In the end, we had 29 predictor variables out of which 6 were community-level predictor variables and 23 individual-level predictor variables.

As we have a binary outcome variable (child alive = yes/no), to analyze factors associated with under-five mortality, binary logistic regression is appropriate. But based on EMDHS; respondents with the same cluster show similar findings or ~~function~~functions at the same level and the data has a hierarchical structure. This makes binary logistic regression not to be used as it violates the assumption of independence of the residuals; rather a model which considers clustering effect should be used (8)(10). Multilevel logistic modeling separates the within-cluster effects from the between-cluster effects (24). Therefore, to assess the predictors associated with U5M, ~~the~~a non-weighted multilevel logistic regression model was used.

Bivariable multilevel logistic regression was used for each predictor variable to screen out the significant variables (p-value less than 0.2) for multivariable multilevel logistic models. Twenty predictor variables (3 of them were community-level predictor variables) passed to multivariable analysis. In multivariable analysis, a p-value less than 0.05 was considered a factor associated with U5M.

The first model fitted was the null model (intercept model) which contains the outcome variable only (under-five mortality status) with the cluster number. The intra-cluster correlation (ICC) was used to assess whether there was a random effect. ICC of 0.130 which ~~is not negligible that~~ means there is a 13% under-five mortality which is explained by between cluster difference and around 87% of the under-five mortality is explained by within-cluster difference~~.~~, which was not negligible. The second model was fitted by using the outcome, the cluster and as a predictor variable the individual level only. The probability of U5M was predicted as a function of individual-level predictors.

Formatted: Font color: Black

Formatted: Normal, Border: Top: (No border), Bottom: (No border), Left: (No border), Right: (No border), Between : (No border), Tab stops: 3.25", Centered + 6.5", Right

Formatted: Tab stops: Not at 2.32"

Formatted: Font color: Black

Formatted: Normal, Border: Top: (No border), Bottom: (No border), Left: (No border), Right: (No border), Between : (No border), Tab stops: 3.25", Centered + 6.5", Right

For the third model the outcome variable, the cluster number, and the community level predictor variables were accounted for. Then the final model was fitted with both the individual level and the community level predictor variables taken into account.

The models were compared by using a log-likelihood statistic, where the fitted model had the smallest -2log * likelihood value that is deviance.

Model performance was checked by using residual diagnostic plot in R software package DHARMa.

Ethical consideration

Permission for data access was obtained from a major demographic and health survey through an online request from http://www.dhsprogram.com. The data used for this study were publicly available with no personal identifier.

Formatted: Font color: Black

Formatted: Normal, Border: Top: (No border), Bottom: (No border), Left: (No border), Right: (No border), Between : (No border), Tab stops: 3.25", Centered + 6.5", Right

Formatted: Font: Times New Roman, 12 pt

Formatted: Justified

Formatted: Font color: Black

Formatted: Normal, Border: Top: (No border), Bottom: (No border), Left: (No border), Right: (No border), Between : (No border), Tab stops: 3.25", Centered + 6.5", Right

Result

~~Background socio~~Socio-demographic Background and maternal and child health factor

An overall of 5,695 live births was encompassed in the study, of which 2,942 (51.66%) were male, 5,528 (97.07%) were single children, 2,937 (51.57%) were delivered at home and around 6.04% of the children were born via cesarean section (**Table 1**)

Out of the total children's mothers, most were married 5,346 (93.87 %) and 3,135 (55.05%) have no education, only 291(5.11%) attain higher education, and Greater than 51.62% of the respondents are of poor economic status. The majority of the respondents 64.27% are utilizers of improved water and 30.99 % travel more than 30 minutes, 50.85% travel less than 30 min to get water unlike those who have water on-premises. The majority of sex of household head is male 4,561 (80.09%) and the commonest age of household head is 35-50 with 45.58%.

Background of community factors (**Table 1**)

Among the population, the majority of the participants were rural residents with 4389(77.07%). And 3457 (60.70%) of the community practiced home delivery. The number of communities that utilize unimproved toilets is 3175(55.75%). 3391 of the population (59.54%) are not exposed to the media. And the majority of the children reside in rural areas (**Table 2**)

Prevalence of under-five mortality

The under-five mortality is 5.9% with its 95% (CI 5.4%, 6.6%) and it varies across regions ranging from 2.13% in Addis Ababa to 9.82 in the Somali region. Out of the total 918(15.79%) of children who had a history of lower than 6 months of breastfeeding died (Table 3)

Spatial autocorrelation

The global Moran's index was 0.09, z-score 3.5, p-value <0.001 suggesting that the spatial distribution under-five mortality is not at random in the 2019 EMDHS (**Figure ~~1)~~2)**

Formatted: Font color: Black
Formatted: Normal, Border: Top: (No border), Bottom: (No border), Left: (No border), Right: (No border), Between : (No border), Tab stops: 3.25", Centered + 6.5", Right
Formatted: Font color: Auto
Formatted: Space After: 10 pt, Border: Top: (No border), Bottom: (No border), Left: (No border), Right: (No border), Between : (No border), Tab stops: Not at 6.49"
Formatted: No underline, Font color: Auto
Formatted: Font color: Auto
Formatted: Font color: Auto
Formatted: Font color: Auto
Formatted: Font color: Auto
Formatted: Space After: 10 pt, Border: Top: (No border), Bottom: (No border), Left: (No border), Right: (No border), Between : (No border), Tab stops: Not at 6.49"
Formatted: Font color: Auto
Formatted: Font color: Auto
Formatted: Font color: Auto
Formatted: Font color: Auto
Formatted: Space After: 10 pt, Border: Top: (No border), Bottom: (No border), Left: (No border), Right: (No border), Between : (No border), Tab stops: Not at 6.49"
Formatted: Font color: Auto
Formatted: Font color: Auto
Formatted: Font: Not Bold, Font color: Auto
Formatted: Font color: Auto
Formatted: Font color: Auto
Formatted: Space After: 10 pt, Border: Top: (No border), Bottom: (No border), Left: (No border), Right: (No border), Between : (No border)
Formatted: Font color: Auto
Formatted: Font color: Black
Formatted: Normal, Border: Top: (No border), Bottom: (No border), Left: (No border), Right: (No border), Between : (No border), Tab stops: 3.25", Centered + 6.5", Right

Hotspot analysis

A high proportion of U5M was observed around the regions of Somali and Dire Dawa (**Figure** ~~2)~~3))

Spatial Interpolation

The spatial interpolation or prediction analysis result showed that the under-five mortality ranges from 0% to 50%. The high risk predicted areas were located in Somali, Dire Dawa, Benishangul Gumuz, Gambella, and SNNPR (**Figure** ~~3~~4)

Spatial scan statistics analysis

As observed in the figure ~~22~~5 significant clusters were identified in the Somali region. Located within 7.384788 N, 45.908936 E with 612.75 km radius. Children living in the primary cluster were 62% more likely to die before they celebrate their fifth birthday when compared to those who lived outside the window (relative risk (1.62), Likelihood ratio (7.36), and p-value (0.03)) (**Figure** ~~4~~5)

Factors associated with Under-five mortality

By using multilevel logistic regression, after filtering all variables in bivariable multilevel logistic regression, 4 models were fitted (null model, individual-level model, community-level model, and both individual and community level). The ICC was found to be 0.13 (95% CI: (0.08, 0.2)) which is not negligible. The 4th model was the best-fitted model with the lowest deviance. When the number of household members increases by one, the odds of under-five mortality will be decreased by 8.5% (AOR=0.09, 95%CI: (0.84, 0.99)). The odds of under-five death will be decreased by 82.8% (AOR=0.17, 95%CI: 0.14, 0.21) as the number of under-five children in the family increases by one. Children who have multiple births have 14 times higher odds of under-five mortality (AOR=14.4, 95%CI: (8.5, 24.3)) than singletons. Children who breastfed for less than 6months (AOR=5.04, 95%CI: (3.81, 6.67)) have 5 times higher odds of experiencing under-five mortality than those who have breastfed. People whose main roof is palm/bamboo have 43% (AOR=0.57, 95%CI: (0.34, 0.96)) decreased odds of having under-five deaths in the family than that of corrugated iron. Under-five children who are in the sixth birth order and more are 2.5

Formatted: Font color: Black

Formatted: Normal, Border: Top: (No border), Bottom: (No border), Left: (No border), Right: (No border), Between : (No border), Tab stops: 3.25", Centered + 6.5", Right

Formatted: Font color: Auto

Formatted: Font color: Auto

Formatted: Font color: Auto

Formatted: Font color: Auto

Formatted: Space After: 10 pt, Border: Top: (No border), Bottom: (No border), Left: (No border), Right: (No border), Between : (No border), Tab stops: Not at 6.49"

Formatted: Space After: 10 pt, Border: Top: (No border), Bottom: (No border), Left: (No border), Right: (No border), Between : (No border), Tab stops: Not at 6.49"

Formatted: Font color: Black

Formatted: Normal, Border: Top: (No border), Bottom: (No border), Left: (No border), Right: (No border), Between : (No border), Tab stops: 3.25", Centered + 6.5", Right

(AOR=2.46, 95%CI: (1.49, 4.06)) times higher odds to have under-five mortality as compared with second and third birth order. Place of delivery was found to have a significant association with U5M, those who have delivered in institutions have 43% less chance of experiencing U5M (AOR=0.57, 95%CI: (0.41, 0.81)) as compared with those delivered at home. Being a resident of the Somali and Afar region increases the odds of under-five death by 3.5 (AOR=3.46, 95%CI: (1.58, 7.55)) and 2.5 (AOR=2.54, 95%CI: (1.10, 5.85)) times respectively as compared with the Tigray region (Table 4).

The residual plot shows that the model performs adequately, with the KS test being insignificant (p-value = 0.29), and also the deviation was not significant (**Figure 6**).

Discussion

The study mainly focused on the spatial distribution of under-five mortality and on factors that influence it. The findings of this study revealed that the prevalence of under-five mortality was 59 per 1000 live births with (95% CI: (54, 66)). The multivariable multilevel logistic regression model result showed that family size, number of under~~-~~-five children, birth order, ~~breast feed~~breastfeeding, multiple birth, region, and institutional delivery were associated with under-five mortality in Ethiopia. The spatial analysis result revealed that Somali ~~and~~, Dire Dawa and Eastern Oromia were high~~-~~-risk areas for under five ~~mortality~~mortalities in Ethiopia.

The study showed that under-five mortality in Ethiopia is 59 per 1000 live births with (95% CI: (5.4%, 6.6%)). This finding is still below the sustainable development goal target of 25 or low deaths per 1000 live births also it is not consistent with studies conducted in Ethiopia (8)(19), this difference might be due to the survey time of the data used and the sample size.

Increased number of households (family size) resulted in decreased odds of under-five death. It is in line with a ~~similar~~ study in Ethiopia (17), and Eastern Nigeria (~~25~~28). This can be explained by, as the family size increases, the mother of the children will be more experienced in taking good and adequate care of the children~~. As the number of household~~ and as well as there will be many

Formatted: Font color: Black
Formatted: Normal, Border: Top: (No border), Bottom: (No border), Left: (No border), Right: (No border), Between : (No border), Tab stops: 3.25", Centered + 6.5", Right
Formatted: Font color: Auto
Formatted: Font color: Auto
Formatted: Font: Not Bold, Font color: Auto
Formatted: Font color: Auto
Formatted: Font: Bold
Formatted: Font color: Auto
Formatted: Font color: Auto
Formatted: Font color: Auto
Formatted: Font color: Auto
Formatted: Font color: Auto
Formatted: Font color: Auto
Formatted: Font color: Auto
Formatted: Font color: Auto
Formatted: Font color: Auto
Formatted: Font color: Auto
Formatted: Font color: Auto
Formatted: Font color: Auto
Formatted: Font color: Auto
Formatted: Font color: Auto
Formatted: Font color: Auto
Formatted: Font color: Auto
Formatted: Font color: Auto
Formatted: Font color: Auto
Formatted: Font color: Auto
Formatted: Font color: Black
Formatted: Normal, Border: Top: (No border), Bottom: (No border), Left: (No border), Right: (No border), Between : (No border), Tab stops: 3.25", Centered + 6.5", Right

family members ~~is increasing, many people~~that can care for ~~the~~ under-five children~~ with fewer burdens.~~.

As the number of under-five children in a family ~~increases~~increase, the odds of under-five mortality will be decreased. This is ~~consistent~~in agreement with a study in ~~Bangladesh (26) and~~ Ghana (~~26~~29). This is because the mother will be more experienced and she will learn from her previous mistakes if there is one. But there is a contradiction with another study result in Ethiopia, which demonstrated that the odds of under-five mortality ~~increases~~increase as the number of under-five children increases (18). This can be justified as the number of under-five children increases~~;~~ it will be difficult to fulfill their basic needs and their family (mother) will be exhausted to look after the children.

According to our study results being fourth and fifth or sixth and above-born children ~~increases~~increase the odds of under-five mortality. This is in line with a study conducted in rural parts of Ethiopia (11). But it is not supported by a study conducted in Ethiopia which pointed out that being firstborn increases the odds of under-five mortality by two folds when compared to those with five and above birth orders (18). This can be because firstborns are more likely to be hospitalized as a result of congenital malformations and perinatal conditions in their early childhood. But, this changes for firstborn children as they get older, when younger brothers and sisters are more likely to be hospitalized for injuries and avoidable conditions, which shows less parental care (~~27).~~30).

Breastfeeding less than six months increases the odds of under-five mortality~~ by 6.78 fold as~~ compared with those who breastfed for six and more months. Which is consistent with a finding in Ethiopia (18). This can be justified by~~,~~ breastfeeding ~~is~~being one of the prevention methods for reducing child mortality and it is a means of prevention of delayed growth ~~too (28).~~(31).

Being multiple birth children increases the odds of under-five mortality ~~by fourteen times that of~~when in comparison to singleton children. This finding is in line with different studies conducted in Ethiopia at different times, a study conducted in rural parts of Ethiopia (11)(16)(7)(8)(18). This can be due to multiple ~~birth imposes~~births imposing short- and long-term medical complications (~~29).~~32).

Formatted: Font color: Black
Formatted: Normal, Border: Top: (No border), Bottom: (No border), Left: (No border), Right: (No border), Between : (No border), Tab stops: 3.25", Centered + 6.5", Right
Formatted: Font color: Auto
Formatted: Font color: Auto
Formatted: Font color: Auto
Formatted: Font color: Auto
Formatted: Font color: Auto
Formatted: Font color: Auto
Formatted: Font color: Auto
Formatted: Font color: Auto
Formatted: Font color: Auto
Formatted: Font color: Auto
Formatted: Font color: Auto
Formatted: Not Highlight
Formatted: Font color: Auto, Not Highlight
Formatted: Not Highlight
Formatted: Not Highlight
Formatted: Not Highlight
Formatted: Not Highlight
Formatted: Font color: Auto, Not Highlight
Formatted: Font color: Black
Formatted: Normal, Border: Top: (No border), Bottom: (No border), Left: (No border), Right: (No border), Between : (No border), Tab stops: 3.25", Centered + 6.5", Right

Facility or institutional delivery reduces the odds of under-five mortality by 43% as compared to home delivery. This finding is consistent with studies conducted in Ethiopia (10)(11) and the Democratic Republic of Congo (14). This is because; Home delivery increases the death of under-five children due to a higher risk of postpartum complications after the intrapartum, exposure to common vaccine-preventable diseases as a result of an absence of vaccination, and less likely to attend prenatal and postpartum visits (10)(33).

The odds of under-five mortality are higher for residents of Afar and Somali regions ~~by 2.5 and 3.5 times respectively~~ as compared to the Tigray region. Somali and Afar have the first and second lowest coverage of children with all basic vaccinations. Children under five with symptoms of Acute Respiratory Infection and diarrhea are below the national average. There is serious child malnutrition with the first and second highest prevalence of wasting for Somali and Afar respectively (~~30)(31).~~34)(35) and the Somali region was one of the regions that were in the primary cluster from our spatial scan statistics analysis where under-five mortality is more likely to occur. This is not supported by studies conducted in Ethiopia based on the 2011 and 2016 EDHS (9)(10)(11) where other regions are found to have a significant association with under-five mortality. This discrepancy can be a result of the different number of enumeration areas (clusters) which led to different samples, different statistical models used for analysis (hurdle negative binomial mode1, multilevel negative binomial regression model, and binary logistic regression).

~~Facility or institutional delivery reduces the odds of under-five mortality by 43% as compared to home delivery. This finding is consistent with studies conducted in Ethiopia (10)(11) and the Democratic Republic of Congo (14). This is because; Home delivery increases the death of under-five children due to a higher risk of postpartum complications after the intrapartum, exposure to common vaccine-preventable diseases as a result of an absence of vaccination, and less likely to attend prenatal and postpartum visits (10)(32).~~

Conclusion: Under-five mortality in Ethiopia is 59 per 1000 live births which ~~is~~are high and unacceptable as compared to the 2030 agenda for sustainable development ~~-~~aimed at 25 per 1000 live births. ~~Breast feed~~Breastfeeding more than 6~~-~~-month, twin birth, institutional delivery and high~~-~~-risk areas of U5M (Somali and dire Dawa) were modifiable risk factors. Therefore, maternal education on the advantage of ~~breast feeding~~breastfeeding and institutional delivery is highly recommended. Women who deliver ~~twin~~multiple births should be given special attention. An

Formatted: Font color: Black

Formatted: Normal, Border: Top: (No border), Bottom: (No border), Left: (No border), Right: (No border), Between : (No border), Tab stops: 3.25", Centered + 6.5", Right

Formatted: Font color: Auto, Not Highlight

Formatted: Not Highlight

Formatted: Font color: Auto, Not Highlight

Formatted: Font color: Auto, Not Highlight

Formatted: Font color: Auto, Not Highlight

Formatted: Font color: Black

Formatted: Font color: Black

Formatted: Normal, Border: Top: (No border), Bottom: (No border), Left: (No border), Right: (No border), Between : (No border), Tab stops: 3.25", Centered + 6.5", Right

effective strategy should be designed for intervention for hot spot areas of U5M that is Somali and Dire Dawa.

Strength and limitation of the study

We used EMDHS 2019 data which is more inclusive and generalizable as EAs are taken from most parts of the country. The larger sample size increases the power of the study and helps to make geographical comparisons. As it is cross-sectional it does not show a temporal relationship (chicken egg dilemma), important variables: antenatal care follow-up, postnatal care, and preceding birth interval were excluded from the study due to their highest rate of missing values.

Abbreviation

AOR: Adjusted Odds Ratio, CI: Confidence Interval, CS: Cesarean Section, EAs: Enumeration Areas, DHS: Demography and Health Survey, EMDHS: Ethiopia Mini Demography and Health Survey, HHs: households, ICC: Intraclass Correlation, KR: kids record, SGD: Sustainable Development Goal, U5M: under-five mortality, U5MR: under-five mortality rate

Declaration

Ethics approval and consent to participate

Ethical clearance was obtained from measure DHS through filling requesting a form for accessing data. The data used in this study are publicly available, aggregated secondary data that hasn't any personal identifying information that can be linked to study participants. The confidentiality of data was maintained anonymously.

Availability of data and materials

The datasets used during the current study are publicly available data from measure DHS program

Funding

We didn't receive external funds for this research.

Formatted: Font color: Black

Formatted: Normal, Border: Top: (No border), Bottom: (No border), Left: (No border), Right: (No border), Between : (No border), Tab stops: 3.25", Centered + 6.5", Right

Formatted: Font: Times New Roman

Formatted: Font color: Black

Formatted: Normal, Border: Top: (No border), Bottom: (No border), Left: (No border), Right: (No border), Between : (No border), Tab stops: 3.25", Centered + 6.5", Right

Acknowledgment

We acknowledge the DHS program for providing us with the dataset.

Consent for publication

Not applicable as there ~~is~~are no image or other confidentiality-related issues.

Competing interests

The authors declared that they have no competing interests.

Authors' contributions

LGG, TMT, and ZTT conceived the study, data analysis, drafted the manuscript, and critically reviewed the manuscript. Read and approve the final version of the manuscript.

Formatted: Font color: Black

Formatted: Normal, Border: Top: (No border), Bottom: (No border), Left: (No border), Right: (No border), Between : (No border), Tab stops: 3.25", Centered + 6.5", Right

Formatted: Font: Times New Roman

Formatted: Font: Times New Roman

Formatted: Font color: Black

Formatted: Normal, Border: Top: (No border), Bottom: (No border), Left: (No border), Right: (No border), Between : (No border), Tab stops: 3.25", Centered + 6.5", Right

References

1. Croft, Trevor N., Aileen M. J. Marshall, Courtney K. Allen, et al. Guide to DHS Statistics. Rockville, Maryland, USA: ICF Guide. 2018;22–51.
2. World Health Organization. Proposed working definition of an older person in Africa for the MDS Project. Zagreb International Review of Economics & Business. 2018;18(1):1–15.
3. Woldeamanuel BT, Aga MA. Count Models Analysis of Factors Associated with Under-Five Mortality in Ethiopia. Global Pediatric Health. 2021 Jan;8:2333794X2198953.
4. UN Inter-agency Group for Child Mortality. Levels and Trends in Child Mortality: Report 2020: Estimates Developed by the Un Inter-Agency Group for Child Mortality Estimation. Report 2020; 2020.
5. Johnston R. Arsenic and the 2030 Agenda for Sustainable Development. In: Arsenic in the Environment - Proceedings [Internet]. CRC Press; 2016 [cited 2021 Jun 20]. p. 12–4. Available from: http://dx.doi.org/10.1201/b20466-7
6. Li Z, Hsiao Y, Godwin J, Martin BD, Wakefield J, Clark SJ. Changes in the spatial distribution of the under-five mortality rate: Small-area analysis of 122 DHS surveys in 262 subregions of 35 countries in Africa. PLOS ONE. 2019 Jan 22;14(1):e0210645.
7. Fenta SM, Fenta HM, Ayenew GM. The best statistical model to estimate predictors of under-five mortality in Ethiopia. Journal of Big Data. 2020 Aug 17;7(1).
8. Worku MG, Teshale AB, Tesema GA. Determinants of under-five mortality in the high mortality regions of Ethiopia: mixed-effect logistic regression analysis. Archives of Public Health. 2021 Apr 23;79(1).
9. Ayele DG, Zewotir TT, Mwambi HG. Structured additive regression models with spatial correlation to estimate under-five mortality risk factors in Ethiopia. BMC Public Health. 2015 Mar 19;15(1).
10. Geremew BM, Gelaye KA, Melesse AW, Akalu TY, Baraki AG. Factors Affecting Under-Five Mortality in Ethiopia: A Multilevel Negative Binomial Model. Pediatric Health, Medicine and Therapeutics. 2020 Dec;Volume 11:525–34.
11. Gebremichael SG, Fenta SM. Under-Five Mortality and Associated Risk Factors in Rural Settings of Ethiopia: Evidence from 2016 Ethiopian Demographic and Health Survey. Advances in Public Health. 2020 Aug 31;2020:1–13.

Formatted: Font color: Black

Formatted: Normal, Border: Top: (No border), Bottom: (No border), Left: (No border), Right: (No border), Between : (No border), Tab stops: 3.25", Centered + 6.5", Right

Formatted: Font color: Black

Formatted: Normal, Border: Top: (No border), Bottom: (No border), Left: (No border), Right: (No border), Between : (No border), Tab stops: 3.25", Centered + 6.5", Right

Formatted: Font color: Black

Formatted: Normal, Border: Top: (No border), Bottom: (No border), Left: (No border), Right: (No border), Between : (No border), Tab stops: 3.25", Centered + 6.5", Right

12. Singh A, Masquelier B. Continuities and changes in spatial patterns of under-five mortality at the district level in India (1991–2011). International Journal of Health Geographics. 2018 Nov 15;17(1).

13. Ghilagaber G, Antai D, Kandala N-B. Modeling Spatial Effects on Childhood Mortality Via Geo-additive Bayesian Discrete-Time Survival Model: A Case Study from Nigeria. In: Advanced Techniques for Modelling Maternal and Child Health in Africa [Internet]. Dordrecht: Springer Netherlands; 2013 [cited 2021 Jun 20]. p. 29–48. Available from: http://dx.doi.org/10.1007/978-94-007-6778-2_3

14. Kandala N-B, Mandungu TP, Mbela K, Nzita KP, Kalambayi BB, Kayembe KP, et al. Child mortality in the Democratic Republic of Congo: cross-sectional evidence of the effect of geographic location and prolonged conflict from a national household survey. BMC Public Health. 2014 Mar 20;14(1).

15. Anafcheh T, Yaghoubi Doust M, Mojadam M, Mirkazemi R, Khafaie MA. Temporal and spatial distribution of under-five mortality and factors associated with multiple cases of under-five deaths within a family in the rural area of Khuzestan, Southern Iran. Scientific Reports. 2018 Dec;8(1).

16. Dejene T, Girma E. Social determinants of under-five mortality in Ethiopia: Event history analysis using evidence from Ethiopian Demographic and Health Survey (EDHS). Health. 2013;05(05):879–84.

17. Ayele DG, Zewotir TT. Childhood mortality spatial distribution in Ethiopia. Journal of Applied Statistics. 2016 Feb 18;43(15):2813–28.

18. Zewudie AT, Gelagay AA, Enyew EF. Determinants of Under-Five Child Mortality in Ethiopia: Analysis Using Ethiopian Demographic Health Survey, 2016. International Journal of Pediatrics. 2020 Sep 22;2020:1–9.

19. Fikru C, Getinet M, Shaweno T. Proximate Determinants of Under-Five Mortality in Ethiopia: Using 2016 Nationwide Survey Data. Pediatric Health, Medicine and Therapeutics. 2019 Dec;Volume 10:169–76.

20. Abir T, Agho KE, Page AN, Milton AH, Dibley MJ. Risk factors for under-5 mortality: evidence from Bangladesh Demographic and Health Survey, 2004–2011. BMJ Open. 2015 Aug;5(8):e006722.

Formatted: Font color: Black

Formatted: Normal, Border: Top: (No border), Bottom: (No border), Left: (No border), Right: (No border), Between : (No border), Tab stops: 3.25", Centered + 6.5", Right

21. Pretorius CE, Asare H, Genuneit J, Kruger HS, Ricci C. Impact of breastfeeding on mortality in sub-Saharan Africa: a systematic review, meta-analysis, and cost-evaluation. European Journal of Pediatrics. 2020 Jun 26;179(8):1213–25.

22. Country profile: Croatia. 2019 Jun 18;

23. Ethiopian Public Health Institute (EPHI) [Ethiopia] and ICF. Ethiopia Mini Demographic and Health Survey 2019: Final Report. Rockville, Maryland, USA: EPHI and ICF; 2021.

24. Sommet N, Morselli D. Keep Calm and Learn Multilevel Logistic Modeling: A Simplified Three-Step Procedure Using Stata, R, Mplus, and SPSS. International Review of Social Psychology. 2017 Sep 8;30(1):203–18.

25. Fortin MJ, Dale M. Spatial autocorrelation. In: Fotheringham AS, Rogerson PA. eds. The SAGE Handbook of Spatial Analysis. Thousand Oaks, CA: SAGE; 2009;89–101.

26. Bhunia GS, Shit PK, Maiti R. Comparison of GIS-based interpolation methods for spatial distribution of soil organic carbon (soc). Journal of the Saudi Society of Agricultural Sciences 2018;17:114–26. 10.1016/j.jssas.2016.02.001

27. Kulldorff M. Bernoulli, Discrete Poisson and Continuous Poisson Models: A spatial scan statistic. Communications in Statistics: Theory and Methods, 1997; 26:1481-1496.

28. Azuike EC. Determinants of Under-Five Mortality in South-Eastern Nigeria. Community Medicine and Public Health Care. 2019 Jul 10;6(2):1–6.

~~26~~29. Aheto JMK. Predictive model and determinants of under-five child mortality: evidence from the 2014 Ghana demographic and health survey. BMC Public Health. 2019 Jan 14;19(1).

~~27~~30. Björkegren E, Svaleryd H. Birth Order and Child Health . Institute for Evaluation of Labour Market and Education Policy (IFAU). 2017(16).

~~28~~31. WHO UNICEF IBFAN. (2016). Marketing of Substitutes : National Implementation of the International Code Status Report. 2016.

~~29~~32. Elster N. Less is more: the risks of multiple births. Fertility and Sterility. 2000 Oct;74(4):617–23.

~~30~~33.~~. UNICEF Ethiopia. Regional Situation Analysis of Children and Women: Afar Region . 2019 Dec.~~

~~31. UNICEF Ethiopia. Regional Situation Analysis of Children and Women: Somali Region. 2019 Dec.~~

Formatted: Font color: Black

Formatted: Normal, Border: Top: (No border), Bottom: (No border), Left: (No border), Right: (No border), Between : (No border), Tab stops: 3.25", Centered + 6.5", Right

Formatted: Font: Arial, 10 pt, Font color: Custom Color(RGB(48,48,48)), Pattern: Clear (White)

Formatted: Font color: Black

Formatted: Normal, Border: Top: (No border), Bottom: (No border), Left: (No border), Right: (No border), Between : (No border), Tab stops: 3.25", Centered + 6.5", Right

~~32.~~ Goyal S, Kortsmit K, Cox S, D'Angelo DV, Romero L, Henderson ZT, et al. Prevalence of Home Births and Associated Risk Profile and Maternal Characteristics, 2016–2018. Obstetrics & Gynecology. 2020 Nov 4;136(6):1195–203.

34. UNICEF Ethiopia. Regional Situation Analysis of Children and Women: Afar Region . 2019 Dec.

35. UNICEF Ethiopia. Regional Situation Analysis of Children and Women: Somali Region. 2019 Dec.

List of Figures

Figure 1 conceptual framework of under-five mortality in Ethiopia 2019.

Figure 2 Spatial autocorrelation of under-five mortality in Ethiopia 2019.

Figure ~~2~~3 Hotspot analysis of under-five mortality in Ethiopia 2019.

Figure ~~3~~4 Empirical Bayesian Kriging prediction map of under-five mortality in Ethiopia 2019.

Figure ~~4~~5 Spatial scan statistics of under-five mortality in Ethiopia 2019.

Figure 6 residual diagnostic plot for the final model of under-five mortality in Ethiopia 2019.

Table 1 Sociodemographic, maternal, and child health factors

Sociodemographic, Maternal and child health factors		Frequency	Percentage
Age of the mother at first birth	Less than 18	3,024	53.10%
	19 upto 34	2,650	46.53%
	35 and more	21	0.37%

Formatted: Font color: Black

Formatted: Normal, Border: Top: (No border), Bottom: (No border), Left: (No border), Right: (No border), Between : (No border), Tab stops: 3.25", Centered + 6.5", Right

Formatted Table

Formatted: Font color: Black

Formatted: Normal, Border: Top: (No border), Bottom: (No border), Left: (No border), Right: (No border), Between : (No border), Tab stops: 3.25", Centered + 6.5", Right

Sex of child	Male	2,942	51.66%
	Female	2,753	48.34%
Contraceptive utilization	not use	3,875	68.04%
	use	1,820	31.96%
Multiple births	Singleton	5,528	97.07%
	Multiple	167	2.93%
Birth order	1st order	1,228	21.56%
	2nd and 3rd order	1,883	33.06%
	4th and 5th order	1,298	22.79%
	6th order	1,286	22.58%
Place where the mother delivered	home delivery	2,937	51.57%
	Facility delivery	2,758	48.43%
Delivery by cesarean section	No	5,351	93.96%
	yes	344	6.04%
Months of Breastfeed	Less than 6month	918	16.12%
	6 month and grater	4,777	83.88%
Sex of household head	Male	4,561	80.09%
	Female	1,134	19.91%
Age of household head	less than 34	2,479	43.53%
	35 ~~upto~~up to 50	2,596	45.58%
	50 and more	620	10.89%
Marital Status	Married	5,346	93.87%
	Unmarried	349	6.13%
Highest educational level	No education	3,135	55.05%
	Primary	1,802	31.64%
	Secondary	467	8.20%
	Higher	291	5.11%
Wealth index	Poor	2,940	51.62%
	Middle	797	13.99%
	Rich	1,958	34.38%

Formatted: Font color: Black

Formatted: Normal, Border: Top: (No border), Bottom: (No border), Left: (No border), Right: (No border), Between : (No border), Tab stops: 3.25", Centered + 6.5", Right

Formatted: Font color: Black

Formatted: Normal, Border: Top: (No border), Bottom: (No border), Left: (No border), Right: (No border), Between : (No border), Tab stops: 3.25", Centered + 6.5", Right

Water source	Improved	3,660	64.27%
	Unimproved	2,035	35.73%
Time to fetch water	more than 30 min	1,765	30.99%
	less than 30 min	2,896	50.85%
	On premises	1,034	18.16%
Type of latrine used	Improved	1,164	20.44%
	Not improved	4,531	79.56%
Household has electricity	No	4,073	71.52%
	Yes	1,622	28.48%
Cooking fuel in use	Clean fuel	432	7.59%
	Solid fuel	5,263	92.41%
Type of floor of the house	Earth/sand/dung	4,384	76.98%
	Wood	49	0.86%
	Cement	799	14.03%
	Carpet	463	8.13 %
Type of the house wall	Cane/Palm/Trunk/Reed	1,261	22.14%
	Bamboo/wood	3,531	62.00%
	Cement/stone with lime/bricks/covered adobe	903	15.86%
Type of the house roof	Thatch	1,488	26.13%
	Palm/bam	995	17.47%
	Cement	198	3.48%
	corrugated iron	3,014	52.92 %

Table 2 Community-level factors

Community level factors		frequency	Percentage
Place of residence	Urban	1,306	22.93%

Formatted: Font color: Black

Formatted: Normal, Border: Top: (No border), Bottom: (No border), Left: (No border), Right: (No border), Between : (No border), Tab stops: 3.25", Centered + 6.5", Right

Formatted Table

Formatted: Font color: Black

Formatted: Normal, Border: Top: (No border), Bottom: (No border), Left: (No border), Right: (No border), Between : (No border), Tab stops: 3.25", Centered + 6.5", Right

	Rural	4,389	77.07%
Community place of delivery	Home delivery	3,457	60.70%
	Facility delivery	2,238	39.30%
Community toilet utilization	Improved	2,520	44.25%
	Not improved	3,175	55.75%
Community wealth status	Poor	2,776	48.74%
	Rich	2,919	51.26%
Community media exposure	Not exposed to media	3,391	59.54%
	Exposed to media	2,304	40.46%

Table 3 Under-five mortality among predictors

Characteristics		Is the child alive?		total
		Yes 5357(94.06%)	No 338(5.94%)	
Is child twin?	Yes	123	44	167
	No	5234	294	5,528
Region	Tigray	436	14	450
	Afar	616	35	651
	Amhara	478	23	501
	Oromia	674	41	715
	Somali	580	57	637
	Benishangul	477	45	522
	SNNPR	631	26	657

Formatted: Font color: Black

Formatted: Normal, Border: Top: (No border), Bottom: (No border), Left: (No border), Right: (No border), Between : (No border), Tab stops: 3.25", Centered + 6.5", Right

Formatted Table

Formatted: Font color: Black

Formatted: Normal, Border: Top: (No border), Bottom: (No border), Left: (No border), Right: (No border), Between : (No border), Tab stops: 3.25", Centered + 6.5", Right

	Gambella	409	35	444
	Harari	408	29	437
	Addis Ababa	281	6	287
	Dire Dawa	367	27	394
Breastfeeding	Less than 6 months	773	145	918
	6 months and more	4,584	193	4,777
Birth order	1st order	1,142	86	1,228
	2-3rd order	1,793	90	1,883
	4-5th order	1,231	67	1,298
	6th + order	1,191	95	1,286
Place of delivery	Home delivery	2,733	204	2,937
	Facility delivery	2,624	134	2,758

Table 4 Multivariable multilevel logistic regression analysis of both individual and community-level factors associated with under-five mortality in Ethiopia, EMDHS 2019.

	Null model	Model2	Model3	Model4
~~Variable~~	~~Null model~~	~~Model2~~ AOR(95%CI) ~~AOR(95%CI)~~	~~Model3~~ AOR(95%CI) ~~AOR(95%CI)~~	~~Model4~~ AOR(95%CI) ~~AOR(95%CI)~~
~~Education~~				
~~No education~~		~~1~~		~~1~~
~~Primary~~		~~1.14 (0.83,1.56)~~		~~1.14 (0.83,1.58)~~
~~Secondary~~		~~0. .59 (0.31, 1.13)~~		~~0.58(0.30,1.12)~~
~~Higher~~		~~0. .65(0. .28, 1.55)~~		~~0.60(0.25,1.44)~~

Formatted: Font color: Black

Formatted: Normal, Border: Top: (No border), Bottom: (No border), Left: (No border), Right: (No border), Between : (No border), Tab stops: 3.25", Centered + 6.5", Right

Inserted Cells

Formatted Table

Inserted Cells

Inserted Cells

Inserted Cells

Formatted: Font color: Black

Formatted: Normal, Border: Top: (No border), Bottom: (No border), Left: (No border), Right: (No border), Between : (No border), Tab stops: 3.25", Centered + 6.5", Right

Household members		0.922(0. 85, 1.01)		0.92(0.83,0.99) *
Number of under-five children		0.17 (0.14, 0 .22)		0.17(0.14, 0.21) ***
Sex				
Male		1		1
female		0 .83(0 .64, 1.07)		0.84(0.64,1.09)
Mode of delivery by C/S				
No		1		1
Yes		1.36 (0. 79, 2.35)		1.37(0.79,2.39)
Preceding birth order				
1st		1		1
2-3rd		1.32(0.92, 1.91)		1.34(0.93,1.94)
4-5th		1.52(0.97, 2.39)		1.51(0.96,2.38)
6+		2.44(1.48, 4.02)		2.46(1.49,4.06) ***
Time to water				
>30min		1		1
<30min		0.77(0.56, 1.04)		0.89(0.64,1.23)
On-premises		0.66(0.38, 1.14)		0.76(0.43,1.34)
Cooking fuel				
Clean fuel		1		1
Solid fuel		1.27(0.62, 2.58)		0.93(0.45,1.94)
Age at 1st birth				
19-34		1		1
<=18		0.91(0.70, 1.20)		0.90(0.68,1.18)

Formatted: Font color: Black

Formatted: Normal, Border: Top: (No border), Bottom: (No border), Left: (No border), Right: (No border), Between : (No border), Tab stops: 3.25", Centered + 6.5", Right

Formatted: Font color: Black

Formatted: Normal, Border: Top: (No border), Bottom: (No border), Left: (No border), Right: (No border), Between : (No border), Tab stops: 3.25", Centered + 6.5", Right

>=35		2.58(0.55, 12.01)		2.72(0.55,13.59)
Breastfeeding				
< 6 months		1		1
>= 6 months		5.09(3.85, 6.72)		5.05(3.82,6.67) ***
Place of delivery				
Home delivery		1		1
Facility delivery		0.54 (0.40,0.75)		0.57(0.41,0.81) **
Main floor				
earth/sand/dung		1		1
Wood		1.71(0.53, 5.52)		2.14(0.66,6.95)
Cement		0.89(0.50, 1.59)		0.86(0.48,1.58)
Carpet		1.56(0.85, 2.86)		1.28(0.67,2.46)
Main wall				
bamboo/wood		1		1
cane/palm/trunks/reed		1.42(0 .97, 2.07)		1.06(0.70,1.58)
cement/stone		1.53(0.89,2.61)		1.34(0.76,2.34)
Main roof				
Corrugated iron		1		
thatch		1.25(0.87,1.79)		1.14(0.77,1.67)
palm/bamboo		0.71(0.44,1.15)		0.57(0.34,0.96)*
calamine/cement		1.14(0.54,2.44)		1.04(0.47,2.29)
Contraceptive utilization				
No		1		1
Yes		0.72(0.53,0.98)		0.82(0.60,1.13)
Marital status				
Currently Married		1		1

Formatted: Font color: Black

Formatted: Normal, Border: Top: (No border), Bottom: (No border), Left: (No border), Right: (No border), Between : (No border), Tab stops: 3.25", Centered + 6.5", Right

Formatted: Font color: Black

Formatted: Normal, Border: Top: (No border), Bottom: (No border), Left: (No border), Right: (No border), Between : (No border), Tab stops: 3.25", Centered + 6.5", Right

Currently Unmarried		1.19(0.74,1.92)		1.22(0.75,1.97)
Multiple Twin child				
No		1		1
Yes		15.63(9.33,26.20)		14.39(8.51,24.33)***
Region				
Tigray			1	1
Afar			1.60(0.76,3.41)	2.55(1.10,5.85)*
Amhara			1.47(0.68,3.19)	0.97(0.44,2.15)
Oromia			1.73(0.84,3.55)	1.52(0.73,3.15)
Somali			2.86(1.39,5.89)	3.46(1.59,7.55)**
Benishangul gumuz			2.74(1.32,5.68)	1.95(0.91,4.20)
SNNPR			1.17(.55,2.47)	0.94(0.44,2.03)
Gambella			2.71(1.29,5.69)	1.94(0.90,4.21)
Harari			2.37(1.12,5.04)	1.95(0.87,4.36)
Addis Ababa			0.71(0.25,2.03)	0.64(0.21,2.02)
Dire Dawa			2.18(1.01,4.70)	2.02(0.91,4.50)
Community delivery place				

Formatted: Font color: Black

Formatted: Normal, Border: Top: (No border), Bottom: (No border), Left: (No border), Right: (No border), Between : (No border), Tab stops: 3.25", Centered + 6.5", Right

Formatted: Font color: Black

Formatted: Normal, Border: Top: (No border), Bottom: (No border), Left: (No border), Right: (No border), Between : (No border), Tab stops: 3.25", Centered + 6.5", Right

Home delivery			1	1
Facility delivery			0.75(0.52, 1.09)	0.94(0.62,1.43)
Community media				
Not exposed			1	1
exposed			1.09(0.75,1.58)	0.94(0.64,1.40)
Intercept	0.49(0.30,0.81)	0.22(0.08,0.63)	0.35(0.19,0.64)	0.16(0.04,0.57)
Log likelihood	-1263.56	-918.15	-1247.40	-903.72
Deviance	2527.12	1836.30	2494.80	1807.44
Variable				
Education				
No education		1		1
Primary		1.14 (0.83,1.56)		1.14 (0.83,1.58)
Secondary		0. .59 (0.31, 1.13)		0.58(0.30,1.12)
Higher		0. .65(0. .28, 1.55)		0.60(0.25,1.44)
Household members		0.922(0. 85, 1.01)		0.92(0.83,0.99) *
Number of under-five children		0.17 (0.14, 0 .22)		0.17(0.14, 0.21) ***
Sex				
Male		1		1
female		0 .83(0 .64, 1.07)		0.84(0.64,1.09)
Mode of delivery by C/S				
No		1		1

Formatted: Font color: Black

Formatted: Normal, Border: Top: (No border), Bottom: (No border), Left: (No border), Right: (No border), Between : (No border), Tab stops: 3.25", Centered + 6.5", Right

Formatted: Line spacing: Multiple 1.08 li

Formatted: Font color: Black

Formatted: Normal, Border: Top: (No border), Bottom: (No border), Left: (No border), Right: (No border), Between : (No border), Tab stops: 3.25", Centered + 6.5", Right

Yes		1.36 (0. 79, 2.35)		1.37(0.79,2.39)
Preceding birth order				
1st		1		1
2-3rd		1.32(0.92, 1.91)		1.34(0.93,1.94)
4-5th		1.52(0.97, 2.39)		1.51(0.96,2.38)
6+		2.44(1.48, 4.02)		2.46(1.49,4.06) ***
Time to water				
>30min		1		1
<30min		0.77(0.56, 1.04)		0.89(0.64,1.23)
On-premises		0.66(0.38, 1.14)		0.76(0.43,1.34)
Cooking fuel				
Clean fuel		1		1
Solid fuel		1.27(0.62, 2.58)		0.93(0.45,1.94)
Age at 1st birth				
19-34		1		1
<=18		0.91(0.70, 1.20)		0.90(0.68,1.18)
>=35		2.58(0.55, 12.01)		2.72(0.55,13.59)
Breastfeeding				
< 6 months		1		1
>= 6 months		5.09(3.85, 6.72)		5.05(3.82,6.67) ***
Place of delivery				
Home delivery		1		1
Facility delivery		0.54 (0.40,0.75)		0.57(0.41,0.81) **
Main floor				

Formatted: Font color: Black

Formatted: Normal, Border: Top: (No border), Bottom: (No border), Left: (No border), Right: (No border), Between : (No border), Tab stops: 3.25", Centered + 6.5", Right

Formatted: Font color: Black

Formatted: Normal, Border: Top: (No border), Bottom: (No border), Left: (No border), Right: (No border), Between : (No border), Tab stops: 3.25", Centered + 6.5", Right

earth/sand/dung		1		1
Wood		1.71(0.53, 5.52)		2.14(0.66,6.95)
Cement		0.89(0.50, 1.59)		0.86(0.48,1.58)
Carpet		1.56(0.85, 2.86)		1.28(0.67,2.46)
Main wall				
bamboo/wood		1		1
cane/palm/trunks/reed		1.42(0 .97, 2.07)		1.06(0.70,1.58)
cement/stone		1.53(0.89,2.61)		1.34(0.76,2.34)
Main roof				
Corrugated iron		1		
thatch		1.25(0.87,1.79)		1.14(0.77,1.67)
palm/bamboo		0.71(0.44,1.15)		0.57(0.34,0.96)*
calamine/cement		1.14(0.54,2.44)		1.04(0.47,2.29)
Contraceptive utilization				
No		1		1
Yes		0.72(0.53,0.98)		0.82(0.60,1.13)
Marital status				
Currently Married		1		1
Currently Unmarried		1.19(0.74,1.92)		1.22(0.75,1.97)
Multiple Twin child				
No		1		1
Yes		15.63(9.33,26.20)		14.39(8.51,24.33)***
Region				
Tigray			1	1

Formatted: Font color: Black

Formatted: Normal, Border: Top: (No border), Bottom: (No border), Left: (No border), Right: (No border), Between : (No border), Tab stops: 3.25", Centered + 6.5", Right

Formatted: Font color: Black

Formatted: Normal, Border: Top: (No border), Bottom: (No border), Left: (No border), Right: (No border), Between : (No border), Tab stops: 3.25", Centered + 6.5", Right

Afar			1.60(0.76,3.41)	2.55(1.10,5.85)*
Amhara			1.47(0.68,3.19)	0.97(0.44,2.15)
Oromia			1.73(0.84,3.55)	1.52(0.73,3.15)
Somali			2.86(1.39,5.89)	3.46(1.59,7.55)**
Benishangul gumuz			2.74(1.32,5.68)	1.95(0.91,4.20)
SNNPR			1.17(.55,2.47)	0.94(0.44,2.03)
Gambella			2.71(1.29,5.69)	1.94(0.90,4.21)
Harari			2.37(1.12,5.04)	1.95(0.87,4.36)
Addis Ababa			0.71(0.25,2.03)	0.64(0.21,2.02)
Dire Dawa			2.18(1.01,4.70)	2.02(0.91,4.50)
Community delivery place				
Home delivery			1	1
Facility delivery			0.75(0.52, 1.09)	0.94(0.62,1.43)
Community media				
Not exposed			1	1
exposed			1.09(0.75,1.58)	0.94(0.64,1.40)

Formatted: Font color: Black

Formatted: Normal, Border: Top: (No border), Bottom: (No border), Left: (No border), Right: (No border), Between : (No border), Tab stops: 3.25", Centered + 6.5", Right

Formatted: Font color: Black

Formatted: Normal, Border: Top: (No border), Bottom: (No border), Left: (No border), Right: (No border), Between : (No border), Tab stops: 3.25", Centered + 6.5", Right

Intercept	0.49(0.30,0.81)	0.22(0.08,0.63)	0.35(0.19,0.64)	0.16(0.04,0.57)
ICC	0.130	0.036	0.091	0.025
Log-likelihood	-1263.56	-918.15	-1247.40	-903.72

*** P-value<0.001, ** p-value<0.01, * p-value<0.05

Formatted: Font color: Black

Formatted: Normal, Border: Top: (No border), Bottom: (No border), Left: (No border), Right: (No border), Between : (No border), Tab stops: 3.25", Centered + 6.5", Right

Formatted: Justified, Widow/Orphan control, Border: Top: (No border), Bottom: (No border), Left: (No border), Right: (No border), Between : (No border)

Formatted: Font color: Black

Formatted: Normal, Border: Top: (No border), Bottom: (No border), Left: (No border), Right: (No border), Between : (No border), Tab stops: 3.25", Centered + 6.5", Right

PLOS ONE

Point by point response for editors/reviewers' comments

The manuscript title "Geographic variation and factors associated with under-five mortality in 2 Ethiopia. A spatial and multilevel analysis of Ethiopian Mini Demographic and 3 Health Surveys 2019"

Manuscript number: PONE-D-21-20589

Dear editor/reviewer,

I would like to thank you for these constructive, building, and improvable comments on this manuscript that would improve the substance and content of the manuscript. We considered each comment and clarification question of reviewers on the manuscript thoroughly. My point-by-point responses for each comment and question are described in detail on the following pages. Further, the details of changes were shown by track changes in the supplementary document attached.

Reviewer comment and authors response

Reviewer #1:

General comments:

Reviewer's comment: - Overall authors have explained regional variation in under five mortality and the key predictors of U5M in Ethiopia. I have few comments to improve this paper further.

Reviewer's comment:

1. Authors have used random intercept model for binary multilevel logistic regression. Authors can also do some more testing of their model by including random slopes for their significant predictors in this model to check if the effect of their key predictors remains the same for each cluster or is it different. If they don't find any major difference by including the random slopes, then they can provide the current results as it is.

Authors response: thanks for the detailed comment we have tested using the random slop in checking its effect and didn't find noticeable difference from the random intercept model.

Reviewer's comment:

2. Authors have used different household condition indicators such as toilet clean fuel main roof etc. than wealth index which captures the economic condition of the household better in the analysis. Is there any specific reason to adapt this strategy?

Authors response: Thank you very much for your comment. We have considered using the wealth index as an economic condition of the household in our analysis, and the wealth index variable failed to enter the multivariant multilevel mixed model since the significant level was high during the univariable multilevel mixed model.

Reviewer's comment:

3. Authors should also provide the residuals plots for their final multilevel model in the appendix.

Authors response: thanks again for the detailed comment, we have provided the residual plots for the final model in figure 6 and on page 10, line 248-249, kindly refer to the revised manuscript.

Reviewer's comment:

4. Authors have shown that there is a significant large cluster of population where U5MR is significantly higher than other areas in Figure 4. Authors can include a table with basic socio demographic profile of this high cluster region. This can be helpful to understand which population mostly suffers with high U5M in that region so that specific interventions and programmes can be targeted for those subgroups of population.

Author’s response: Thank you very much for you very constructive comment. In the descriptive statistics section example in table one already reported. We believe that no need to include the specific socio-demographic characteristics of the hot sport areas. If there any help for us we will do it in next round revision.

Reviewer's comment:

5. Authors should include a short paragraph in the discussion on the implications of their findings in context of the SDG goals.

Authors response: Thank you very much for your comment. We have accepted the comment and kindly refer to the revised manuscript specified on page 10, line 258 up to 260.

Reviewer's comment:

6. Please see the flow of your texts in the discussion once more and edit the sentences wherever required for more clarity.

Authors response: Thank you very much for your comment. We have accepted the comment and kindly refer to the description part of the revised manuscript.

Reviewer #2:

General comments:

Reviewer's comment: - This publication is of high importance because it attempts to present sophisticated analyses of a legitimate population-based survey data to highlight key risk factors for under five mortalities in Ethiopia. However, there are two problems with the manuscript: the first is that it is poorly written in English, which makes understanding of all processed hard; secondly, there are some explanations about the statistics used that (partly due to the poor English) are difficult to understand. Double check both the English and the statistical methods used (binary, bivariable? multi-level logistic regression?).

Reviewer response: thank you for your comment. We accept and kindly find the revised manuscript. When we say binary, we want to explain that our outcome variable has only the possibility of failure or success, and also with regards to bivariable, we wanted to illustrate that we first run a multilevel logistic regression model consisting of the outcome variable and one predictor variable for each predictor variables then choosing those predictors that have passed our significant level (less than the P-value of 0.2) to the multivariable multilevel logistic regression model. We used multilevel logistic regression due to the hierarchical nature of the data.

Reviewer #3:

General comments:

Reviewer's comment: - This is an engaging, insightful paper and mostly methodologically sound paper.

There is one important thing to resolve. The authors need to explain why the geospatial analysis was pertinent. Sure, it produced great maps, but the inferences that made it to the Discussion and Conclusion sections could have been made with simple aggregation instead of the employed GIS methods. If this is not the case, the authors need to explain why their approach was used and discuss that in the Discussion section.

Authors response: thank you for your detailed comment, we accepted and the main reason we employed GIS is for cost minimization and to help the policymakers to focus on the arias noted by GIS and saTscan, we have also edited in the discussion section specifically on page 11, line 300-302 in the revised manuscript.

Reviewer's comment:

The paper should undergo professional proofreading as some formulations were strange. There were some minor mistakes and suggestions, which were marked and commented on in the attached PDF.

Authors response: thank you for your comments. We accept and kindly find the revised manuscript.

Reviewer #4

Reviewer's comment: - I have reviewed the manuscript and it brings a perspective of spatial and multilevel analysis to understanding the variations in under-five mortality in Ethiopia. The multilevel analysis introduces community level variables not examined before enabling the study to consider factors beyond individual ones.

The study uses more recent data. The method of data analysis, that is, multivariate multilevel logistic regression is fine but its limitation is that it does not handle the aspect of censored data well.

Authors response: thanks for the comment, our data is cross-sectional hierarchical data with a binary outcome and for this type of data multilevel logistic regression is the best method of data analysis.

Reviewer's comment:

In performing the analysis, it is not clear in the manuscript if the data were weighted or not.

Authors response: thanks for the comment we have accepted and revised it kindly find on page 6 line 166 up to line 167 in the revised manuscript.

Reviewer's comment:

The data source section (page 4, lines 91-108), could have been summarized further and high light the information collected by the 2019 EMDHS relevant to study.

Authors response: thanks for the comment we accepted and kindly refer to the revised manuscript specifically (page 4, lines 91- 97).

Reviewer's comment:

On study variables (page 5, lines 112-122), the presentation of the variables could have been efficiently done in a table with definitions and categorization. More especially for community level variables.

Authors response: thank you for your comment, we have considered using the table to illustrate the study variables and since the variables are presented in table 1 up to 3 additional tables, we feared that an additional table might be a bit redundant.

Reviewer's comment:

There are too many variables considered at individual level. Is it possible to reduce them to relevant ones only based on reviewed literature?

Authors response: thanks for your detailed comment, all of our variables are from literature reviews and we have reduced many not significant variables during the bivariable multilevel logistic regression.

Reviewer's comment:

For spatial analysis (page 5, lines 124-125), state what was actually done. Highlight the benefits and limitations of using multilevel analysis.

Authors response: thank you for the detailed comment the spatial analysis that was employed are reported in detail on page 5-6, line 127-145.

Reviewer's comment:

In the results section (pages 8-9), is possible to use 1 decimal place for the percentages in the text. Also, check the table titles and ensure they are named appropriately.

Authors response: thanks for the detailed comment, we have considered using one decimal place and since we wanted to be precise as possible that is why we used two decimal places.

Reviewer's comment:

For multilevel analysis results, the intra-cluster correlation (ICC) is not shown in the table.

Authors response: thanks for the detailed comment, we accept and kindly find the revised table on page 24 in the revised manuscript.

Reviewer's comment:

A theoretical framework work should have been considered by the study.

Authors response: thank you for the detailed comment, we have provided the theoretical framework kindly find the revised manuscript specifically in figure 1.

Reviewer's comment:

In the discussion section of the manuscript, emphasis should have been on discussing community level factors and the geographical variation in under-five mortality observed.

Authors' response: thanks for the detailed comment, we only discussed the community factors that are significantly associated with under-five mortality, and most of the community-level factors are not significantly associated with under-five mortality.

Reviewer's comment:

The manuscript should have discussed if the under-five mortality rates in the 2019 EMDHS were reproduced for benchmarking prior to deriving its own.

Authors response: thanks for your comment we have discussed benchmarking prior to EDHS, kindly find it on page 10 between the lines 258-261.

Reviewer's comment:

The conclusion and recommendations of the manuscript should have been based on the multilevel analysis (community level variables) results in addition to the spatial variation findings on under-five mortality.

Authors' response: thanks for the detailed comment, most of the community-level variables were not significantly associated with under-five mortality that is why we didn't give focused on the community-level variables in the conclusion.

Reviewer #5

Reviewer's comment: This paper is well motivated and carries out a series of multivariate and spatial analysis on Ethiopian data on under 5 child mortality. The survey data appears to be of excellent quality from a rigorous sampling design. The spatial analysis is a strong point and allows subregional rates to be detected that are higher or lower than the background. Spatial scan statistics provide a promising approach in this case and are good complements to the other approaches.

The authors should properly cite the SaTScan software, along with the author M.Kulldforf (see the user manual). I would encourage citing Stata properly as well (note spelling and case).

Authors response: thank you for your detailed comment, we accepted kindly refer to the revised manuscript specifically page 6, lines 139 and 143.

Reviewer's comment:

In my experience, SaTScan runs need quite a bit of tinkering of the parameter file to get good hot spot representation (i.e., like that in the spatial EB approach) . I think the cluster detection can be improved by selecting a smaller window size or radius in order to detect smaller secondary clusters other than the primary two presented here.

Authors response: thanks for the detailed comment, we agree that the cluster can be improved by selecting a smaller window size to detect smaller secondary clusters but we aim to identify the primary ones that is why we used the default maximum spatial cluster size of less than 50% of the population, which allows both small and large clusters to be detected.

Reviewer's comment:

Otherwise, this is an informative paper on an important topic. Table 4 could use better formatting. Please consider using coefficient plots. Additional proofing of the manuscript is also recommended to improve clarity.

Authors response: thank you for your detailed comment, we have improved the clarity kindly refer to the revised manuscript. It known that it possible to report using coefficient plot but we believe table four format appropriate in this regard.

Reviewer #6

Reviewer's comment:

This is a thoughtful and interesting analysis of under-five mortality in Ethiopia in the years preceding the current crisis and civil war. The analysis focusses on the major individual and area level variables that have been identified in the literature and the results are largely in line with previous findings. While my overall evaluation is positive there are a number of issues that need to be addressed before publication:

1. The style of writing is extremely telegraphic, to the point that at times the meaning is not strictly clear. For instance, your community level variables refer to place of delivery; toilet use; wealth and media exposure. I presume these are based on majority characteristics from your individual level analysis, but this should be made clearer. In particular, I see no reference to media exposure at the individual level, so what does this variable refer to?

Authors response: thank you for your detailed comment, we accepted and kindly refer to the revised manuscript specifically page 5, line 115-123.

Reviewer's comment:

2. A major contribution of this paper is the difference between the different areas of Ethiopia and the relation to community characteristics. It would be important, for non-Ethiopian readers, to provide a bit more background on the different regions, socially and historically. A comparison based on the community characteristics you describe would be particularly useful. You should also provide references for the spatial analysis you present (Moran's I and the spatial interpolation).

Authors response: thank you for your comment, we didn't find any significant community characteristics that's why we didn't compare based on the community characteristics. Also, we

accepted and kindly find the revised manuscript for the edited reference on pages 5, line 130, and 135 respectively.

Reviewer's comment:

3. Your choice of model (multilevel binomial) is appropriate but the presentation is a bit overloaded. Many of the variables appear to be non-significant in the multivariate model, even if they were significant at the bivariate level. This may be due to collinearity between the variables. Removing the non-significant variables would bring out better the major covariates related to U5M.

Authors response: thanks for the detailed comment, we have checked for collinearity and there was no noticeable collinearity between variables.

Reviewer's comment:

It is also important to indicate that the coefficients are relative risks (eb). There is no need for both log-likelihood and deviance values (as you need, D = -2LL) but you should indicate the degrees of freedom and the variance of the random effects (clusters) as well as the number of individual cases (level 1) and clusters (level 2).

Authors response: thanks for the detailed comment, we accept we have edited out the deviance from the table kindly refer to the revised table 4 in the revised manuscript. As for the number of individual cases and clusters we thought it would be repetition since it has been mentioned in the data source page 4, lines 101 and 105, and also on the result section on page 8, line 194.

Reviewer's comment:

4. Your discussion focusses on the major factors reducing under 5 mortalities, but you do not discuss differences between the models. For instance, use of solid fuel increases U5M in model 2 and reduces it in model 4; Amhara has higher mortality in model 3, lower mortality in model 4. In both cases, the coefficients are non-significant (within the CI) but the reverse should still be mentioned (also see previous comment on removing non-significant comparisons).

Authors response: thanks for the detailed comment. Indeed it is advantageous to discuss the differences between the models, we feer that the paper might loos its pertinence.

Reviewer's comment:

5. Some of the comments in your discussion appear to be inconsistent. For instance, larger families have lower mortality, but so do higher birth orders. You mention mother's age at first birth but not at current birth, which may be important in explaining this paradox. You rightly identify risk factors subject to intervention, such as short breast feeding or home delivery, but I fail to see how twin births fall into this category, except in the sense that greater attention should be given to mothers with multiple births.

Authors response: thanks for the comment, Since also multiple births are prone to develop short to long-term complications kindly refer to the citation (32). We accept and we edited in multiple births and compared it with singleton birth kindly refer to the revised manuscript specifically on page 12, line 313.

Reviewer's comment:

In sum, this is a valuable contribution which I hope to see published, and my comments are intended to strengthen what is, at heart, a sound analysis based on a rich and valuable dataset.

Reviewer #7

Reviewer's comment:

The paper is sound and generally well-written. The topic is very relevant and the analyses performed compatible and consistent with the research question. I have some minor remarks that I consider should be addressed before approving for publication:

Overall, please explain a bit further the difference between the mini-DHS survey and the main DHS survey. Why do you think this new data is helpful to track the trends in U5M? The authors extensively explain the sampling design, but do not explain the difference between the mini and the main DHS survey, which would be helpful in interpreting the results. Is the mini-DHS survey comparable to the main DHS survey? Can we use this to compare them in time?

Authors response: thanks for the detailed comment. Indeed it is advantageous to discuss the differences between the mini-DHS and main DHS, we feer that the paper might loos its pertinence.

Reviewer's comment:

In addition, it would be important to know whether any data quality assessment of deaths was performed before the regression analysis. Would any bias be added to the study due to misreporting of deaths in the survey or issues related to coverage areas? Did the authors correct the registry of deaths in any way or were deaths under 5 taken as it is from the survey? How do the authors feel about this data and do they trust the information?

Authors response: Thank you very much for your ideal comment. Frankly speaking, we authors had no any room to check death registry. Since is secondary data and there is no strong death registry in the country it is difficult to check death by any other means. Before the data is collected training was given for data collectors this may be one way data quality assurance. The second it large survey and conducted carefully with expertise and representative of the country under-five mortality. The information is trustful.

Reviewer's comment:

In addition, some minor remarks/suggestions in the writing part that felt confusing:

lines 82- 84: the sentence is confusing. I would add here a period. and Say: "Though U5M has been declining, it is still high and is taking the lives of many children. Despite this fact, no research on U5M has been done with recent data available from the Ethiopian mini ... "

Authors response: thank you for your detailed comment, we accepted and kindly find the edited statements on pages 3 and 4, lines 81 – 84.

Reviewer's comment:

lines 85-87: similarly, the sentence is confusing. I would suggest: "...to show the current burden of U5M that is crucial for better planning different policies and interventions for U5M prevention. This allows for an efficient allocation of scarce resources according to spatial..."

Authors response: thank you for your detailed comment, we accepted and kindly find the edited statements on page 4 lines 85 – 88.

Reviewer's comment:

lines 188: suggestion to make the sentence clearer: "Sociodemographic background" and not "background socio-demographic"

Authors response: thank you for your detailed comment, we accepted and kindly find the edited statements on page 8, line 193.

Reviewer's comment:

line 195: correct to: "are of poor economic status"

Authors response: thank you for your detailed comment, we accepted and kindly find the edited statements on page 8, line 199.

Reviewer #8

Reviewer's comment:

This paper aims to examine geographic variation and factors attributable to Under-Five Mortality (UFM) in Ethiopia. Recognizing as an important marker for health equity and access, UFM is considered the best proxy measure or indicator for socioeconomic development. In addition, child mortality rate is also a useful marker of overall development and a Millennium Development Goal (MDG) indicator and its importance has been further emphasized in an ambitious target under the Sustainable Development Goals. Generally, literature search reveals numerous or countless studies on factors determining UFM conducted in the Sub-Saharan African countries, however, spatial distributions and geographic variations are less investigated compared to the UFM factors.

The data sources used, statistical tests and analysis performed systematically support, but NOT to the full extent, in answering the research questions and its intended aims or objectives. Results and conclusion made in relation to research aims and objectives or intentions are supported by data and analysis, in general. However, there needs further refinement and improvement for author(s) to take into the consideration to make this paper more added-value to the knowledge and literature.

I foresee two major drawbacks of this study, which are described below:

Reviewer's comment:

1. The Primary Investigator should not limit the independent variables to individual and community level predictors. It is suggested to take into the consideration of inclusion of health interventions, such as malaria, sanitation and hygiene (WASH), reproductive health (RH), vaccinations, micronutrient supplementation and treatments. Most studies have revealed that there are significant association between these health interventions and reduction of UFM. For example, BCG, OPV, Measles, TT, etc. vaccinations have significantly contributed to drastic reduction in deaths of children in most developing countries. Furthermore, measure DHS captures or collects information on health interventions; and it's worthwhile to examine its association with UFM in Ethiopia.

Authors response: thank you for the detailed comments, we have considered using the above-listed variables, and some we used like WASH variables (time to water), and contraceptive utilization, but still some variables like (water source and toilet type) were not able to pass the bivariable multilevel logistic regression and some variables like (BCG, OPV, measles, and TT) were no available in the EMDHS data.

Reviewer's comment:

2. Since the other known predictors of child morality are generally attributable to food security and accessibility in conflict areas thus, it is recommended to discuss how such conflicts or disruptions would influence or alter the geographic variations of UFM in Ethiopia? Other risk factors to take into considerations are: indoor air pollution (determined by type of cooking fuel used in household), nutrition, access to basic health services (ANC, PNC, FP, RH, etc.), poverty status by regions, fertility rates, among others.

Authors response: thank you for the detailed comments, the EMDHS data doesn't possess the data that is attributable to food security and whether the EA was a conflict area at the time of collecting the data. additionally, we have considered indoor air pollution and added the variable of cooking fuel to the final models but the results were insignificant and we didn't want to discuss its results. We have also considered basic health services and most were insignificant during the bivariable multilevel logistic regression model and were not added to the multivariable multilevel logistic regression model.
