# Supplementary material for: Geographic variation and factors associated with under-five mortality in Ethiopia. A spatial and multilevel analysis of Ethiopian mini demographic and health survey 2019
Source: PLoS One. 2022 Oct 13;17(10):e0275586. doi: 10.1371/journal.pone.0275586 (PMC9560495; doi:10.1371/journal.pone.0275586)
Supplement: Attachment — Submitted filename: PONE-D-21-20589_R2-AFort.pdf [file pone.0275586.s006.pdf]

PLOS ONE

Geographic variation and factors associated with under-five mortality in Ethiopia. A spatial and multilevel analysis of Ethiopian Mini Demographic and Health Surveys 2019.

--Manuscript Draft--

**Manuscript Number:**	PONE-D-21-20589R2
**Article Type:**	Research Article
**Full Title:**	Geographic variation and factors associated with under-five mortality in Ethiopia. A spatial and multilevel analysis of Ethiopian Mini Demographic and Health Surveys 2019.
**Short Title:**	Geographic variation and factors associated with under-five mortality in Ethiopia

**Corresponding Author:**	Zemenu Tadesse Tessema, MSC Biostatistics<br>University of Gondar<br>Gondar, ETHIOPIA

**Keywords:**	Under five mortality; factors; spatial distribution; multilevel; Ethiopia

Abstract:

Background

The distribution of under-five mortality (U5M) worldwide is uneven and the burden is higher in Sub-Saharan African countries which account for more than 53% of the global under-five mortality. In Ethiopia, though U5M shows a substantial decrease from 1990 to 2019, it is still unacceptably high and unevenly distributed. Therefore, this study aimed to assess geographic variation and factors associated with under five mortality in Ethiopia.

Method

We sourced data from the most recent nationally representative 2019 Ethiopian mini Demographic and Health Survey data for this study. A sample size of 5,695 live births were considered for this study. Descriptive and analytical Analysis was conducted by using STATA version 16. Both multilevel and spatial analyses were employed to ascertain the factors associated with under-five mortality in Ethiopia.

Result

The under-five mortality is 5.9% with its 95% CI 5.4% to 6.6%. Based on the multivariable multilevel logistic regression model result; family size (AOR=0.09, 95%CI:(0.84, 0.99)), number of under-five children in the family (AOR=0.17, 95%CI: 0.14, 0.21), multiple birth (AOR=14.4, 95%CI: (8.5, 24.3)), Children who breastfed for less than 6 months (AOR=5.04, 95%CI: (3.81, 6.67)), People whose main roof is palm (AOR=0.57, 95%CI: 0.34, 0.96), Under-five children who are at birth order sixth or more (AOR=2.46, 95%CI: (1.49, 4.06)), institutional delivery (AOR=0.57, 95%CI: (0.41, 0.81)), being resident of Somali and Afar region (AOR=3.46, 95%CI: (1.58, 7.55)) and (AOR=2.54, 95%CI: (1.10, 5.85)) respectively were factors associated with under-five mortality. A spatial analysis result revealed that hot spot areas of under-five mortality were located in Somali region and Dire Dawa.

Conclusion

Under-five mortality in Ethiopia is 59 per 1000 live births which is high and unacceptable as compared to the 2030 agenda for sustainable development aimed at 25 per 1000 live births. ~~Breast feed~~ less than 6 month, twin birth, institutional delivery and high risk areas of under-five mortality (Somali and ~~dire~~ Dawa) were modifiable risk factors. Therefore maternal education on the advantage of breast feeding and institutional delivery is highly recommended. Women who deliver twins should be given special attention. An effective strategy should be designed for intervention ~~for~~ hot spot areas of under-five ~~mortality that is~~ Somali and Dire Dawa.

**Order of Authors:**	Zemenu Tadesse Tessema, MSC Biostatistics

Powered by Editorial Manager® and ProduXion Manager® from Aries Systems Corporation

	Tsion Mulat Tebeje
	Lewi Goytom Gebrehewet
**Opposed Reviewers:**	
**Response to Reviewers:**	PLOS ONE<br>Point by point response for editors/reviewers' comments<br>The manuscript title "Geographic variation and factors associated with under-five mortality in Ethiopia. A spatial and multilevel analysis of Ethiopian Mini Demographic and Health Surveys 2019"<br>Manuscript number: PONE-D-21-20589R2<br>Dear editor/reviewer,<br>I would like to thank you for these constructive, building, and improvable comments on this manuscript that would improve the substance and content of the manuscript. We considered each comment and clarification question of reviewers on the manuscript thoroughly. My point-by-point responses for each comment and question are described in detail on the following pages. Further, the details of changes were shown by track changes in the supplementary document attached.<br><br>Reviewer comment and authors response<br>Reviewer #1:<br>General comments:<br>Reviewer's comment: - THIS IS A GOOD STUDY BUT IS TERRIBLY WRITTEN! THE ONLY WAY IT WILL BE PUBLISHED IS THAT THE AUTHORS LOOK IN DETAIL AT ALL THE IMPRECISIONS AND BADLY WRITTEN CONTENT. ONLY IF THEY RE-WRITE ENTIRELY WITH AN ENGLISH-SPEAKING PERSON (WHOSE FIRST LANGUAGE IS ENGLISH OR IS VERY PROFFICIENT IN ENGLISH), THIS MANUSCRIPT WILL BE PUBLISHED. OTHERWISE, UNFORTUNATELY, IT CANNOT BE PUBLISHED! PLEASE PAY CLOSE ATTENTION TO ALL THE WRITING<br>Authors response: Thank you very much for you very important comment specially the language of the manuscript and recommendation to give our manuscript for language editor to English speaking person. We accept your comment and gave our manuscript for English speaking person (Elizabeth Korevaar). We acknowledge her support in our revised manuscript. She gave us extensive language edition and we corrected it in the revised version of the manuscript.<br><br>Reviewer's comment: The comment of the reviewer was given on the PDF part of the manuscript with detail comments.<br>Authors response: - We accept all comments and corrected it accordingly.
**Additional Information:**	
**Question**	**Response**
**Financial Disclosure**<br><br>Enter a financial disclosure statement that describes the sources of funding for the work included in this submission. Review the submission guidelines for detailed requirements. View published research articles from *PLOS ONE* for specific examples.<br><br>This statement is required for submission and **will appear in the published article** if the submission is accepted. Please make sure it is accurate.	The authors received no specific funding for this work.

**Unfunded studies**<br>Enter: *The author(s) received no specific funding for this work.*<br><br>**Funded studies**<br>Enter a statement with the following details:<br>• Initials of the authors who received each award<br>• Grant numbers awarded to each author<br>• The full name of each funder<br>• URL of each funder website<br>• Did the sponsors or funders play any role in the study design, data collection and analysis, decision to publish, or preparation of the manuscript?<br>• **NO** - Include this sentence at the end of your statement: *The funders had no role in study design, data collection and analysis, decision to publish, or preparation of the manuscript.*<br>• **YES** - Specify the role(s) played.<br><br>* typeset	
**Competing Interests**<br><br>Use the instructions below to enter a competing interest statement for this submission. On behalf of all authors, disclose any competing interests that could be perceived to bias this work—acknowledging all financial support and any other relevant financial or non-financial competing interests.<br><br>This statement is required for submission and **will appear in the published article** if the submission is accepted. Please make sure it is accurate and that any funding sources listed in your Funding Information later in the submission form are also declared in your Financial Disclosure statement.<br><br>View published research articles from *PLOS ONE* for specific examples.	The authors have declared that no competing interests exist.

**NO authors have competing interests**<br><br>Enter: *The authors have declared that no competing interests exist.*<br><br>**Authors with competing interests**<br><br>Enter competing interest details beginning with this statement:<br><br>*I have read the journal's policy and the authors of this manuscript have the following competing interests: [insert competing interests here]*<br><br>* typeset	
**Ethics Statement**<br><br>Enter an ethics statement for this submission. This statement is required if the study involved:<br><br>• Human participants<br>• Human specimens or tissue<br>• Vertebrate animals or cephalopods<br>• Vertebrate embryos or tissues<br>• Field research<br><br>Write "N/A" if the submission does not require an ethics statement.<br><br>General guidance is provided below. Consult the submission guidelines for detailed instructions. **Make sure that all information entered here is included in the Methods section of the manuscript.**	Ethical clearance was obtained from measure DHS through filling requesting a form for accessing data. The data used in this study are publicly available, aggregated secondary data that hasn't any personal identifying information that can be linked to study participants. The confidentiality of data was maintained anonymously.

Powered by Editorial Manager® and ProduXion Manager® from Aries Systems Corporation

**Format for specific study types**<br><br>**Human Subject Research (involving human participants and/or tissue)**<br>• Give the name of the institutional review board or ethics committee that approved the study<br>• Include the approval number and/or a statement indicating approval of this research<br>• Indicate the form of consent obtained (written/oral) or the reason that consent was not obtained (e.g. the data were analyzed anonymously)<br><br>**Animal Research (involving vertebrate animals, embryos or tissues)**<br>• Provide the name of the Institutional Animal Care and Use Committee (IACUC) or other relevant ethics board that reviewed the study protocol, and indicate whether they approved this research or granted a formal waiver of ethical approval<br>• Include an approval number if one was obtained<br>• If the study involved *non-human primates*, add *additional details* about animal welfare and steps taken to ameliorate suffering<br>• If anesthesia, euthanasia, or any kind of animal sacrifice is part of the study, include briefly which substances and/or methods were applied<br><br>**Field Research**<br><br>Include the following details if this study involves the collection of plant, animal, or other materials from a natural setting:<br>• Field permit number<br>• Name of the institution or relevant body that granted permission	
**Data Availability**<br><br>Authors are required to make all data underlying the findings described fully available, without restriction, and from the time of publication. PLOS allows rare exceptions to address legal and ethical concerns. See the PLOS Data Policy and FAQ for detailed information.	Yes - all data are fully available without restriction

A Data Availability Statement describing where the data can be found is required at submission. Your answers to this question constitute the Data Availability Statement and **will be published in the article**, if accepted.<br><br>**Important:** Stating 'data available on request from the author' is not sufficient. If your data are only available upon request, select 'No' for the first question and explain your exceptional situation in the text box.<br><br>Do the authors confirm that all data underlying the findings described in their manuscript are fully available without restriction?	
**Describe where the data may be found in full sentences. If you are copying our sample text, replace any instances of XXX with the appropriate details.**<br><br>• If the data are **held or will be held in a public repository**, include URLs, accession numbers or DOIs. If this information will only be available after acceptance, indicate this by ticking the box below. For example: *All XXX files are available from the XXX database (accession number(s) XXX, XXX.).*<br>• If the data are all contained **within the manuscript and/or Supporting Information files**, enter the following: *All relevant data are within the manuscript and its Supporting Information files.*<br>• If neither of these applies but you are able to provide **details of access elsewhere**, with or without limitations, please do so. For example:<br><br>*Data cannot be shared publicly because of [XXX]. Data are available from the XXX Institutional Data Access / Ethics Committee (contact via XXX) for researchers who meet the criteria for access to confidential data.*<br><br>*The data underlying the results presented in the study are available from (include the name of the third party*	Third party data was obtained for this study from The DHS Program. Data may be requested from The DHS Program after creating an account and submitting a concept note. More access information can be found here: https://dhsprogram.com/data/Access-Instructions.cfm<br><br>The authors confirm that interested researchers would be able to access these data in the same manner as the authors. The authors also confirm that they had no special access privileges that others would not have.

and contact information or URL).<br>• This text is appropriate if the data are owned by a third party and authors do not have permission to share the data.<br><br>* typeset	
Additional data availability information:	

Powered by Editorial Manager® and ProduXion Manager® from Aries Systems Corporation

March 23/ 2022

Gondar, Ethiopia

Subject: **Cover Letter**

Dear editors,

We intended to submit our research manuscript entitled **“Geographic variation and factors associated with under-five mortality in Ethiopia. A spatial and multilevel analysis of Ethiopian Mini Demographic and Health Surveys 2019. ”** in your journal **PLOS ONE** as a research article. The reason for considering is for its open access, the fast publication process, rigorous peer-review, and high visibility. The manuscript was not submitted elsewhere. In this regard, we request you to kindly consider our manuscript to publish in your esteemed journal.

Regarding

Editors Comment : Are these third party data (i.e., data not owned or collected by the author(s))?

Authors response:- Yes , the data is obtained from third pary that is fronm DHS program after a short project concept submitted. Therefore other authors can access this dataset from DHS program after create an account and prepare a concept note that they are interested. The program websiate is www.dhsprogram.com

Editors Comment:-Figures

Authors response:-The figures in the manuscript was obtained from WWW.OpenAfrica Shapfile free and obtained from this website

Best Regards:

Zemenu Tadesse Tessema,

March 23/ 2022

Gondar, Ethiopia

Subject: **Cover Letter**

Dear editors,

We intended to submit our research manuscript entitled **"Geographic variation and factors associated with under-five mortality in Ethiopia. A spatial and multilevel analysis of Ethiopian Mini Demographic and Health Surveys 2019. "** in your journal **PLOS ONE** as a research article. The reason for considering is for its open access, the fast publication process, rigorous peer-review, and high visibility. The manuscript was not submitted elsewhere. In this regard, we request you to kindly consider our manuscript to publish in your esteemed journal.

Regarding

Editors Comment : Are these third party data (i.e., data not owned or collected by the author(s))?

Authors response:- Yes , the data is obtained from third pary that is fronm DHS program after a short project concept submitted. Therefore other authors can access this dataset from DHS program after create an account and prepare a concept note that they are interested. The program websiate is www.dhsprogram.com

Editors Comment:-Figures

Authors response:-The figures in the manuscript was obtained from WWW.OpenAfrica Shapfile free and obtained from this website

Best Regards:

Zemenu Tadesse Tessema,

Manuscript

Click here to access/download;Manuscript;revised final manuscript_LK.docx

Geographic variation and factors associated with under-five mortality in Ethiopia. A spatial and multilevel analysis of Ethiopian Mini Demographic and Health Surveys 2019.

Zemenu Tadesse Tessema [1,2*], Tsion Mulat Tebeje[3], Lewi Goytom Gebrehewet[2]

[1]Department of Epidemiology and Preventive Medicine, School of Public Health and Preventive Medicine, Monash University, Melbourne, Australia

[2] Department of Epidemiology and Biostatistics, Institute of Public Health, College of Medicine and Health Sciences, University of Gondar, Gondar, Ethiopia

[3] Epidemiology and Biostatistics Unit, School of public health, Dilla University, Dilla, Ethiopia

E-mail: ZTT zemenut1979@gmail.com

TMT yemarina12@gmail.com

LGG lewigt369@gmail.com

***= Corresponding author**

Abstract

Background

The distribution of under-five mortality (U5M) worldwide is uneven and the burden is higher in Sub-Saharan African countries, which account for more than 53% of the global under-five mortality. In Ethiopia, though U5M decreased substantially between 1990 and 2019, it remains excessively high and unevenly distributed. Therefore, this study aimed to assess geographic variation and factors associated with under-five mortality (U5M) in Ethiopia.

Methods

We sourced data from the most recent nationally representative 2019 Ethiopian Mini-Demographic and Health Survey for this study. A sample size of 5,695 total births was considered. Descriptive, analytical analysis and spatial analysis were conducted using STATA version 16. Both multilevel and spatial analyses were employed to ascertain the factors associated with U5M in Ethiopia.

Results

The U5M was 5.9% with a 95% CI 5.4% to 6.6%. Based on the multivariable multilevel logistic regression model results, the following characteristics were associated with under-five mortality: family size (AOR=0.92, 95%CI : 0.84,0.99), number of under-five children in the family (AOR=0.17, 95%CI: 0.14, 0.21), multiple birth (AOR=14.4, 95%CI: 8.5, 24.3), children who were breastfed for less than 6 months (AOR=5.04, 95%CI: 3.81, 6.67), people whose main roof is palm (AOR=0.57, 95%CI: 0.34, 0.96), under-five children who are the sixth or more child to be born (AOR=2.46, 95%CI: 1.49, 4.06), institutional delivery (AOR=0.57, 95%CI: 0.41, 0.81), resident of Somali and Afar region (AOR=3.46, 95%CI: 1.58, 7.55) and (AOR=2.54, 95%CI: 1.10, 5.85), respectively. Spatial analysis revealed that hot spot areas of under-five mortality were located in the Dire Dawa and Somali regions.

Conclusion:

Under-five mortality in Ethiopia is high and unacceptable when compared to the 2030 sustainable development target, which aims for 25 per 1000 live births. Breastfeeding for less than 6-months, twin births, institutional delivery and high-risk areas of under-five mortality (Somali and dire

Dawa) are modifiable risk factors. Therefore, maternal education on the advantages of breastfeeding and institutional delivery is highly recommended. Women who deliver twins should be given special attention. An effective strategy should be designed for intervention in under-five mortality hot spot areas such as Somali and Dire Dawa.

Keywords: Under-five mortality, factors, spatial distribution, multilevel, Ethiopia

Background

Under-five mortality is defined as deaths reported at ages 0 to 59 months and includes neonatal, post-neonatal, and child deaths (1). ~~Under-five mortality rate (U5MR) is the probability of a child dying before celebrating their fifth birthday~~(2). It is a significant indicator of the socioeconomic, health, and environmental conditions, and ~~the~~ national development ~~of~~ health equity and access(3).

Globally, in 1990, the number of under-five deaths was 12,494,000 (93 deaths per 1000 live births). Following an average annual decline of 3.1%, by 2019 it was significantly reduced to 5,189,000 deaths (38 deaths per 1000 live births) representing 58% reduction(4). The 2030 sustainable development (SDG~~?~~) aims to end the preventable deaths of newborns and children under 5 years of age, with all countries aiming to reduce under-5 mortality to at least25 per 1000 live births or lower(5).

The distribution of U5MR worldwide is uneven and the global burden of U5MR is concentrated in two regions, Sub-Saharan Africa and Central and Southern Asia, which account~~s~~ for more than 80% of U5MR(6). The burden is even higher in Sub-Saharan African countries with 74 deaths per 1000 live births, which account~~s~~ for more than 53% of the global under-five mortality(7).

In Ethiopia, U5MR has substantially decreased from 200 per 1000 live births in 1990 to 49 per 1000 live births in 2020 but is not close to Sustainable Development~~(SDG)~~(4). Additionally, different studies conducted in Ethiopia revealed several socioeconomic, demographic, and geographic or spatial variation. For example, Afar, Somali, and Benishangul Gumuz are the three high-risk regions of Ethiopia for U5MR(8).

Different studies examining U5M previously showed that source of water(9), multiple births (7, 8), region (10, 11), age and sex of household head (11), maternal education status (12, 13), place of delivery(10, 11, 14), maternal age(15-17), sex of the child(9, 14, 17), residence (18, 19), family

size (10, 16), number of children under five(19, 20), birth order (10, 16), and breastfeeding status (19, 21) have a significant association with under-five mortality.

Although U5M has been declining it remains high and takes the lives of many children, no research on U5M has been made until recently, with the Ethiopian mini–Demographic Health Survey (EMDHS) 2019.Research is required to inform policies and interventions for the prevention of U5M with the efficient allocation of scarce resources to priority areas based. This study aimed to assess the geographic variation and factors associated with under-five mortality in Ethiopia. The results of our study will assist in achieving the SDG, by providing an understand of the current burden of U5M in Ethiopia, and its determinants, in addition to identifying areas and factors with the highest burden of U5M in Ethiopia.

Method

Data Source

Our source was the 2019 EMDHS, the second mini demographic health survey (DHS) conducted in Ethiopia (a land-locked country located in the Horn of Africa that lies between the 30N and 150N Latitude or 330E and 480E Longitude)(22). Data collection was conducted from March 21, 2019, to June 28, 2019, from the nine regions (Tigray, Afar, Amhara, Oromia, Somali, Benishangul Gumuz, Southern nation nationalities and People region (SNNPR), Harari, and Gambella) and two administrative cities (Addis Ababa and Dire Dawa). The study design was a population-based cross-sectional study. A frame of all census Enumeration areas (EAs) was used as a sampling frame for the 2019 EMDHS. 149,093 EAs were created which cover an average of 131 Households (HHS). A two-stage stratified cluster sampling technique was employed and each region was stratified into urban and rural areas, yielding 21 sampling strata were selected independently in each stratum. In the first stage, 305 clusters (93 urban and 212 rural) were selected with probability proportional to EAs size and with independent selection in each sampling stratum. In the second stage, a fixed number of 30 households per cluster were selected. Finally, women aged 15–49 in 9,150 (2,790 urban and 6,360 rural) households from 305 clusters were selected. The whole procedure of sampling is found in the full 2019 EMDHS report(22).

Study variables

The outcome variable was under-five mortality status, which was categorized as (child alive: Yes= 0 and No = 1). The age was recorded in months. The ~~independent variables, classified as individual-level and~~ community-level predictors, were place of residence, region, community place of delivery, community wealth, community media exposure, and community toilet facility. The individual-level predictors were further categorized as socio-demographic and economic factors like the educational level of the mother, sex of the household head, age of household head, number of household members, number of childred under the age of five, marital status of the mother, source of water, time to get water, type of toilet facility, household electricity, types of cooking fuel, main floor material, main wall material, and main roof material and maternal and child factors (such as maternal age at first birth, sex of the child), utilization of contraception, order of birth, mode of delivery, duration of breastfeeding and multiple births (**Figure 1)**.

Spatial analysis

The data for spatial analysis was cleaned and merged using STATA version 16 and Microsoft Excel. ArcGIS version 10.8 and saTScan version 9.7 were used for the spatial analysis.

Spatial autocorrelation

Spatial autocorrelation (Global Moran's I) analysis was conducted to examine whether under-five mortality was dispersed (Moran's I value closer to -1), clustered (Moran's I value closer to 1), or randomly distributed (Moran's I value of 0) in Ethiopia (23).

Spatial interpolation

The under-five mortality was known in enumerated areas, while in areas that were not selected, the under-five mortality rates were predicted. Spatial interpolation was applied using the geostatistical ordinary Kriging spatial interpolation technique to predict under-five mortality from existing sample data points to un-sampled areas (24).

Spatial scan statistics

The scan analysis was performed using SaTscan, based on the Bernoulli test for cases (child is not alive) and controls (child is alive). The upper limit used was the default maximum spatial cluster size of less than 50% of the population, allowing both small and large clusters to be detected, while clusters that contained more than the maximum limit with the circular shape of the window were avoided(25). Most likely clusters were identified using p-values and likelihood ratio tests, which is the ratio of the likelihood of the alternative hypothesis (higher activity level inside the window) over the likelihood of the null hypothesis (same activity level inside and outside).

Data management and analysis

We used STATA version 16 and R statistical software version 4.0.5 to analyze the data. A total of 31 variables were retained for the analysis. Residents who were not De jure were excluded which affected under-five mortality, as they could not respond to most of the socio-demographic and economic characteristics though they could answer the maternal and child characteristics. This exclusion changed our sample size from 5,753 to 5,695.

The outcome variable was re-coded to (child alive: Yes= 1 and No = 0). Four community variables were generated by taking the individual variable, calculating their proportion, and dichotomizing them based on their mean or median according to their distribution.

In the end, we had 29 predictor variables, of which 6 were community-level predictor variables and 23 were individual-level predictor variables.

Based on EMDHS, respondents in the same cluster showed similar outcomes or functions at the same level and the data has a hierarchical structure. This made binary logistic regression not the most appropriate as it violates the assumption of independence of the residuals. Instead, a model that considers clustering effect should be used(8, 11). Multilevel logistic modeling separates the within-cluster effects from the between-cluster effects (26). Therefore, to assess the predictors associated with U5M, a non-weighted multilevel logistic regression model was used.

Bivariable multilevel logistic regression was used to screen each predictor variable for a p-value less than 0.2. Significant variables were included in multivariable multilevel logistic models. Twenty predictor variables (3 of which were community-level predictor variables) were included

in the multivariable analysis. In the multivariable analysis, a p-value less than 0.05 was considered a factor associated with U5M.

The first model fitted was the null model (intercept model), which contained the outcome variable only (under-five mortality status) with the cluster number. The intra-cluster correlation (ICC) was used to assess whether there was a random effect. An ICC of 0.130 which meant there was a minimum of 13% under-five mortality was explained by between-cluster differences. We found that 87% of under-five mortality was explained by within-cluster differences, which was not negligible. The second model was fitted using the outcome, the cluster and the individual-level predictor variables only. The probability of U5M was predicted as a function of individual-level predictors.

For the third model the outcome variable, the cluster number, and the community-level predictor variables were accounted for. Then the final model was fitted by taking both the individual-level and the community-level predictor variables into account.

The models were compared by using a log-likelihood statistic, where the best model was selected based on smallest deviance.

Ethical consideration

Permission for data access was obtained from a major demographic and health survey through an online request from http://www.dhsprogram.com. The data used for this study were publicly available with no personal identifier.

Results

Socio-demographic background and maternal and child health factor

A total of 5,695 live births were included in this study, of which 2,942 (51.66%) were male, 5,528 (97.07%) were single children, 2,937 (51.57%) were delivered at home and 6.04% of the children were born via cesarean section (**Table 1**).

Out of the total children's mothers, most were married (5,346, 93.87 %) and 3,135 (55.05%) had no education, only 291 (5.11%) attained higher education, and 51.62% were of poor economic status. 64.27% had access to improved water; 30.99 % travel more than 30 minutes, and 50.85% travel less than 30 min to get water unlike those who do not have water on-premises. The majority of household heads were male, 4,561 (80.09%), and the most common age of the household head was 35-50 years (**Table 1**).

Among the population, the majority were rural residents 4,389 (77.07%), and 3,457 (60.70%) in the community practiced home delivery. The number of communities that used unimproved toilets was 3,175 (55.75%). 3,391 residents of the population (59.54%) were not exposed to the media (**Table 2**).

Prevalence of under-five mortality

The under-five mortality was 5.9% (95% CI 5.4%, 6.6%) and it varied across regions ranging from 2.13% in Addis Ababa to 9.82% in the Somali region. Out of the total, there were 918 (15.79%) children who died having been breastfed for less than 6 months (Table 3).

Spatial autocorrelation

The global Moran's index was 0.09, *z*-score 3.5, and p-value <0.001 suggesting that the spatial distribution under-five mortality was not at random in the 2019 EMDHS (**Figure 2**)(27).

Hotspot analysis

A high proportion of U5M was observed around the regions of Somali and Dire Dawa (**Figure 3**)(27).

Spatial Interpolation

The spatial interpolation or prediction analysis result showed that the under-five mortality ranged from 0% to 50%. The areas predicted to have the highest risk were located in Somali, Dire Dawa, Benishangul Gumuz, Gambella, and SNNPR (**Figure 4**)(27).

Spatial scan statistics analysis

As observed in figure 5, significant clusters were identified in the Somali region. Located within 7.384788 N, 45.908936 E with 612.75 km radius. Children living in the primary cluster were 62% more likely to die before they celebrate their fifth birthday when compared to those who lived outside the window (relative risk (1.62), likelihood ratio (7.36), and p-value (0.03)) (**Figure 5**)(27).

Factors associated with under-five mortality

After filtering, all variables in bivariable multilevel logistic regression, 4 models were fitted (null model, individual-level model, community-level model, and both individual- and community-level model). The ICC was 0.13 with 95% CI: 0.08 to 0.2, which is not negligible. The 4th model was the best-fitting model with the lowest deviance. When the number of household members increased by one, the odds of under-five mortality decreased by 8.5% (AOR=0.92, 95%CI: 0.84, 0.99). The odds of under-five death decreased by 82.8% (AOR=0.17, 95%CI: 0.14, 0.21) as the number of under-five children in the family increased by one. Children who were part of multiple births had 14 times higher odds of under-five mortality (AOR=14.4, 95%CI: 8.5, 24.3) than singletons. Children who were breastfed for less than 6 months had 5 times higher odds of experiencing under-five mortality (AOR=5.04, 95%CI: 3.81, 6.67) than those who were breastfed. People whose main roof was palm/bamboo had 43% (AOR=0.57, 95%CI: 0.34, 0.96) decreased odds of having under-five deaths in the family than those with corrugated iron. Under-five children who were the sixth born or more ~~were~~ 2.5 (AOR=2.46, 95%CI: 1.49, 4.06) times higher odds of under-five mortality compared to the second and third born. Place of delivery was found to have a significant association with U5M, those who were delivered in institutions had 43% less chance of experiencing U5M (AOR=0.57, 95%CI: 0.41, 0.81) compared to those delivered at home. Being a resident of the Somali and Afar region increased the odds of under-five death by 3.5 (AOR=3.46, 95%CI: 1.58, 7.55) and 2.5 (AOR=2.54, 95%CI: 1.10, 5.85) times, respectively, compared to the Tigray region (Table 4).

The residual plot shows that the model performed adequately, with the Kolmogorov shirnov(KS)test being insignificant (p-value = 0.29), and also the deviation was not significant (**Figure 6**).

Discussion

The study mainly focused on the spatial distribution of under-five mortality and on factors that influence it. The findings of this study revealed that the prevalence of under-five mortality was 5.9% (95% CI: 5.4, 6.6). The multivariable multilevel logistic regression model result showed that family size, number of under-five children, birth order, breastfeeding, multiple birth, region, and institutional delivery were associated with under-five mortality in Ethiopia. The spatial analysis result revealed that Somali, Dire Dawa and Eastern Oromia were high-risk areas for under five ~~mortalities~~ in Ethiopia.

Increased number of family size resulted in decreased odds of under-five death. This finding is consistent with previous studies conducted in Ethiopia(17), Eastern Nigeria(28), and Ghana(29). This may be due to the mother of the children being more experienced in taking good and adequate care of the children as the family size increases, in addition to there being more family members that can care for under-five children. ~~Another possible reason might be that the mother may be more experienced and may learn from her previous mistakes if there were any~~. However, this is in contrast to another study conducted in Ethiopia, which demonstrated that the odds of under-five mortality increased as the number of under-five children increased(19). This may indicate that , as the number of under-five children increases, it may become difficult to fulfill their basic needs, and their family too exhausted to look after the children.

Birth order had significant effect on under-five mortality. This study indicates that, as order of birth increased, the likelihood of child death also increased. This finding is consistent with a study conducted in rural parts of Ethiopia (10). However, it does not support another study conducted in Ethiopia which found that being firstborn increased the odds of under-five mortality twofold when compared to those with five and above birth orders(19). This may be because firstborns are more likely to be hospitalized as a result of congenital malformations and perinatal conditions in their early childhood. This changes for firstborn children as they get older, while younger brothers and

sisters are more likely to be hospitalized for injuries and avoidable conditions, indicating less parental care (30).

Being ~~B~~reastfed forless than six months increased the odds of under-five mortality compared with those who were breastfed for six and more months. This is consistent with a finding in Ethiopia(19). Breastfeeding is known as a ~~prevention~~ method for reducing child mortality and as well as a prevention ~~of~~ delayed growth (31, 32).

Being multiple birth children increased the odds of under-five mortality when compared to singleton children. This finding is in line with several studies conducted in Ethiopia at different times, and a study conducted in rural parts of Ethiopia(7, 8, 10, 17, 19). This may be due to multiple births imposing short- and long-term medical complications as well as adding increased burden on the mother and the family (33).

Facility or institutional delivery reduced the odds of under-five mortality by 43% as compared to home delivery. This finding is consistent with studies conducted in Ethiopia(10, 11) and the Democratic Republic of Congo(14). This is because home delivery increases the death of under-five children due to a higher risk of postpartum complications. These complications include exposure to common vaccine-preventable diseases if there is absence of vaccination, and a lower likelihood of attending prenatal and postpartum visits (11, 34).

The odds of under-five mortality ~~was~~ higher for residents of Afar and Somali regions compared to the Tigray region. This coincides among other things, with Somali and Afar regions having the first and second lowest coverage of children with all basic ~~vaccinations. There is serious child malnutrition with the first and second highest prevalence of wasting in the Somali and Afar~~, respectively(35, 36). Additionally, the Somali region was one of the regions that were in the primary cluster from our spatial scan statistics analysis where under-five mortality is more likely to occur. This is not supported by studies conducted in Ethiopia based on the 2011 and 2016 EDHS(10, 11), where other regions were found to have a significant association with under-five mortality. This discrepancy may be a result of the different number of enumerated areas (clusters) used in the different studies, which may have led to different samples, or different statistical models used for analysis.

Conclusion: Under-five mortality in Ethiopia was 5.9% of live birth. Being breastfed for more than 6-months, twin birth, institutional delivery and high-risk areas of U5M (Somali and dire Dawa) were modifiable risk factors. Therefore, maternal education on the advantage of breastfeeding and institutional delivery is highly recommended. Women who deliver multiple births should be given special attention. Further, effective strategies should be designed for intervention on hot spot areas of U5M, namely Somali and Dire Dawa.

Strength and limitation of the study

We used EMDHS 2019 data which is more inclusive and generalizable as Enumeration Areas(EAs)are taken from most parts of the country. The larger sample size increased the power of the study and helped to make geographical comparisons. As this was cross-sectional, it ~~does not show a temporal relationship~~. Additionally, potentially important variables such as antenatal care visits, postnatal care, and preceding birth interval were excluded from the study due to their high rate of missing values.

Abbreviations

AOR: Adjusted Odds Ratio, CI: Confidence Interval, CS: Cesarean Section, EAs: Enumeration Areas, DHS: Demography and Health Survey, EMDHS: Ethiopia Mini Demography and Health Survey, HHs: households, ICC: Intraclass Correlation, KR: kids record, SGD: Sustainable Development Goal, U5M: under-five mortality, U5MR: under-five mortality rate

Declaration

Availability of data and materials

The datasets used during the current study are publicly available data from measure DHS program found at www.dhsprogram.com.

Funding

The authors received no specific funding for this work.

Acknowledgment

We acknowledge the DHS program for providing us with the dataset. Elizabeth Korevaar reviewed the English language of the manuscript.

Consent for publication

Not applicable as there are no images or other confidentiality-related issues.

Competing interests

The authors declared that they have no competing interests.

Authors' contributions

LGG, TMT, and ZTT conceived the study, data analysis, drafted the manuscript, and critically reviewed the manuscript. They read and approved the final version of the manuscript.

Reference

1. Croft T, Marshall A, Allen C. Guide to DHS Statistics. Rockville, Maryland, USA: ICF; 2018. 2018.
2. Vidak I. OLDER PEOPLE AND SOCIAL MEDIA USAGE. Strategic Approach to Aging Population: Experiences and Challenges: Sveučilište Josipa Jurja Strossmayera u Osijeku, Ekonomski fakultet u Osijeku. p. 325-40.
3. Woldeamanuel BT, Aga MA. Count models analysis of factors associated with under-five mortality in Ethiopia. Global Pediatric Health. 2021;8:2333794X21989538.
4. Ezbakhe F, Pérez-Foguet A. Child mortality levels and trends. Demographic Research. 2020;43:1263-96.
5. Johnston R. Arsenic and the 2030 Agenda for sustainable development. Arsen Res Glob Sustain-Proc 6th Int Congr Arsen Environ AS. 2016;2016:12-4.
6. Li Z, Hsiao Y, Godwin J, Martin BD, Wakefield J, Clark SJ, et al. Changes in the spatial distribution of the under-five mortality rate: Small-area analysis of 122 DHS surveys in 262 subregions of 35 countries in Africa. PloS one. 2019;14(1):e0210645.
7. Fenta SM, Fenta HM, Ayenew GM. The best statistical model to estimate predictors of under-five mortality in Ethiopia. Journal of Big Data. 2020;7(1):1-14.
8. Worku MG, Teshale AB, Tesema GA. Determinants of under-five mortality in the high mortality regions of Ethiopia: mixed-effect logistic regression analysis. Archives of Public Health. 2021;79(1):1-9.
9. Ayele DG, Zewotir TT, Mwambi HG. Structured additive regression models with spatial correlation to estimate under-five mortality risk factors in Ethiopia. BMC public health. 2015;15(1):1-12.

10. Gebremichael SG, Fenta SM. Under-Five Mortality and Associated Risk Factors in Rural Settings of Ethiopia: Evidences from 2016 Ethiopian Demographic and Health Survey. Advances in Public Health. 2020;2020.
11. Geremew BM, Gelaye KA, Melesse AW, Akalu TY, Baraki AG. Factors Affecting Under-Five Mortality in Ethiopia: A Multilevel Negative Binomial Model. Pediatric Health, Medicine and Therapeutics. 2020;11:525.
12. Ghilagaber G, Antai D, Kandala N-B. Modeling spatial effects on childhood mortality via geo-additive Bayesian discrete-time survival model: A case study from Nigeria. Advanced techniques for modelling maternal and child health in Africa: Springer; 2014. p. 29-48.
13. Singh A, Masquelier B. Continuities and changes in spatial patterns of under-five mortality at the district level in India (1991–2011). International journal of health geographics. 2018;17(1):1-18.
14. Kandala N-B, Mandungu TP, Mbela K, Nzita KP, Kalambayi BB, Kayembe KP, et al. Child mortality in the Democratic Republic of Congo: cross-sectional evidence of the effect of geographic location and prolonged conflict from a national household survey. BMC public health. 2014;14(1):1-9.
15. Anafcheh T, Yaghoubi Doust M, Mojadam M, Mirkazemi R, Khafaie MA. Temporal and spatial distribution of under-five mortality and factors associated with multiple cases of under-five deaths within a family in the rural area of Khuzestan, Southern Iran. Scientific reports. 2018;8(1):1-8.
16. Ayele DG, Zewotir TT. Childhood mortality spatial distribution in Ethiopia. Journal of Applied Statistics. 2016;43(15):2813-28.
17. Dejene T, Girma E. Social determinants of under-five mortality in Ethiopia: Event history analysis using evidence from Ethiopian Demographic and Health Survey (EDHS). 2013.
18. Fikru C, Getnet M, Shaweno T. Proximate determinants of under-five mortality in Ethiopia: using 2016 nationwide survey data. Pediatric health, medicine and therapeutics. 2019;10:169.
19. Zewudie AT, Gelagay AA, Enyew EF. Determinants of under-five child mortality in Ethiopia: analysis using Ethiopian demographic health survey, 2016. International Journal of Pediatrics. 2020;2020.
20. Abir T, Agho KE, Page AN, Milton AH, Dibley MJ. Risk factors for under-5 mortality: evidence from Bangladesh Demographic and Health Survey, 2004–2011. BMJ open. 2015;5(8):e006722.
21. Pretorius CE, Asare H, Genuneit J, Kruger HS, Ricci C. Impact of breastfeeding on mortality in sub-Saharan Africa: a systematic review, meta-analysis, and cost-evaluation. European Journal of Pediatrics. 2020;179(8):1213-25.
22. Ethiopian Public Health Institute - EPHI, Federal Ministry of Health - FMoH, ICF. Ethiopia Mini Demographic and Health Survey 2019. Addis Ababa, Ethiopia: EPHI/FMoH/ICF; 2021.
23. Fotheringham ASRP. The SAGE handbook of spatial analysis. Los Angeles; London: SAGE Publications; 2009.
24. Bhunia G, Shit P, Maiti R. Comparison of GIS-based interpolation methods for spatial distribution of soil organic carbon (SOC). J Saudi Soc Agric Sci 17: 114–126. 2018.
25. Kulldorff M. A spatial scan statistic. Communications in Statistics-Theory and methods. 1997;26(6):1481-96.
26. Sommet N, Morselli D. Keep calm and learn multilevel logistic modeling: A simplified three-step procedure using stata, R, Mplus, and SPSS. International Review of Social Psychology. 2017;30:203-18.
27. GeoPortal I. OpenAfrica shapfile 2019 [Available from: https://open.africa/dataset/africa-shapefiles.
28. Azuike E, Onyemachi P, Amah C, Okafor K, Anene J. Determinants of under-five mortality in South-Eastern Nigeria. J Community Med Public Health Care. 2019;6:049.
29. Aheto JMK. Predictive model and determinants of under-five child mortality: evidence from the 2014 Ghana demographic and health survey. BMC public health. 2019;19(1):1-10.
30. Julihn A, Soares FC, Hammarfjord U, Hjern A, Dahllöf G. Birth order is associated with caries development in young children: a register-based cohort study. BMC Public Health. 2020;20(1):1-8.

31. Ahmed AH. The International Code of Marketing of Breast-milk Substitutes: Update on the Global Implementation. Journal of Human Lactation. 2020;36(4):803-7.
32. WHO U. Global nutrition targets 2025: breastfeeding policy brief (WHO/NMH/NHD14. 7). Geneva: World Health Organization. 2014.
33. Elster N. Less is more: the risks of multiple births. Fertility and Sterility. 2000;74(4):617-23.
34. Goyal S, Kortsmit K, Cox S, D'Angelo DV, Romero L, Henderson ZT, et al. Prevalence of Home Births and Associated Risk Profile and Maternal Characteristics, 2016–2018. Obstetrics & Gynecology. 2020;136(6):1195-203.
35. Dessalegn M, Ayele M, Hailu Y, Addisu G, Abebe S, Solomon H, et al. Gender inequality and the sexual and reproductive health status of young and older women in the Afar region of Ethiopia. International Journal of Environmental Research and Public Health. 2020;17(12):4592.
36. Oladeji O, Oladeji B, Omer MD, Farah AE, Ameda IM, Gera R, et al. Exploring opportunities to enhance effectiveness of mobile health and nutrition strategy for providing health and nutrition services amongst pastoralists in Somali region, Ethiopia. African Journal of Primary Health Care & Family Medicine. 2021;13(1):1-7.

List of Figures

Figure 1: conceptual framework of under-five mortality in Ethiopia 2019(27).

Figure 2: Spatial autocorrelation of under-five mortality in Ethiopia 2019(27).

Figure 3: Hotspot analysis of under-five mortality in Ethiopia 2019(27).

Figure 4: Empirical Bayesian Kriging prediction map of under-five mortality in Ethiopia 2019(27).

Figure 5: Spatial scan statistics of under-five mortality in Ethiopia 2019(27).

Figure 6: residual diagnostic plot for the final model of under-five mortality in Ethiopia 2019.

Table 1 Sociodemographic, maternal, and child health factors

Sociodemographic, Maternal and child health factors		Frequency	Percentage
Age of the mother at first birth	Less than 18	3,024	53.10%
	19 to 34	2,650	46.53%
	35 and more	21	0.37%

Sex of child	Male	2,942	51.66%
	Female	2,753	48.34%
Current Contraceptive utilization	Not used	3,875	68.04%
	Used	1,820	31.96%
Multiple births	Singleton	5,528	97.07%
	Multiple	167	2.93%
Birth order	1st order	1,228	21.56%
	2nd and 3rd order	1,883	33.06%
	4th and 5th order	1,298	22.79%
	6th order +	1,286	22.58%
Place where the mother delivered	Home delivery	2,937	51.57%
	Facility delivery	2,758	48.43%
Delivery by cesarean section	No	5,351	93.96%
	Yes	344	6.04%
Months of Breastfeeding	Less than 6 months	918	16.12%
	6 months and more	4,777	83.88%
Sex of household head	Male	4,561	80.09%
	Female	1,134	19.91%
Age of household head (years?)	Less than 35	2,479	43.53%
	35 to 50	2,596	45.58%
	50 and more	620	10.89%
Marital Status	Married	5,346	93.87%
	Unmarried	349	6.13%
Highest educational level	No education	3,135	55.05%
	Primary	1,802	31.64%
	Secondary	467	8.20%
	Higher	291	5.11%
Wealth index	Poor	2,940	51.62%
	Middle	797	13.99%

	Rich	1,958	34.38%
Water source	Improved	3,660	64.27%
	Unimproved	2,035	35.73%
Time to fetch water	More than 30 min	1,765	30.99%
	Less than 30 min	2,896	50.85%
	On premises	1,034	18.16%
Type of latrine used	Improved	1,164	20.44%
	Not improved	4,531	79.56%
Household has electricity	No	4,073	71.52%
	Yes	1,622	28.48%
Cooking fuel in use	Clean fuel	432	7.59%
	Solid fuel	5,263	92.41%
Type of floor of the house	Earth/sand/dung	4,384	76.98%
	Wood	49	0.86%
	Cement	799	14.03%
	Carpet	463	8.13 %
Type of the house wall	Cane/Palm/Trunk/Reed	1,261	22.14%
	Bamboo/wood	3,531	62.00%
	Cement/stone with lime/bricks/covered adobe	903	15.86%
Type of the house roof	Thatch	1,488	26.13%
	Palm/bam	995	17.47%
	Cement	198	3.48%
	Corrugated iron	3,014	52.92 %

Table 2 Community-level variable

Community level variable		frequency	Percentage
Place of residence	Urban	1,306	22.93%
	Rural	4,389	77.07%
Community place of delivery	Home delivery	3,457	60.70%
	Facility delivery	2,238	39.30%
Community toilet utilization	Improved	2,520	44.25%
	Not improved	3,175	55.75%
Community wealth status	Poor	2,776	48.74%
	Rich	2,919	51.26%
Community media exposure	Not exposed to media	3,391	59.54%
	Exposed to media	2,304	40.46%

Table 3 Under-five mortality among predictors

Characteristics		Is the child alive?		total
		Yes 5357(94.06%)	No338(5.94%)	
Is child twin?	Yes	123(2.16%)	44(0.77%)	167(2.93%)
	No	5234(91.9%)	294(5.16%)	5,528(97.06%)
Region	Tigray	436(7.65%)	14(0.24%)	450(7.90%)
	Afar	616(10.81%)	35(0.612%)	651(11.43%)
	Amhara	478(8.39%)	23(0.40%)	501(8.79%)
	Oromia	674(11.83%)	41(0.72%)	715(12.55%)
	Somali	580(10.18%)	57(1.00%)	637(11.18%)

	Benishangul	477(8.37%)	45(0.79%)	522(9.17%)
	SNNPR	631(11.08%)	26(0.46%)	657(11.54%)
	Gambella	409(7.18%)	35(0.61%)	444(7.79%)
	Harari	408(7.16%)	29(0.51%)	437(7.67%)
	Addis Ababa	281(4.93%)	6(0.11%)	287(5.04%)
	Dire Dawa	367(6.44%)	27(0.47%)	394(6.92%)
Breastfeeding	Less than 6 months	773(13.57%)	145(2.55%)	918(16.12%)
	6 months and more	4,584(80.49%)	193(3.39%)	4,777(83.88%)
Birth order	1st order	1,142(20.05%)	86(1.52%)	1,228(21.56%)
	2-3rd order	1,793(31.48%)	90(1.56%)	1,883(33.06%)
	4-5th order	1,231(21.62%)	67(1.18%)	1,298(22.79%)
	6th + order	1,191(20.91%)	95(1.67%)	1,286(22.58%)
Place of delivery	Home delivery	2,733(47.99%)	204(3.58%)	2,937(51.57%)
	Facility delivery	2,624(46.06%)	134(2.35%)	2,758(48.43%)

Table 4 Multivariable multilevel logistic regression analysis of both individual and community-level factors associated with under-five mortality in Ethiopia, EMDHS 2019.

Variable	Null model	Model2 AOR (95%CI)	Model3 AOR(95%CI)	Model4 AOR(95%CI)
Education				
No education		1		1
Primary		1.14 (0.83,1.56)		1.14 (0.83,1.58)
Secondary		0. .59 (0.31, 1.13)		0.58(0.30,1.12)
Higher		0. .65(0. .28, 1.55)		0.60(0.25,1.44)
Household members		0.922(0. 85, 1.01)		0.92(0.83,0.99) *
Number of under-five children		0.17 (0.14, 0 .22)		0.17(0.14, 0.21) ***
Sex				
Male		1		1
female		0 .83(0 .64, 1.07)		0.84(0.64,1.09)
Mode of delivery by C/S				
No		1		1
Yes		1.36 (0. 79, 2.35)		1.37(0.79,2.39)
Preceding birth order				
1st		1		1
2-3rd		1.32(0.92, 1.91)		1.34(0.93,1.94)
4-5th		1.52(0.97, 2.39)		1.51(0.96,2.38)
6+		2.44(1.48, 4.02)		2.46(1.49,4.06) ***
Time to water				
>30min		1		1
<30min		0.77(0.56, 1.04)		0.89(0.64,1.23)
On-premises		0.66(0.38, 1.14)		0.76(0.43,1.34)
Cooking fuel				
Clean fuel		1		1

Solid fuel		1.27(0.62, 2.58)		0.93(0.45,1.94)
Age at 1st birth				
19-34		1		1
<=18		0.91(0.70, 1.20)		0.90(0.68,1.18)
>=35		2.58(0.55, 12.01)		2.72(0.55,13.59)
Breastfeeding				
< 6 months		1		1
>= 6 months		5.09(3.85, 6.72)		5.05(3.82,6.67) ***
Place of delivery				
Home delivery		1		1
Facility delivery		0.54 (0.40,0.75)		0.57(0.41,0.81) **
Main floor				
earth/sand/dung		1		1
Wood		1.71(0.53, 5.52)		2.14(0.66,6.95)
Cement		0.89(0.50, 1.59)		0.86(0.48,1.58)
Carpet		1.56(0.85, 2.86)		1.28(0.67,2.46)
Main wall				
bamboo/wood		1		1
cane/palm/trunks/re ed		1.42(0 .97, 2.07)		1.06(0.70,1.58)
cement/stone		1.53(0.89,2.61)		1.34(0.76,2.34)
Main roof				
Corrugated iron		1		
thatch		1.25(0.87,1.79)		1.14(0.77,1.67)
palm/bamboo		0.71(0.44,1.15)		0.57(0.34,0.96)*
calamine/cement		1.14(0.54,2.44)		1.04(0.47,2.29)
Contraceptive utilization				
No		1		1
Yes		0.72(0.53,0.98)		0.82(0.60,1.13)

Marital status				
Currently Married		1		1
Currently Unmarried		1.19(0.74,1.92)		1.22(0.75,1.97)
Multiple Twin child				
No		1		1
Yes		15.63(9.33,26.20)		14.39(8.51,24.33)***
Region				
Tigray			1	1
Afar			1.60(0.76,3.41)	2.55(1.10,5.85)*
Amhara			1.47(0.68,3.19)	0.97(0.44,2.15)
Oromia			1.73(0.84,3.55)	1.52(0.73,3.15)
Somali			2.86(1.39,5.89)	3.46(1.59,7.55)**
Benishangul gumuz			2.74(1.32,5.68)	1.95(0.91,4.20)
SNNPR			1.17(.55,2.47)	0.94(0.44,2.03)
Gambella			2.71(1.29,5.69)	1.94(0.90,4.21)
Harari			2.37(1.12,5.04)	1.95(0.87,4.36)
Addis Ababa			0.71(0.25,2.03)	0.64(0.21,2.02)
Dire Dawa			2.18(1.01,4.70)	2.02(0.91,4.50)
Community delivery place				
Home delivery			1	1
Facility delivery			0.75(0.52, 1.09)	0.94(0.62,1.43)
Community media				
Not exposed			1	1
exposed			1.09(0.75,1.58)	0.94(0.64,1.40)
Intercept	0.49(0.30,0.81)	0.22(0.08,0.63)	0.35(0.19,0.64)	0.16(0.04,0.57)
ICC	0.130	0.036	0.091	0.025
Log-likelihood	-1263.56	-918.15	-1247.40	-903.72

*** P-value<0.001, ** p-value<0.01, * p-value<0.05

433

 Click here to access/download;Figure;Figure 1.tif

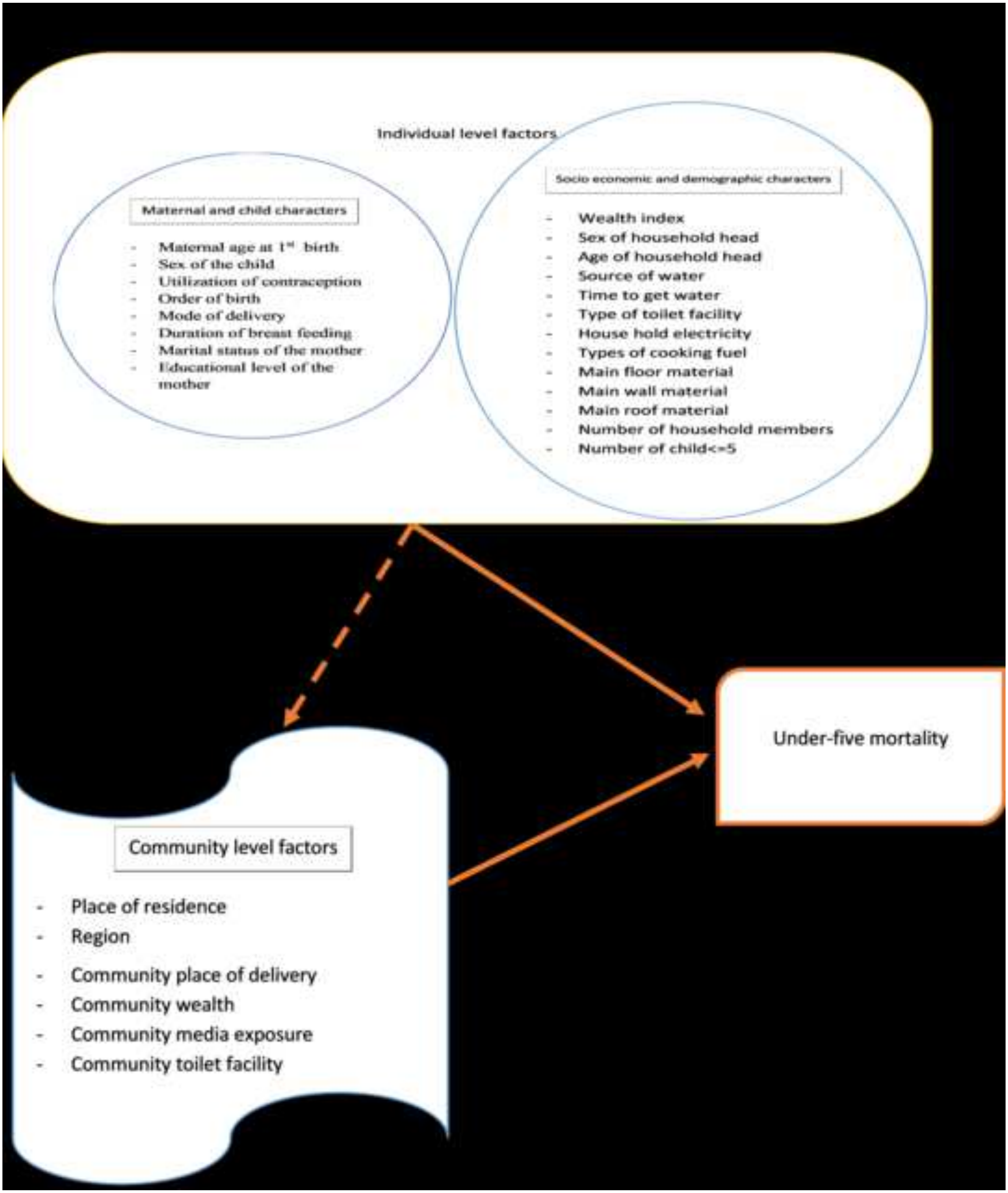

Figure

Click here to access/download;Figure;Figure 2.tif

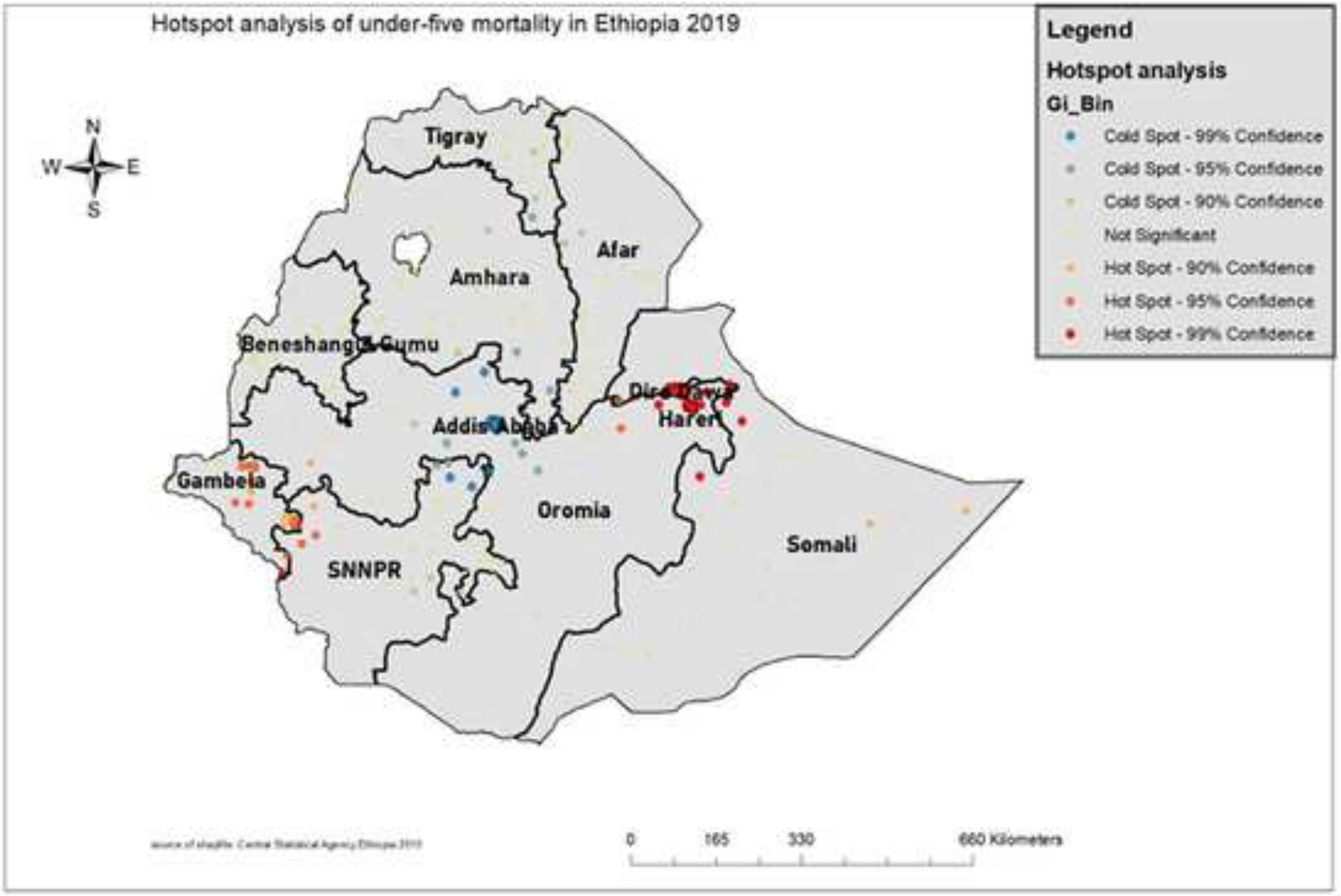

Figure

Click here to access/download;Figure;Figure 3.tif

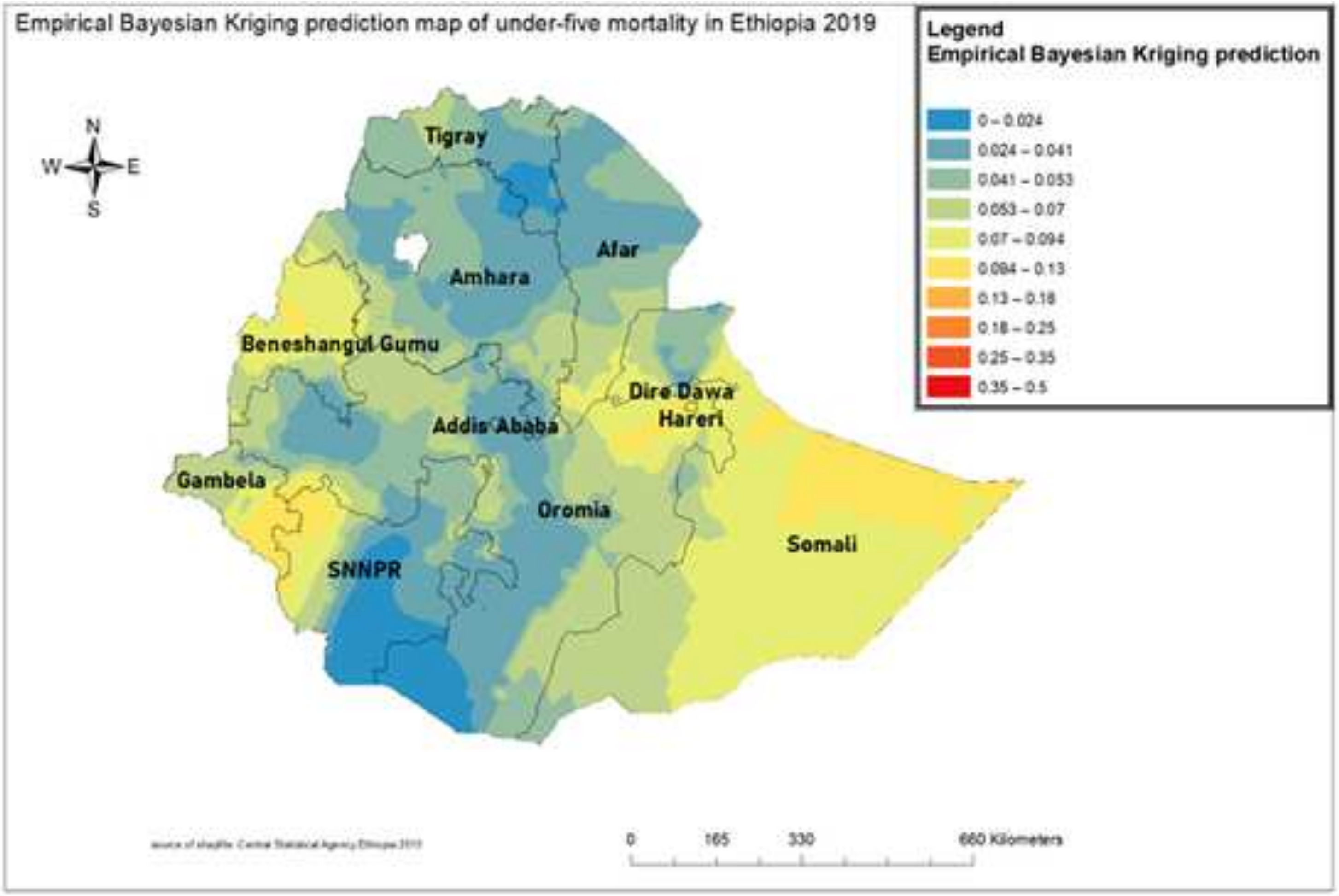

Figure
Click here to access/download;Figure;Figure 4.tif

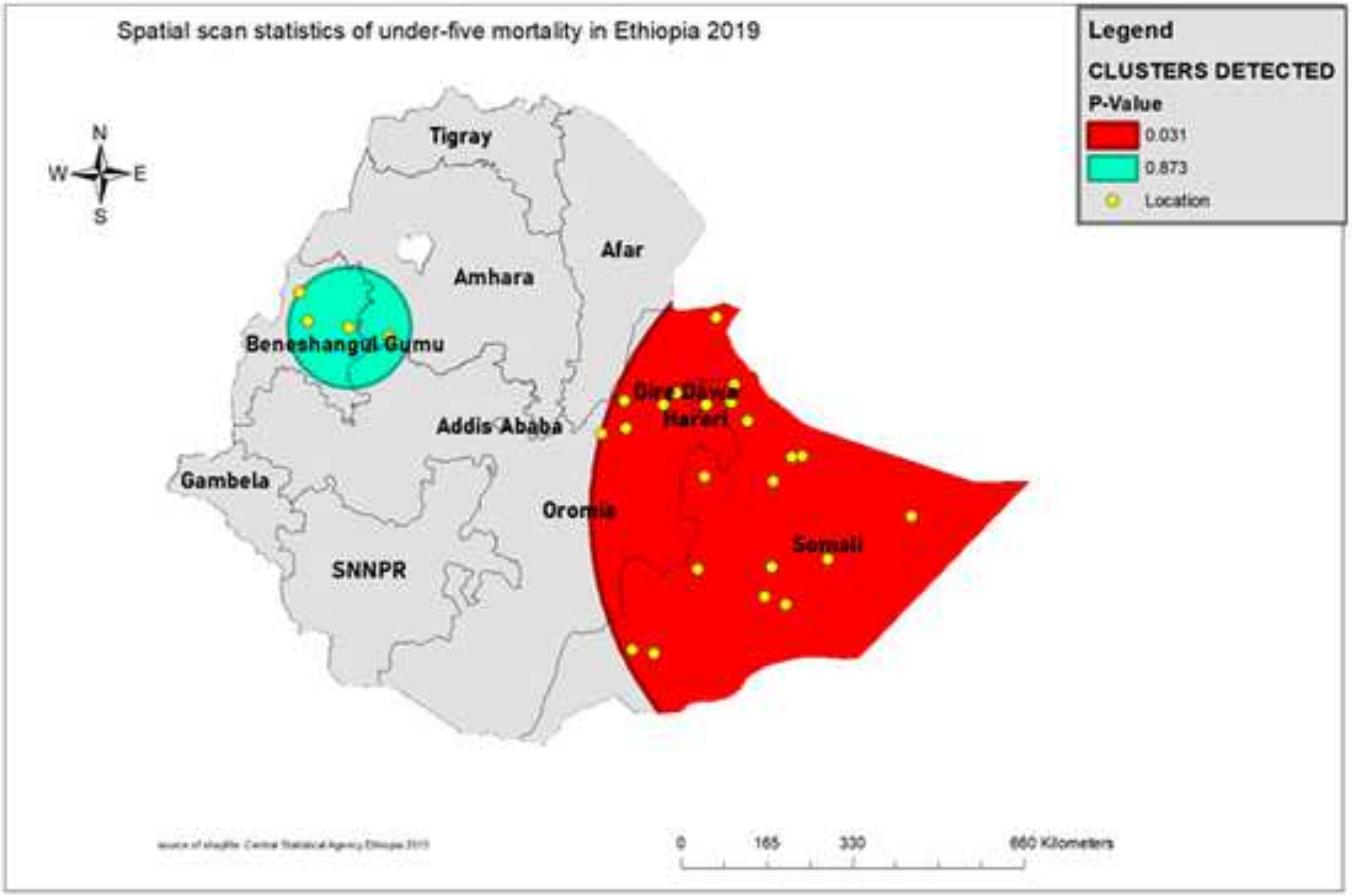

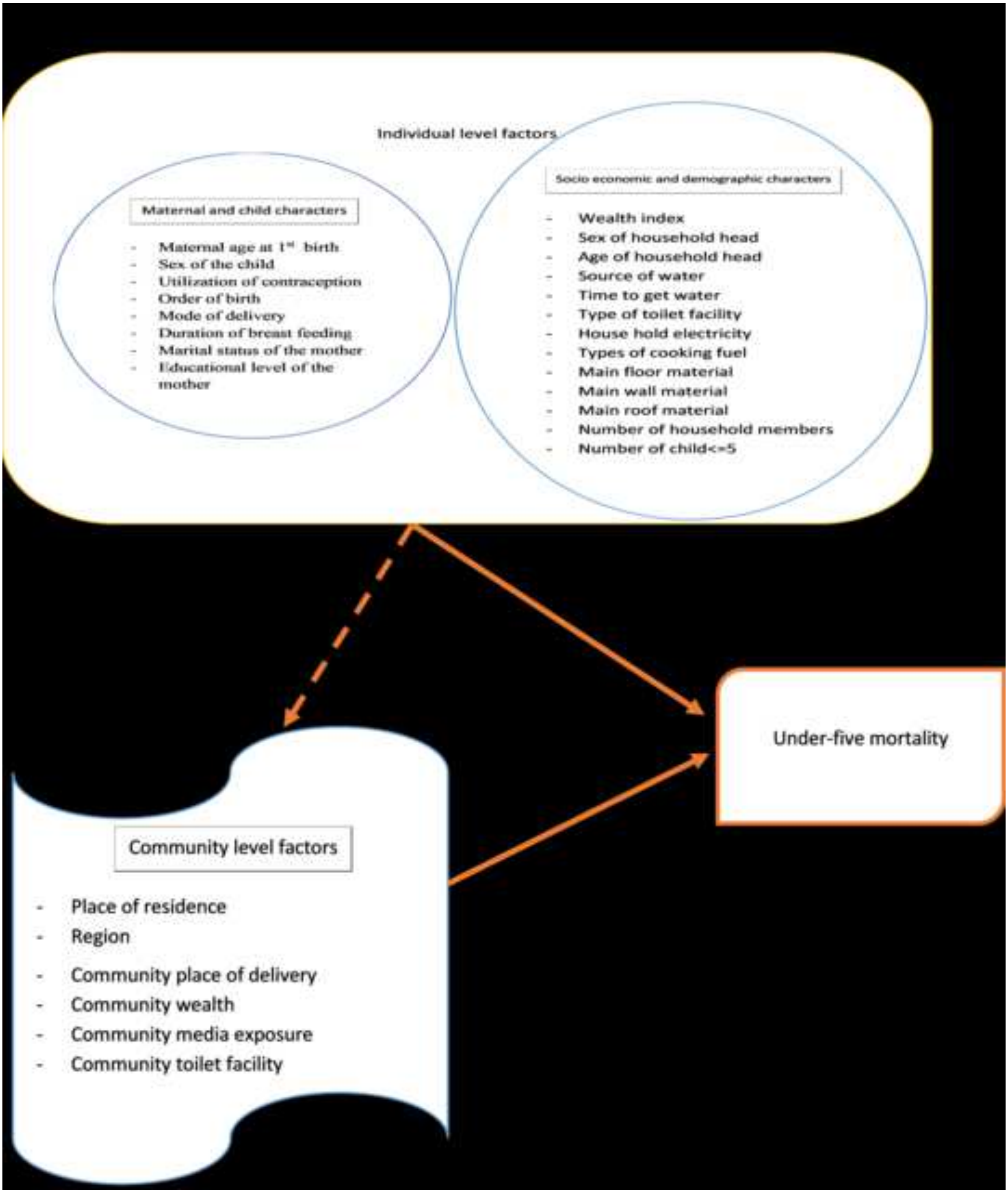
Individual level factors
Maternal and child characters
- Maternal age at 1st birth
- Sex of the child
- Utilization of contraception
- Order of birth
- Mode of delivery
- Duration of breast feeding
- Marital status of the mother
- Educational level of the mother
Socio economic and demographic characters
- Wealth index
- Sex of household head
- Age of household head
- Source of water
- Time to get water
- Type of toilet facility
- House hold electricity
- Types of cooking fuel
- Main floor material
- Main wall material
- Main roof material
- Number of household members
- Number of child<=5
Under-five mortality
Community level factors
- Place of residence
- Region
- Community place of delivery
- Community wealth
- Community media exposure
- Community toilet facility

Figure

Click here to access/download;Figure;Figure 2 (2).tif

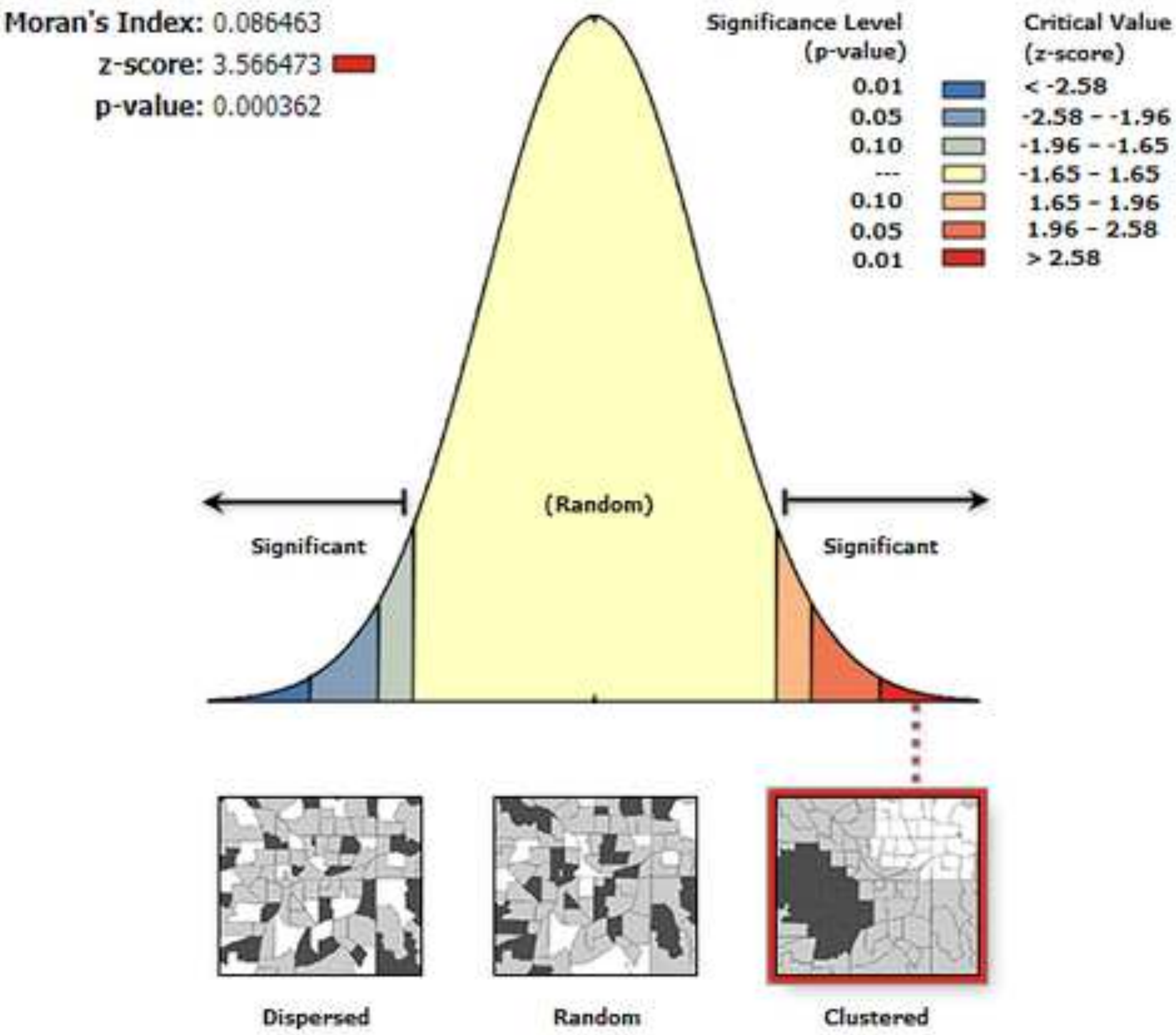

Figure

Click here to access/download;Figure;Figure 3 (2).tif

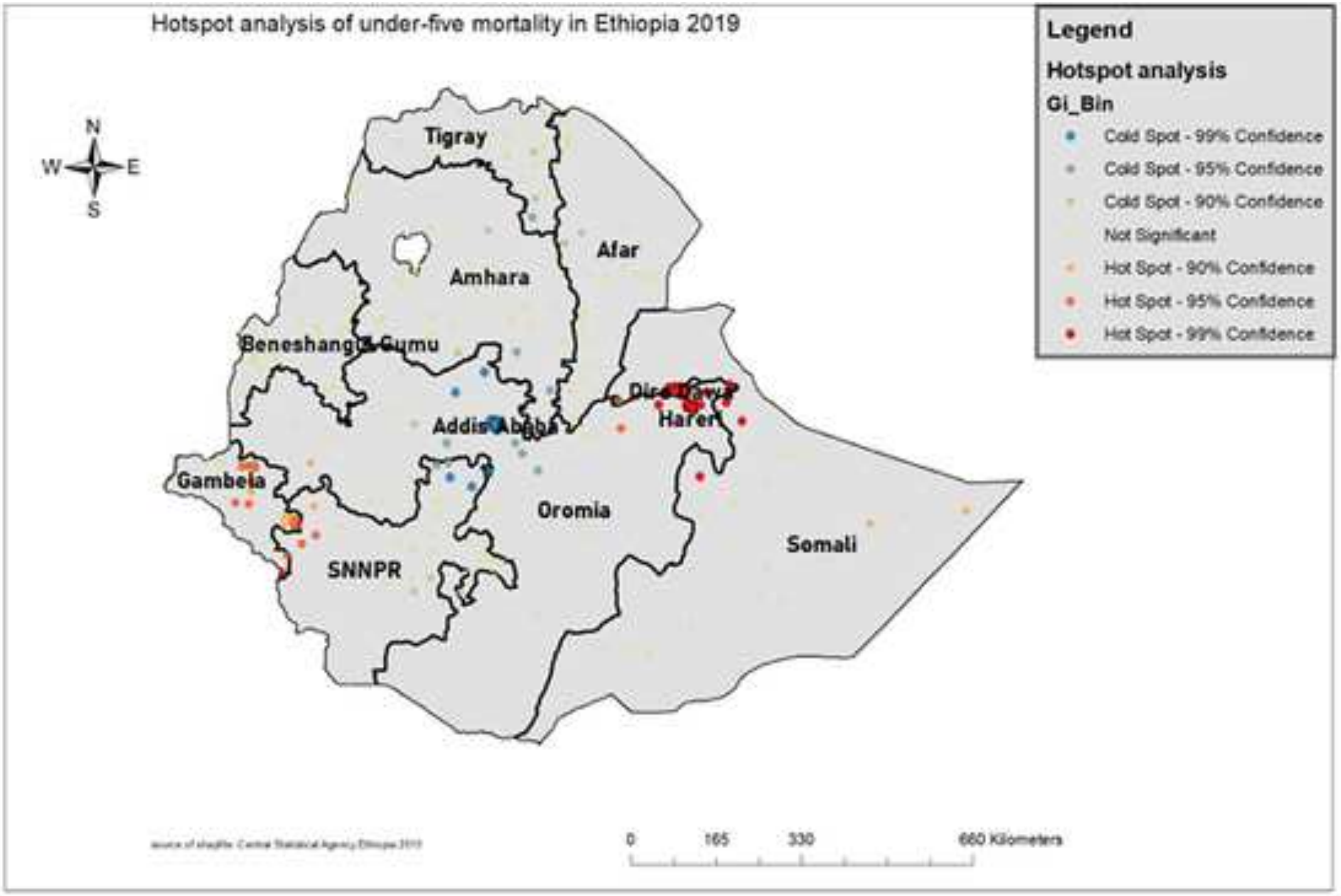

Figure
Click here to access/download;Figure;Figure 4 (2).tif

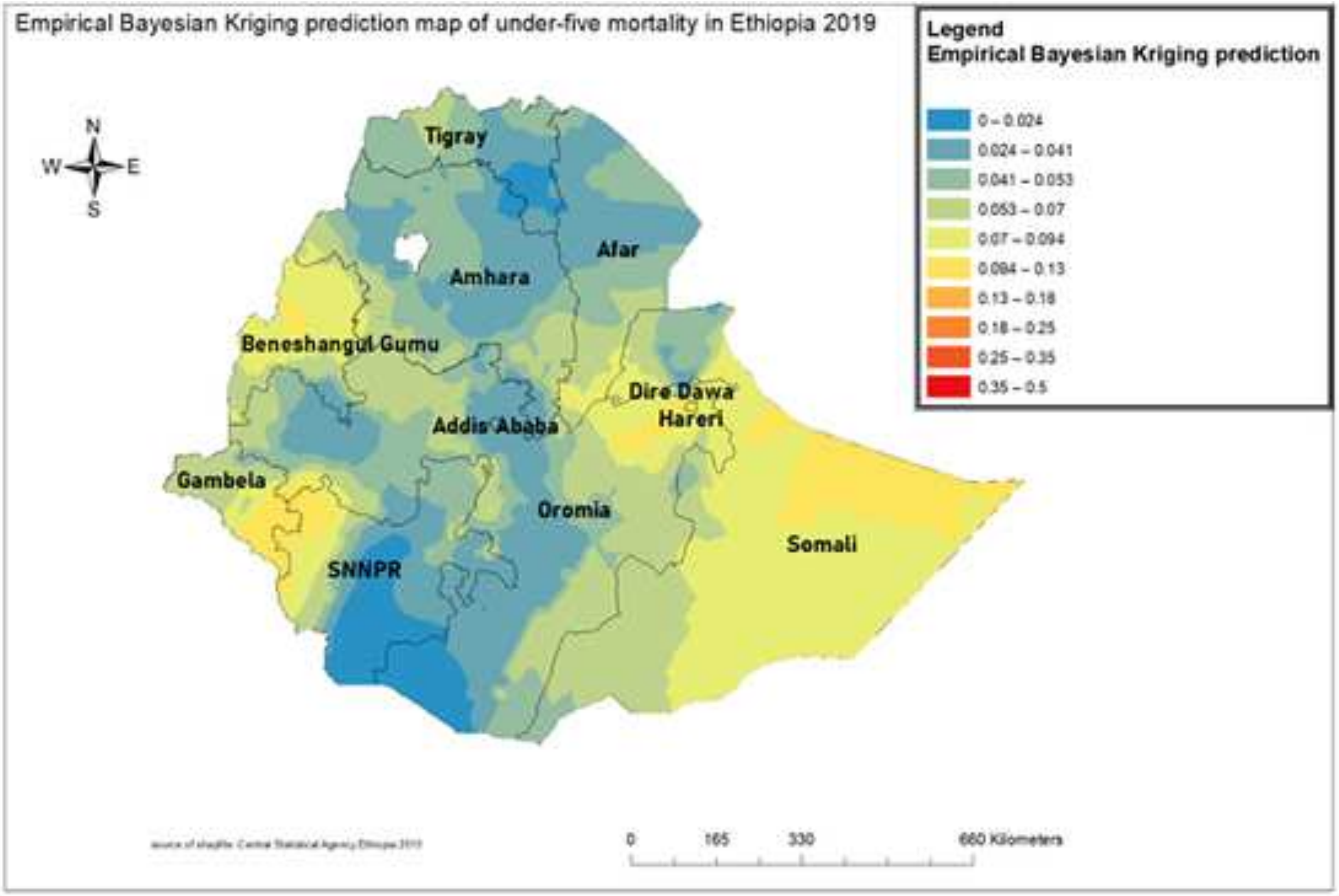

Figure

Click here to access/download;Figure;Figure 5 (2).tif

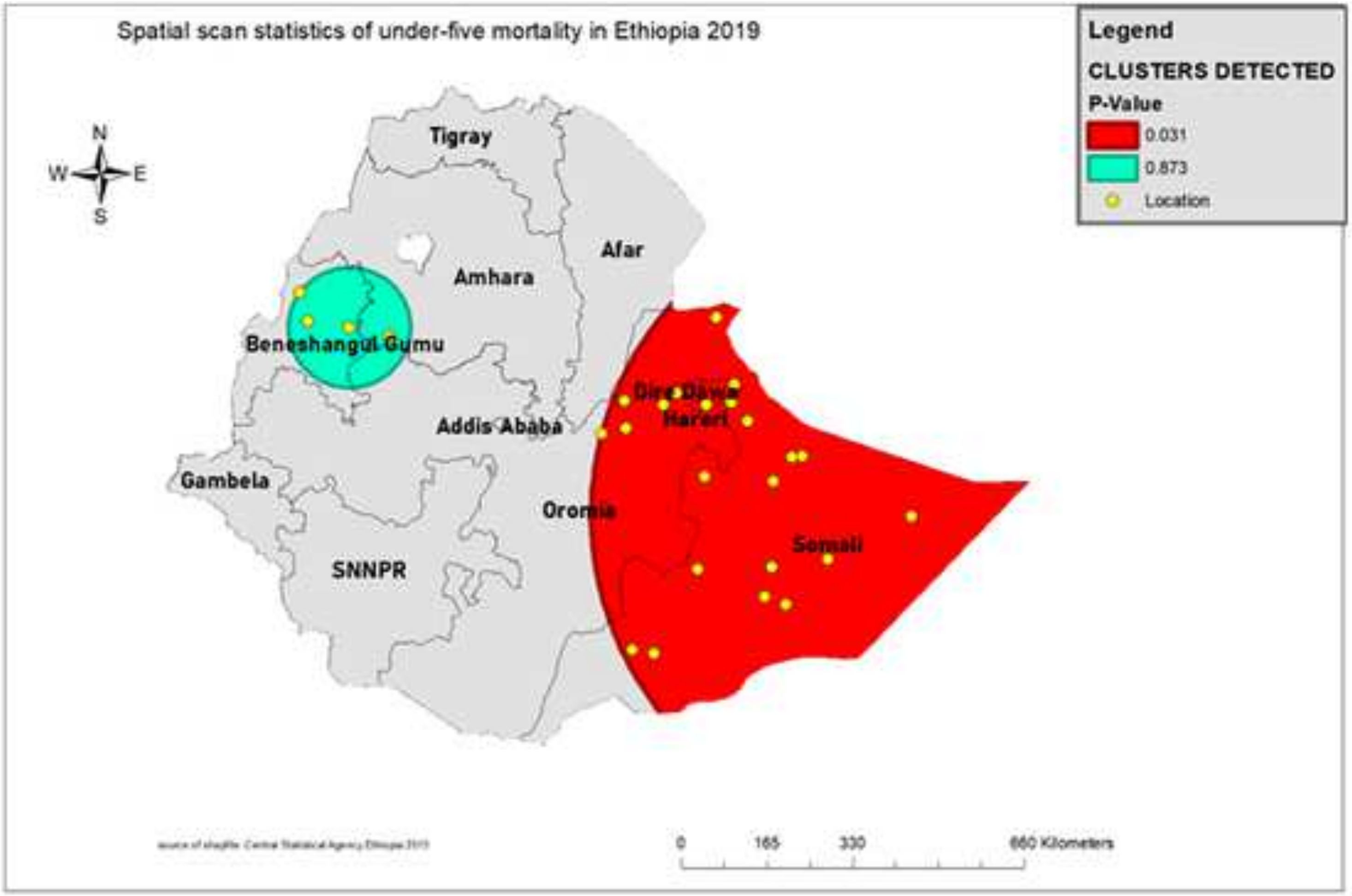

 Click here to access/download;Figure;Figure 6 (2).tif

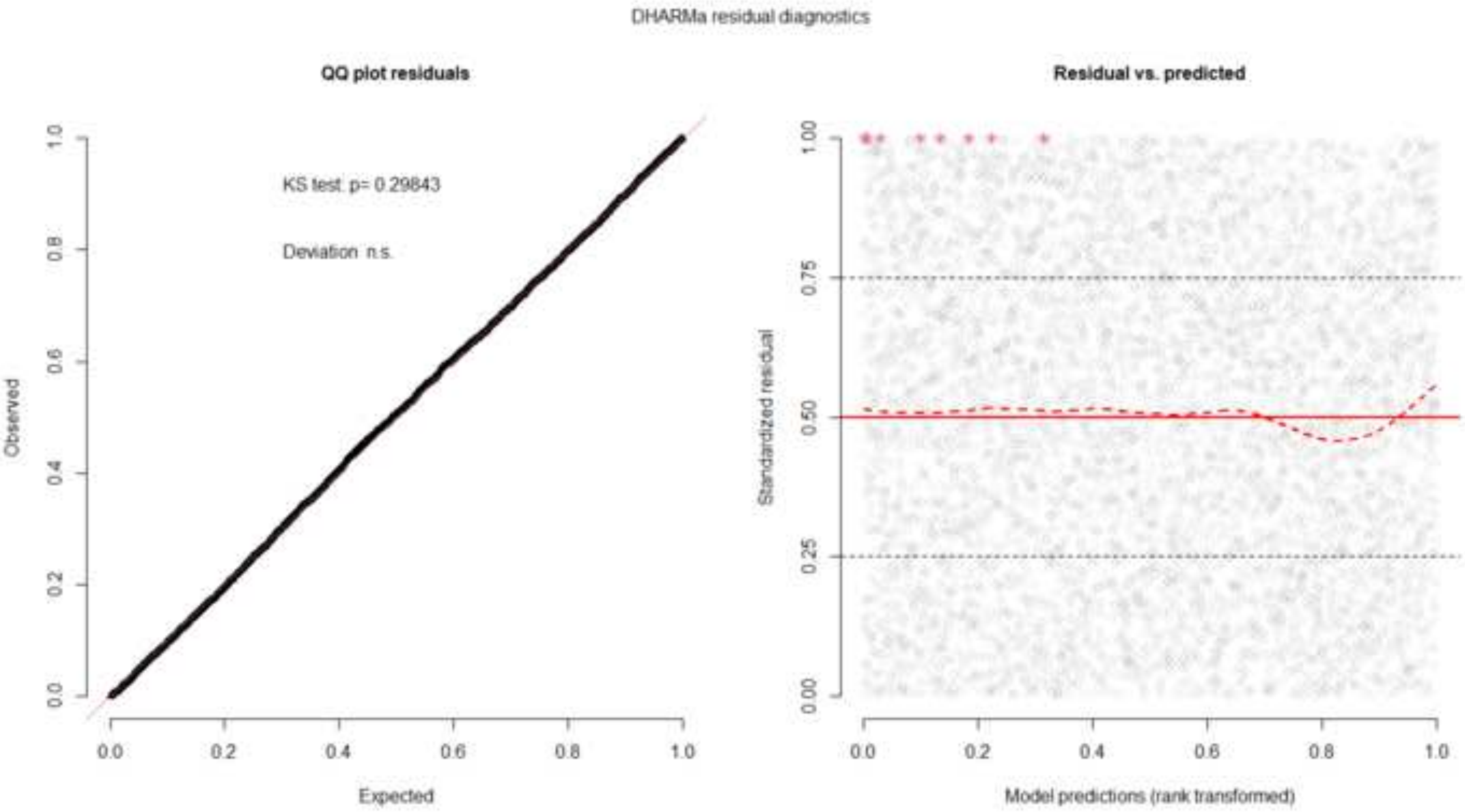

Geographic variation and factors associated with under-five mortality in Ethiopia. A spatial and multilevel analysis of Ethiopian Mini Demographic and Health Surveys 2019.

Zemenu Tadesse Tessema [1,2*], ~~Elizabeth Korevaar[1],~~ Tsion Mulat Tebeje[3], Lewi Goytom Gebrehewet[2]

[1]Department of Epidemiology and Preventive Medicine, School of Public Health and Preventive Medicine, Monash University, Melbourne, Australia

[2] Department of Epidemiology and Biostatistics, Institute of Public Health, College of Medicine and Health Sciences, University of Gondar, Gondar, Ethiopia

[3] Epidemiology and Biostatistics Unit, School of public health, Dilla University, Dilla, Ethiopia

E-mail: ZTT zemenut1979@gmail.com

~~**EK:** elizabeth.korevaar1@monash.edu~~

TMT yemarina12@gmail.com

LGG lewigt369@gmail.com

***= Corresponding author**

Abstract

Background~~:~~

The distribution of under-five mortality (U5M) worldwide is uneven and the burden is higher in Sub-Saharan African countries, which account for more than 53% of the global under-five mortality. In Ethiopia, though U5M ~~shows a substantial decrease from~~decreased substantially between 1990 ~~to~~and 2019, it ~~is still~~remains excessively high and unevenly distributed. Therefore, this study aimed to assess geographic variation and factors associated with under-five mortality (U5M) in Ethiopia.

Methods

We sourced data from the most recent nationally representative 2019 Ethiopian Mini-Demographic and Health Survey for this study. A sample size of 5,695 ~~live~~total births was considered ~~for this study.~~. Descriptive ~~and~~, analytical ~~Analysis was~~analysis and spatial analysis were conducted ~~by~~ using STATA version 16. Both multilevel and spatial analyses were employed to ascertain the factors associated with ~~under-five mortality~~U5M in Ethiopia.

Results

The ~~under-five mortality is~~U5M was 5.9% with ~~its~~a 95% CI 5.4% to 6.6%. Based on the multivariable multilevel logistic regression model ~~result;~~results, the following characteristics were associated with under-five mortality: family size (AOR=0.~~09~~92, 95%CI ~~:(~~: 0.84, 0.99~~)),~~), number of under-five children in the family (AOR=0.17, 95%CI: 0.14, 0.21), multiple birth (AOR=14.4, 95%CI: ~~(~~8.5, 24.3~~)), Children~~), children who were breastfed for less than 6 months (AOR=5.04, 95%CI: ~~(~~3.81, 6.67~~)), People~~), people whose main roof is palm (AOR=0.57, 95%CI: 0.34, 0.96), ~~Under~~under-five children who are ~~at birth order~~ the sixth or more child to be born (AOR=2.46, 95%CI: ~~(~~1.49, 4.06~~)),~~), institutional delivery (AOR=0.57, 95%CI: ~~(~~0.41, 0.81~~)), being~~), resident of Somali and Afar region (AOR=3.46, 95%CI: ~~(~~1.58, 7.55~~))~~) and (AOR=2.54, 95%CI: ~~(~~1.10, 5.85~~))~~), respectively ~~were factors associated with under-five mortality. A spatial~~. Spatial analysis ~~result~~ revealed that hot spot areas of under-five mortality were located in the ~~Somali region and~~ Dire Dawa~~.~~ and Somali regions.

Conclusion:

Under-five mortality in Ethiopia is ~~59 per 1000 live births which are~~ high and unacceptable ~~as~~when compared to the 2030 ~~agenda for~~ sustainable development ~~aimed at~~ target, which aims for 25 per 1000 live births. Breastfeeding for less than 6-~~month~~months, twin ~~birth~~births, institutional delivery and high-risk areas of under-five mortality (Somali and dire Dawa) ~~were~~are modifiable risk factors. Therefore, maternal education on the ~~advantage~~advantages of breastfeeding and institutional delivery is highly recommended. Women who deliver twins should be given special attention. An effective strategy should be designed for intervention ~~for hot spot areas of~~in under-five mortality ~~that is~~hot spot areas such as Somali and Dire Dawa.

Keywords: Under-five mortality, factors, spatial distribution, multilevel, Ethiopia

Formatted: Font: Bold

Background

Under-five mortality is defined as deaths reported at ages 0 to 59 months and ~~it contains~~includes neonatal, post-neonatal, and child deaths (1). Under-five mortality rate (U5MR) is the probability of a child dying before celebrating ~~the~~their fifth birthday(2). It is a significant indicator of the socioeconomic, health, and environmental conditions, and the national development of health equity and access(3).

Globally, in 1990, the number of under-five deaths ~~were~~was 12,494,000 (93 deaths per 1000 live births). ~~By showing~~Following an average annual decline of 3.1%, ~~in~~by 2019 it was significantly reduced to 5,189,000 deaths (38 deaths per 1000 live births) ~~with a~~representing 58% reduction(4). The 2030 ~~agenda for~~ sustainable development ~~is aimed at ending~~(SDG?) aims to end the preventable deaths of newborns and children under 5 years of age, with all countries aiming to reduce under-5 mortality to ~~make it~~ at ~~least less than 25~~least25 per 1000 live births or lower(5).

~~The distribution of U5MR worldwide is uneven and has wider disparity and the global burden of U5MR is concentrated at two sustainable development goals (SDG) regions, Sub-Saharan Africa and Central and Southern Asia with more than 80% of U5MR accounted for(6)~~The distribution of U5MR worldwide is uneven and the global burden of U5MR is concentrated in two regions, Sub-Saharan Africa and Central and Southern Asia, which accounts for more than 80% of U5MR(6).

The burden is even higher in Sub-Saharan African countries with 74 deaths per 1000 live births, which accounts for more than 53% of the global under-five mortality(7).

In Ethiopia, U5MR ~~shows a substantial decrease~~has substantially decreased from 200 per 1000 live births in 1990 to ~~55~~49 per 1000 live births in ~~2019~~2020 but ~~still couldn't achieve the~~ is not close to Sustainable Development(SDG ~~aim)~~)(4)~~, additionally~~. Additionally, different studies conducted in Ethiopia revealed several ~~Socioeconomic~~socioeconomic, demographic, and geographic or spatial variation ~~is considerable, and~~. For example, Afar, Somali, and Benishangul Gumuz are the three high-risk regions of Ethiopia for U5MR(8).

Different studies ~~conducted on~~examining U5M previously showed that source of water(9), multiple births (7, 8), region (10, 11), age and sex of household head (11), maternal education status ~~(12, 13)~~(12, 13), place of delivery(10, 11, 14), maternal age(15-17), sex of the child(9, 14, 17), residence (18, 19), family size ~~(10, 16)~~(10, 16), number of children under five~~(19, 20)~~(19, 20), birth order ~~(10, 16)~~(10, 16), and breastfeeding status (19, 21) have a significant association with under-five mortality.

~~Though~~Although U5M has been declining it ~~is still~~remains high and ~~is taking~~takes the lives of many children, ~~and~~ no research on U5M has been made ~~on~~until recently, with the ~~recent survey data,~~ Ethiopian mini–Demographic Health Survey (EMDHS) 2019. ~~Therefore,~~Research is required to ~~make different~~inform policies and interventions ~~of U5M~~for the prevention of U5M with the efficient allocation of scarce resources ~~according~~ to ~~spatial variability~~priority areas based ~~on~~. This study aimed to assess the geographic variation and factors associated with under-five mortality in Ethiopia. The results of our ~~findings and to achieve~~study will assist in achieving the SDG ~~by understanding the~~, by providing an understand of the current burden of U5M in Ethiopia, and its determinants, in addition to identifying areas and factors with the highest burden of U5M in Ethiopia ~~is helpful to show the current burden of U5M, hence this study is aimed at assessing the geographic variation and factors associated with Under-five mortality in Ethiopia.~~ .

Method

Data Source

Our source was the 2019 EMDHS~~ is~~, the second mini demographic health survey (DHS) conducted in Ethiopia~~, which is ~~ (a land-locked country located in the Horn of Africa that lies between the ~~30 N~~30N and 150N Latitude or ~~330 E~~330E and ~~480 E~~480E Longitude)(22). Data collection was conducted from March 21, 2019, to June 28, 2019, from the nine regions (Tigray, Afar, Amhara, Oromia, Somali, Benishangul Gumuz, ~~SNNPR,~~Southern nation nationalities and People region (SNNPR), Harari, and Gambella) and two administrative cities (Addis Ababa and Dire Dawa). The study design was a population-based cross-sectional study. ~~Kids Record (KR) format was the data of interest to conduct the study. ~~A frame of all census Enumeration areas (EAs) was used as a sampling frame for the 2019 EMDHS. 149,093 EAs were created which ~~covers~~cover an average of 131 Households (HHS). A two-stage stratified cluster sampling technique was employed and each region was stratified into urban and rural areas, yielding 21 sampling strata~~. Enumeration areas (EAs)~~ were selected independently in each stratum. In the first stage, 305 clusters (93 urban and 212 rural) were selected with probability proportional to EAs size and with independent selection in each sampling stratum. In the second stage, a fixed number of 30 households per cluster were selected. Finally, women aged 15–49 in ~~9150 (2790~~9,150 (2,790 urban and ~~6360~~6,360 rural) households from 305 clusters were selected. The whole procedure of sampling is found in the full 2019 EMDHS report(22).

Study variables

The ~~response~~outcome variable was under-five mortality status, which was categorized as (child alive: Yes= 0 and No = 1). The age was recorded in months. ~~Independent~~The independent variables ~~were~~, classified as individual~~ -~~-level and community~~ -~~-level predictors~~ (Place~~, were place of residence, ~~Region, Community~~region, community place of delivery, ~~Community~~community wealth, ~~Community~~community media exposure, and ~~Community~~community toilet facility~~), some of this community level predictors are driven from individual level predictors~~. The individual-level predictors were further categorized as socio-demographic and economic factors ~~(Educational~~like the educational level of the mother, ~~Sex~~sex of the household head, age of household head, ~~Number~~number of household members, ~~Number~~number of ~~a child~~childred under the age of five, ~~Marital~~marital status of the mother, ~~Source~~source of water, ~~Time~~time to get water, ~~Type~~type of toilet facility, ~~Household~~household electricity, ~~Types~~types of cooking fuel, ~~Main~~main

floor material, ~~Main~~main wall material, and ~~Main~~main roof material~~)~~ and ~~Maternal~~maternal and child factors (~~Maternal~~such as maternal age at first birth, ~~Sex~~sex of the child~~, Utilization~~), utilization of contraception, ~~Order~~order of birth, ~~Mode~~mode of delivery, duration of breastfeeding and ~~Multiple~~multiple births~~)~~ **(Figure 1)**.

Spatial analysis

The data for spatial analysis was cleaned and merged ~~by~~ using STATA version 16 and ~~excel soft wares~~Microsoft Excel. ArcGIS version 10.8 and saTScan version 9.7 were used for the spatial analysis.

Spatial autocorrelation

Spatial autocorrelation (Global Moran's I) analysis was conducted to ~~see if~~examine whether under-five mortality was ~~measured as~~ dispersed (Moran's I value closer to -1), clustered (Moran's I value closer to 1), or randomly distributed (Moran's I value of 0) in Ethiopia (23).

Spatial interpolation

~~Though all enumeration areas have known~~ The under-five mortality~~,~~ was known in enumerated areas, while in areas that ~~are~~were not selected, the under-five mortality rates were predicted. Spatial interpolation was applied ~~by~~ using the geostatistical ordinary Kriging spatial interpolation technique to predict under-five mortality from ~~a limited number of~~existing sample data points ~~(EAs)~~ to un-sampled areas (24).

Spatial scan statistics

The scan analysis was performed using SaTscan ~~was used~~, based on the Bernoulli test for cases (child is not alive) and controls (child is alive~~) to conduct the scan analysis.~~). The upper limit used was the default maximum spatial cluster size of less than 50% of the population, allowing both small and large clusters to be detected, ~~and~~while clusters that contained more than the maximum limit with the circular shape of the window were avoided(25). Most likely clusters were identified

using p-values and likelihood ratio tests, which is the ratio of the likelihood of the alternative hypothesis (higher activity level inside the window) over the likelihood of the null hypothesis (same activity level inside and outside).

Data management and analysis

We used STATA version 16 and R statistical software version 4.0.5 to analyze the data. ~~Data (KR) was cleaned and~~ A total of 31 variables were ~~kept that were needed~~retained for the analysis. Residents who ~~are~~were not De jure ~~(included in the study if they lasted the night before the study)~~ were excluded which ~~affect~~affected under-five mortality, as they ~~couldn't~~could not respond to most of the socio-demographic and economic characteristics though they ~~can~~could answer the maternal and child characteristics~~, which has~~. This exclusion changed our sample size from ~~5753~~5,753 to ~~5695~~5,695.

The outcome variable was re-coded to (child alive: Yes= 1 and No = 0). ~~The variables were further recording, renaming, labeling, and generating new variables from the original variables was done. These additional four~~ Four community variables were generated by taking the individual variable, calculating their proportion, and dichotomizing them based on their mean or median according to their distribution~~ and merging variables~~.

In the end, we had 29 predictor variables~~ out~~, of which 6 were community-level predictor variables and 23 were individual-level predictor variables.

~~As we have a binary outcome variable (child alive = yes/no), to analyze factors associated with under five mortality, binary logistic regression is appropriate. But based~~ Based on EMDHS~~;~~, respondents ~~with~~in the same cluster ~~show~~showed similar ~~findings~~outcomes or functions at the same level and the data has a hierarchical structure. This ~~makes~~made binary logistic regression not ~~to be used~~the most appropriate as it violates the assumption of independence of the residuals~~; rather~~. Instead, a model ~~which~~that considers clustering effect should be used(8, 11). Multilevel logistic modeling separates the within-cluster effects from the between-cluster effects (26). Therefore, to assess the predictors associated with U5M, a non-weighted multilevel logistic regression model was used.

Bivariable multilevel logistic regression was used ~~for~~to screen each predictor variable ~~to screen out the significant variables (~~for a p-value less than 0.2~~) for~~. Significant variables were included in multivariable multilevel logistic models. Twenty predictor variables (3 of ~~them~~which were community-level predictor variables) ~~passed to~~ were included in the multivariable analysis. In the multivariable analysis, a p-value less than 0.05 was considered a factor associated with U5M.

The first model fitted was the null model (intercept model~~)~~), which ~~contains~~contained the outcome variable only (under-five mortality status) with the cluster number. The intra-cluster correlation (ICC) was used to assess whether there was a random effect. An ICC of 0.130 which ~~means~~meant there ~~is~~was a minimum of 13% under-five mortality ~~which is~~was explained by between~~-~~-cluster ~~difference and around~~differences. We found that 87% of ~~the~~ under-five mortality ~~is~~was explained by within-cluster ~~difference~~differences, which was not negligible. The second model was fitted ~~by~~ using the outcome, the cluster and ~~as a predictor variable~~ the individual~~-~~-level predictor variables only. The probability of U5M was predicted as a function of individual-level predictors.

For the third model the outcome variable, the cluster number, and the community~~-~~-level predictor variables were accounted for. Then the final model was fitted ~~with~~by taking both the individual~~-~~-level and the community~~-~~-level predictor variables~~ taken~~ into account.

The models were compared by using a log-likelihood statistic, where the ~~fitted~~best model ~~had the~~was selected based on smallest ~~-2log * likelihood value that is~~ deviance.

~~Model performance was checked by using residual diagnostic plot in R software package.~~

Ethical consideration

Permission for data access was obtained from a major demographic and health survey through an online request from http://www.dhsprogram.com. The data used for this study were publicly available with no personal identifier.

~~Result~~Results

Socio-demographic ~~Background~~background and maternal and child health factor

~~An overall~~A total of 5,695 live births ~~was encompassed~~were included in ~~the~~this study, of which 2,942 (51.66%) were male, 5,528 (97.07%) were single children, 2,937 (51.57%) were delivered at home and ~~around~~ 6.04% of the children were born via cesarean section (**Table 1**~~)~~).

Out of the total children's mothers, most were married (5,346 ~~(~~, 93.87 %) and 3,135 (55.05%) ~~have~~had no education, only 291 (5.11%) ~~attain~~attained higher education, and ~~Greater than~~ 51.62% ~~of the respondents are~~ were of poor economic status. ~~The majority of the respondents~~ 64.27% ~~are utilizers of~~ had access to improved water ~~and~~; 30.99 % travel more than 30 minutes, and 50.85% travel less than 30 min to get water unlike those who do not have water on-premises. The majority of ~~sex of~~ household ~~head is~~heads were male, 4,561 (80.09~~%)~~%), and the ~~commonest~~most common age of the household head ~~is~~was 35-50 ~~with 45.58%. Background of community factors~~years (**Table 1**~~)~~).

Among the population, the majority ~~of the participants~~ were rural residents ~~with 4389~~4,389 (77.07~~%). And 3457~~%), and 3,457 (60.70%) ~~of~~in the community practiced home delivery. The number of communities that ~~utilize~~used unimproved toilets ~~is 3175~~was 3,175 (55.75%). ~~3391~~3,391 residents of the population (59.54%) ~~are~~were not exposed to the media~~. And the majority of the children reside in rural areas~~ (**Table 2**~~)~~).

Prevalence of under-five mortality

The under-five mortality ~~is~~was 5.9% ~~with its~~ (95% ~~(~~CI 5.4%, 6.6%) and it ~~varies~~varied across regions ranging from 2.13% in Addis Ababa to 9.82% in the Somali region. Out of the total, there were 918 (15.79%) ~~of~~ children who ~~had a history of lower~~died having been breastfed for less than 6 months ~~of breastfeeding died~~ (Table 3~~)~~).

Spatial autocorrelation

The global Moran's index was 0.09, z-score 3.5, and p-value <0.001 suggesting that the spatial distribution under-five mortality ~~is~~was not at random in the 2019 EMDHS (**Figure 2**)(27)~~.~~.

Hotspot analysis

A high proportion of U5M was observed around the regions of Somali and Dire Dawa (**Figure 3**)(27).

Spatial Interpolation

The spatial interpolation or prediction analysis result showed that the under-five mortality ~~ranges~~ranged from 0% to 50%. The ~~high risk~~areas predicted ~~areas~~to have the highest risk were located in Somali, Dire Dawa, Benishangul Gumuz, Gambella, and SNNPR (**Figure 4**)(27).

Spatial scan statistics analysis

As observed in ~~the~~ figure 5, significant clusters were identified in the Somali region. Located within 7.384788 N, 45.908936 E with 612.75 km radius. Children living in the primary cluster were 62% more likely to die before they celebrate their fifth birthday when compared to those who lived outside the window (relative risk (1.62), ~~Likelihood~~likelihood ratio (7.36), and p-value (0.03)) (**Figure 5**)(27).

Factors associated with ~~Under~~under-five mortality

~~By using multilevel logistic regression, after~~After filtering, all variables in bivariable multilevel logistic regression, 4 models were fitted (null model, individual-level model, community-level model, and both individual- and community~~-~~-level model). The ICC was ~~found to be~~ 0.13 ~~(~~with 95% CI: ~~(~~0.08~~,~~ to 0.2~~)~~), which is not negligible. The 4th model was the best-~~fitted~~fitting model with the lowest deviance. When the number of household members ~~increases~~increased by one, the odds of under-five mortality ~~will be~~ decreased by 8.5% (AOR=0.~~09~~92, 95%CI: ~~(~~0.84, 0.99~~)).~~). The odds of under-five death ~~will be~~ decreased by 82.8% (AOR=0.17, 95%CI: 0.14, 0.21) as the number of under-five children in the family ~~increases~~increased by one. Children who ~~have~~were part of multiple births ~~have~~had 14 times higher odds of under-five mortality (AOR=14.4, 95%CI: ~~(~~8.5, 24.3~~))~~) than singletons. Children who were breastfed for less than ~~6months (AOR=5.04, 95%CI: (3.81, 6.67)) have~~6 months had 5 times higher odds of experiencing under-five mortality (AOR=5.04, 95%CI: 3.81, 6.67) than those who ~~have~~were breastfed. People whose main roof ~~is~~was palm/bamboo ~~have~~had 43% (AOR=0.57, 95%CI: ~~(~~0.34, 0.96~~))~~) decreased odds of having under-five deaths in the family than ~~that of~~those with corrugated iron. Under-five children who ~~are~~

~~in~~were the sixth ~~birth order and~~born or more ~~are~~were 2.5 (AOR=2.46, 95%CI: ~~(~~1.49, 4.06~~))~~) times higher odds ~~to have~~of under-five mortality ~~as~~ compared ~~with~~to the second and third ~~birth order~~born. Place of delivery was found to have a significant association with U5M, those who ~~have~~were delivered in institutions ~~have~~had 43% less chance of experiencing U5M (~~-~~AOR=0.57, 95%CI: ~~(~~0.41, 0.81~~)) as~~) compared ~~with~~to those delivered at home. Being a resident of the Somali and Afar region ~~increases~~increased the odds of under-five death by 3.5 (AOR=3.46, 95%CI: ~~(~~1.58, 7.55~~))~~) and 2.5 (AOR=2.54, 95%CI: ~~(~~1.10, 5.85~~))~~) times, respectively~~ as~~, compared ~~with~~to the Tigray region (Table 4).

The residual plot shows that the model ~~performs~~performed adequately, with the Kolmogorov shirnov(KS~~-~~)test being insignificant (p-value = 0.29), and also the deviation was not significant (**Figure 6**).

Discussion

The study mainly focused on the spatial distribution of under-five mortality and on factors that influence it. The findings of this study revealed that the prevalence of under-five mortality was ~~59 per 1000 live births with~~5.9% (95% CI: ~~(54, 66)).~~5.4, 6.6). The multivariable multilevel logistic regression model result showed that family size, number of under-five children, birth order, breastfeeding, multiple birth, region, and institutional delivery were associated with under-five mortality in Ethiopia. The spatial analysis result revealed that Somali, Dire Dawa and Eastern Oromia were high-risk areas for under five mortalities in Ethiopia.

~~The study showed that under-five mortality in Ethiopia is 59 per 1000 live births with (95% CI: (5.4%, 6.6%)). This finding is still below the sustainable development goal target of 25 or low deaths per 1000 live births also it is not consistent with studies conducted in Ethiopia(8, 16), this difference might be due to the survey time of the data used and the sample size.~~

Increased number of ~~households (~~family size~~)~~ resulted in decreased odds of under-five death. ~~It~~This finding is ~~in line~~consistent with ~~a study~~previous studies conducted in Ethiopia(17), ~~and~~ Eastern Nigeria(28)~~. This can be explained by, as the family size increases, the mother of the children will be more experienced in taking good and adequate care of the children and as well as there will be many family members that can care for under-five children.~~

~~As the number of under-five children in a family increase, the odds of under-five mortality will be decreased. This is in agreement with a study in Ghana .~~, and Ghana(29). This ~~is because~~ may be due to the mother ~~will be~~ of the children being more experienced in taking good and ~~she will~~adequate care of the children as the family size increases, in addition to there being more family members that can care for under-five children. Another possible reason might be that the mother may be more experienced and may learn from her previous mistakes if there ~~is one. But there is a contradiction with~~were any. However, this is in contrast to another study ~~result~~conducted in Ethiopia, which demonstrated that the odds of under-five mortality ~~increase~~increased as the number of under-five children ~~increases~~increased(19). This ~~can be justified~~may indicate that , as the number of under-five children increases, it ~~will be~~ may become difficult to fulfill their basic needs, and their family ~~(mother) will be~~too exhausted to look after the children.

~~According to our study results being fourth and fifth or sixth and above born children increase the odds of~~ Birth order had significant effect on under-five mortality. This study indicates that, as order of birth increased, the likelihood of child death also increased. This finding is ~~in line~~consistent with a study conducted in rural parts of Ethiopia (10). ~~But~~However, it ~~is~~does not ~~supported by a~~support another study conducted in Ethiopia which ~~pointed out~~found that being firstborn ~~increases~~increased the odds of under-five mortality ~~by two folds~~twofold when compared to those with five and above birth orders(19). This ~~can~~may be because firstborns are more likely to be hospitalized as a result of congenital malformations and perinatal conditions in their early childhood. ~~But, this~~This changes for firstborn children as they get older, ~~when~~while younger brothers and sisters are more likely to be hospitalized for injuries and avoidable conditions, ~~which shows~~indicating less parental care (30).

~~Breastfeeding less~~Being Breastfed forless than six months ~~increases~~increased the odds of under-five mortality compared with those who were breastfed for six and more months. ~~Which~~This is consistent with a finding in Ethiopia(19). ~~This can be justified by breastfeeding being one of the~~ Breastfeeding is known as a prevention ~~methods~~method for reducing child mortality and ~~it is~~as well as a ~~means of~~ prevention of delayed growth ~~(31)~~(31, 32).

Being multiple birth children ~~increases~~increased the odds of under-five mortality when ~~in comparison~~compared to singleton children. This finding is in line with ~~different~~several studies conducted in Ethiopia at different times, and a study conducted in rural parts of Ethiopia(7, 8, 10,

17, 19). This ~~can~~may be due to multiple births imposing short- and long-term medical complications ~~(32).~~as well as adding increased burden on the mother and the family (33).

Facility or institutional delivery ~~reduces~~reduced the odds of under-five mortality by 43% as compared to home delivery. This finding is consistent with studies conducted in Ethiopia(10, 11) and the Democratic Republic of Congo(14). This is because~~; Home~~ home delivery increases the death of under-five children due to a higher risk of postpartum complications~~ after the intrapartum,~~. These complications include exposure to common vaccine-preventable diseases ~~as a result of an~~if there is absence of vaccination, and ~~less likely to attend~~a lower likelihood of attending prenatal and postpartum visits (11, ~~33~~34).

Field Code Changed

The odds of under-five mortality ~~are~~was higher for residents of Afar and Somali regions ~~as~~ compared to the Tigray region. This coincides among other things, with Somali and Afar ~~have~~regions having the first and second lowest coverage of children with all basic vaccinations. ~~Children under five with symptoms of Acute Respiratory Infection and diarrhea are below the national average.~~ There is serious child malnutrition with the first and second highest prevalence of wasting ~~for~~in the Somali and Afar, respectively~~(34, 35) and~~(35, 36). Additionally, the Somali region was one of the regions that were in the primary cluster from our spatial scan statistics analysis where under-five mortality is more likely to occur. This is not supported by studies conducted in Ethiopia based on the 2011 and 2016 EDHS(10, 11), where other regions ~~are~~were found to have a significant association with under-five mortality. This discrepancy ~~can~~may be a result of the different number of ~~enumeration~~enumerated areas (clusters) used in the different studies, which may have led to different samples, or different statistical models used for analysis ~~(hurdle negative binomial mode1, multilevel negative binomial regression model, and binary logistic regression).~~ .

Conclusion: Under-five mortality in Ethiopia ~~is 59 per 1000 live births which are high and unacceptable as compared to the 2030 agenda for sustainable development aimed at 25 per 1000 live births.~~ was 5.9% of live birth. Being breastfed for more than 6-months~~Breastfeeding more than 6-month~~, twin birth, institutional delivery and high-risk areas of U5M (Somali and dire Dawa) were modifiable risk factors. Therefore, maternal education on the advantage of breastfeeding and institutional delivery is highly recommended. Women who deliver multiple births should be given

special attention. ~~An~~Further, effective ~~strategy~~strategies should be designed for intervention ~~for~~on hot spot areas of U5M~~ that is~~, namely Somali and Dire Dawa.

Strength and limitation of the study

We used EMDHS 2019 data which is more inclusive and generalizable as Enumeration Areas(EAs)are taken from most parts of the country. The larger sample size ~~increases~~increased the power of the study and ~~helps~~helped to make geographical comparisons. As ~~it is~~this was cross-sectional, it does not show a temporal relationship~~ (chicken egg dilemma),~~. Additionally, potentially important variables~~:~~ such as antenatal care ~~follow-up~~visits, postnatal care, and preceding birth interval were excluded from the study due to their ~~highest~~high rate of missing values.

~~Abbreviation~~Abbreviations

AOR: Adjusted Odds Ratio, CI: Confidence Interval, CS: Cesarean Section, EAs: Enumeration Areas, DHS: Demography and Health Survey, EMDHS: Ethiopia Mini Demography and Health Survey, HHs: households, ICC: Intraclass Correlation, KR: kids record, SGD: Sustainable Development Goal, U5M: under-five mortality, U5MR: under-five mortality rate

Declaration

Ethics approval and consent to participate

Ethical clearance was obtained from measure DHS through filling requesting a form for accessing data. The data used in this study are publicly available, aggregated secondary data that ~~hasn't~~does not include any personal identifying information that ~~can~~may be linked to study participants. The confidentiality of data was maintained anonymously.

Availability of data and materials

The datasets used during the current study are publicly available data from measure DHS program found at www.dhsprogram.com~~ ~~.

Funding

The authors received no specific funding for this work.

Acknowledgment

We acknowledge the DHS program for providing us with the dataset. Elizabeth Korevaar reviewed the English language of the manuscript.

Consent for publication

Not applicable as there are no ~~image~~images or other confidentiality-related issues.

Competing interests

The authors declared that they have no competing interests.

Authors' contributions

LGG, TMT, and ZTT conceived the study, data analysis, drafted the manuscript, and critically reviewed the manuscript. ~~Read~~ They read and ~~approve~~approved the final version of the manuscript.

Reference

~~1. Croft T, Marshall A, Allen C. Guide to DHS Statistics. Rockville, Maryland, USA: ICF; 2018. 2018.~~

~~2. Vidak I. OLDER PEOPLE AND SOCIAL MEDIA USAGE. Strategic Approach to Aging Population: Experiences and Challenges: Sveučilište Josipa Jurja Strossmayera u Osijeku, Ekonomski fakultet u Osijeku. p. 325-40.~~

~~3. Woldeamanuel BT, Aga MA. Count models analysis of factors associated with under-five mortality in Ethiopia. Global Pediatric Health. 2021;8:2333794X21989538.~~

~~4. Ezbakhe F, Pérez-Foguet A. Child mortality levels and trends. Demographic Research. 2020;43:1263-96.~~

~~5. Johnston R. Arsenic and the 2030 Agenda for sustainable development. Arsen Res Glob Sustain-Proc 6th Int Congr Arsen Environ AS. 2016;2016:12-4.~~

~~6. Li Z, Hsiao Y, Godwin J, Martin BD, Wakefield J, Clark SJ, et al. Changes in the spatial distribution of the under-five mortality rate: Small-area analysis of 122 DHS surveys in 262 subregions of 35 countries in Africa. PloS one. 2019;14(1):e0210645.~~

~~7. Fenta SM, Fenta HM, Ayenew GM. The best statistical model to estimate predictors of under-five mortality in Ethiopia. Journal of Big Data. 2020;7(1):1-14.~~

8. Worku MG, Teshale AB, Tesema GA. Determinants of under-five mortality in the high mortality regions of Ethiopia: mixed-effect logistic regression analysis. Archives of Public Health. 2021;79(1):1-9.
9. Ayele DG, Zewotir TT, Mwambi HG. Structured additive regression models with spatial correlation to estimate under-five mortality risk factors in Ethiopia. BMC public health. 2015;15(1):1-12.
10. Gebremichael SG, Fenta SM. Under-Five Mortality and Associated Risk Factors in Rural Settings of Ethiopia: Evidences from 2016 Ethiopian Demographic and Health Survey. Advances in Public Health. 2020;2020.
11. Geremew BM, Gelaye KA, Melesse AW, Akalu TY, Baraki AG. Factors Affecting Under-Five Mortality in Ethiopia: A Multilevel Negative Binomial Model. Pediatric Health, Medicine and Therapeutics. 2020;11:525.
12. Ghilagaber G, Antai D, Kandala N-B. Modeling spatial effects on childhood mortality via geo-additive Bayesian discrete-time survival model: A case study from Nigeria. Advanced techniques for modelling maternal and child health in Africa: Springer; 2014. p. 29-48.
13. Singh A, Masquelier B. Continuities and changes in spatial patterns of under-five mortality at the district level in India (1991–2011). International journal of health geographics. 2018;17(1):1-18.
14. Kandala N-B, Mandungu TP, Mbela K, Nzita KP, Kalambayi BB, Kayembe KP, et al. Child mortality in the Democratic Republic of Congo: cross-sectional evidence of the effect of geographic location and prolonged conflict from a national household survey. BMC public health. 2014;14(1):1-9.
15. Anafcheh T, Yaghoubi-Doust M, Mojadam M, Mirkazemi R, Khafaie MA. Temporal and spatial distribution of under-five mortality and factors associated with multiple cases of under-five deaths within a family in the rural area of Khuzestan, Southern Iran. Scientific reports. 2018;8(1):1-8.
16. Ayele DG, Zewotir TT. Childhood mortality spatial distribution in Ethiopia. Journal of Applied Statistics. 2016;43(15):2813-28.
17. Dejene T, Girma E. Social determinants of under-five mortality in Ethiopia: Event history analysis using evidence from Ethiopian Demographic and Health Survey (EDHS). 2013.
18. Fikru C, Getnet M, Shaweno T. Proximate determinants of under-five mortality in Ethiopia: using 2016 nationwide survey data. Pediatric health, medicine and therapeutics. 2019;10:169.
19. Zewudie AT, Gelagay AA, Enyew EF. Determinants of under-five child mortality in Ethiopia: analysis using Ethiopian demographic health survey, 2016. International Journal of Pediatrics. 2020;2020.
20. Abir T, Agho KE, Page AN, Milton AH, Dibley MJ. Risk factors for under-5 mortality: evidence from Bangladesh Demographic and Health Survey, 2004–2011. BMJ open. 2015;5(8):e006722.
21. Pretorius CE, Asare H, Genuneit J, Kruger HS, Ricci C. Impact of breastfeeding on mortality in sub-Saharan Africa: a systematic review, meta-analysis, and cost-evaluation. European Journal of Pediatrics. 2020;179(8):1213-25.
22. Ethiopian Public Health Institute - EPHI, Federal Ministry of Health - FMoH, ICF. Ethiopia Mini Demographic and Health Survey 2019. Addis Ababa, Ethiopia: EPHI/FMoH/ICF; 2021.
23. Fotheringham ASRP. The SAGE handbook of spatial analysis. Los Angeles; London: SAGE Publications; 2009.
24. Bhunia G, Shit P, Maiti R. Comparison of GIS-based interpolation methods for spatial distribution of soil organic carbon (SOC). J Saudi Soc Agric Sci 17: 114–126. 2018.
25. Kulldorff M. A spatial scan statistic. Communications in Statistics-Theory and methods. 1997;26(6):1481-96.
26. Sommet N, Morselli D. Keep calm and learn multilevel logistic modeling: A simplified three-step procedure using stata, R, Mplus, and SPSS. International Review of Social Psychology. 2017;30:203-18.
27. GeoPortal I. OpenAfrica shapfile 2019 [Available from: https://open.africa/dataset/africa-shapefiles.
28. Azuike E, Onyemachi P, Amah C, Okafor K, Anene J. Determinants of under-five mortality in South-Eastern Nigeria. J Community Med Public Health Care. 2019;6:049.

29. Aheto JMK. Predictive model and determinants of under-five child mortality: evidence from the 2014 Ghana demographic and health survey. BMC public health. 2019;19(1):1-10.
30. Julihn A, Soares FC, Hammarfjord U, Hjern A, Dahllöf G. Birth order is associated with caries development in young children: a register-based cohort study. BMC Public Health. 2020;20(1):1-8.
31. Ahmed AH. The International Code of Marketing of Breast-milk Substitutes: Update on the Global Implementation. Journal of Human Lactation. 2020;36(4):803-7.
32. Elster N. Less is more: the risks of multiple births. Fertility and Sterility. 2000;74(4):617-23.
33. Goyal S, Kortsmit K, Cox S, D'Angelo DV, Romero L, Henderson ZT, et al. Prevalence of Home Births and Associated Risk Profile and Maternal Characteristics, 2016–2018. Obstetrics & Gynecology. 2020;136(6):1195-203.
34. Dessalegn M, Ayele M, Hailu Y, Addisu G, Abebe S, Solomon H, et al. Gender inequality and the sexual and reproductive health status of young and older women in the Afar region of Ethiopia. International Journal of Environmental Research and Public Health. 2020;17(12):4592.
35. Oladeji O, Oladeji B, Omer MD, Farah AE, Ameda IM, Gera R, et al. Exploring opportunities to enhance effectiveness of mobile health and nutrition strategy for providing health and nutrition services amongst pastoralists in Somali region, Ethiopia. African Journal of Primary Health Care & Family Medicine. 2021;13(1):1-7.

1. Croft T, Marshall A, Allen C. Guide to DHS Statistics. Rockville, Maryland, USA: ICF; 2018. 2018.
2. Vidak I. OLDER PEOPLE AND SOCIAL MEDIA USAGE. Strategic Approach to Aging Population: Experiences and Challenges: Sveučilište Josipa Jurja Strossmayera u Osijeku, Ekonomski fakultet u Osijeku. p. 325-40.
3. Woldeamanuel BT, Aga MA. Count models analysis of factors associated with under-five mortality in Ethiopia. Global Pediatric Health. 2021;8:2333794X21989538.
4. Ezbakhe F, Pérez-Foguet A. Child mortality levels and trends. Demographic Research. 2020;43:1263-96.
5. Johnston R. Arsenic and the 2030 Agenda for sustainable development. Arsen Res Glob Sustain-Proc 6th Int Congr Arsen Environ AS. 2016;2016:12-4.
6. Li Z, Hsiao Y, Godwin J, Martin BD, Wakefield J, Clark SJ, et al. Changes in the spatial distribution of the under-five mortality rate: Small-area analysis of 122 DHS surveys in 262 subregions of 35 countries in Africa. PloS one. 2019;14(1):e0210645.
7. Fenta SM, Fenta HM, Ayenew GM. The best statistical model to estimate predictors of under-five mortality in Ethiopia. Journal of Big Data. 2020;7(1):1-14.
8. Worku MG, Teshale AB, Tesema GA. Determinants of under-five mortality in the high mortality regions of Ethiopia: mixed-effect logistic regression analysis. Archives of Public Health. 2021;79(1):1-9.
9. Ayele DG, Zewotir TT, Mwambi HG. Structured additive regression models with spatial correlation to estimate under-five mortality risk factors in Ethiopia. BMC public health. 2015;15(1):1-12.
10. Gebremichael SG, Fenta SM. Under-Five Mortality and Associated Risk Factors in Rural Settings of Ethiopia: Evidences from 2016 Ethiopian Demographic and Health Survey. Advances in Public Health. 2020;2020.
11. Geremew BM, Gelaye KA, Melesse AW, Akalu TY, Baraki AG. Factors Affecting Under-Five Mortality in Ethiopia: A Multilevel Negative Binomial Model. Pediatric Health, Medicine and Therapeutics. 2020;11:525.
12. Ghilagaber G, Antai D, Kandala N-B. Modeling spatial effects on childhood mortality via geo-additive Bayesian discrete-time survival model: A case study from Nigeria. Advanced techniques for modelling maternal and child health in Africa: Springer; 2014. p. 29-48.
13. Singh A, Masquelier B. Continuities and changes in spatial patterns of under-five mortality at the district level in India (1991–2011). International journal of health geographics. 2018;17(1):1-18.

14. Kandala N-B, Mandungu TP, Mbela K, Nzita KP, Kalambayi BB, Kayembe KP, et al. Child mortality in the Democratic Republic of Congo: cross-sectional evidence of the effect of geographic location and prolonged conflict from a national household survey. BMC public health. 2014;14(1):1-9.
15. Anafcheh T, Yaghoubi Doust M, Mojadam M, Mirkazemi R, Khafaie MA. Temporal and spatial distribution of under-five mortality and factors associated with multiple cases of under-five deaths within a family in the rural area of Khuzestan, Southern Iran. Scientific reports. 2018;8(1):1-8.
16. Ayele DG, Zewotir TT. Childhood mortality spatial distribution in Ethiopia. Journal of Applied Statistics. 2016;43(15):2813-28.
17. Dejene T, Girma E. Social determinants of under-five mortality in Ethiopia: Event history analysis using evidence from Ethiopian Demographic and Health Survey (EDHS). 2013.
18. Fikru C, Getnet M, Shaweno T. Proximate determinants of under-five mortality in Ethiopia: using 2016 nationwide survey data. Pediatric health, medicine and therapeutics. 2019;10:169.
19. Zewudie AT, Gelagay AA, Enyew EF. Determinants of under-five child mortality in Ethiopia: analysis using Ethiopian demographic health survey, 2016. International Journal of Pediatrics. 2020;2020.
20. Abir T, Agho KE, Page AN, Milton AH, Dibley MJ. Risk factors for under-5 mortality: evidence from Bangladesh Demographic and Health Survey, 2004–2011. BMJ open. 2015;5(8):e006722.
21. Pretorius CE, Asare H, Genuneit J, Kruger HS, Ricci C. Impact of breastfeeding on mortality in sub-Saharan Africa: a systematic review, meta-analysis, and cost-evaluation. European Journal of Pediatrics. 2020;179(8):1213-25.
22. Ethiopian Public Health Institute - EPHI, Federal Ministry of Health - FMoH, ICF. Ethiopia Mini Demographic and Health Survey 2019. Addis Ababa, Ethiopia: EPHI/FMoH/ICF; 2021.
23. Fotheringham ASRP. The SAGE handbook of spatial analysis. Los Angeles; London: SAGE Publications; 2009.
24. Bhunia G, Shit P, Maiti R. Comparison of GIS-based interpolation methods for spatial distribution of soil organic carbon (SOC). J Saudi Soc Agric Sci 17: 114–126. 2018.
25. Kulldorff M. A spatial scan statistic. Communications in Statistics-Theory and methods. 1997;26(6):1481-96.
26. Sommet N, Morselli D. Keep calm and learn multilevel logistic modeling: A simplified three-step procedure using stata, R, Mplus, and SPSS. International Review of Social Psychology. 2017;30:203-18.
27. GeoPortal I. OpenAfrica shapfile 2019 [Available from: https://open.africa/dataset/africa-shapefiles.
28. Azuike E, Onyemachi P, Amah C, Okafor K, Anene J. Determinants of under-five mortality in South-Eastern Nigeria. J Community Med Public Health Care. 2019;6:049.
29. Aheto JMK. Predictive model and determinants of under-five child mortality: evidence from the 2014 Ghana demographic and health survey. BMC public health. 2019;19(1):1-10.
30. Julihn A, Soares FC, Hammarfjord U, Hjern A, Dahllöf G. Birth order is associated with caries development in young children: a register-based cohort study. BMC Public Health. 2020;20(1):1-8.
31. Ahmed AH. The International Code of Marketing of Breast-milk Substitutes: Update on the Global Implementation. Journal of Human Lactation. 2020;36(4):803-7.
32. WHO U. Global nutrition targets 2025: breastfeeding policy brief (WHO/NMH/NHD14. 7). Geneva: World Health Organization. 2014.
33. Elster N. Less is more: the risks of multiple births. Fertility and Sterility. 2000;74(4):617-23.
34. Goyal S, Kortsmit K, Cox S, D'Angelo DV, Romero L, Henderson ZT, et al. Prevalence of Home Births and Associated Risk Profile and Maternal Characteristics, 2016–2018. Obstetrics & Gynecology. 2020;136(6):1195-203.
35. Dessalegn M, Ayele M, Hailu Y, Addisu G, Abebe S, Solomon H, et al. Gender inequality and the sexual and reproductive health status of young and older women in the Afar region of Ethiopia. International Journal of Environmental Research and Public Health. 2020;17(12):4592.

36. Oladeji O, Oladeji B, Omer MD, Farah AE, Ameda IM, Gera R, et al. Exploring opportunities to enhance effectiveness of mobile health and nutrition strategy for providing health and nutrition services amongst pastoralists in Somali region, Ethiopia. African Journal of Primary Health Care & Family Medicine. 2021;13(1):1-7.

List of Figures

Figure 1~~:-~~: conceptual framework of under-five mortality in Ethiopia 2019(27).

Figure 2~~:-~~: Spatial autocorrelation of under-five mortality in Ethiopia 2019(27).

Figure 3~~:-~~: Hotspot analysis of under-five mortality in Ethiopia 2019(27).

Figure 4~~:-~~: Empirical Bayesian Kriging prediction map of under-five mortality in Ethiopia 2019(27).

Figure 5~~:-~~: Spatial scan statistics of under-five mortality in Ethiopia 2019(27).

Figure 6~~:-~~: residual diagnostic plot for the final model of under-five mortality in Ethiopia 2019.

Table 1 Sociodemographic, maternal, and child health factors

Sociodemographic, Maternal and child health factors		Frequency	Percentage
Age of the mother at first birth	Less than 18	3,024	53.10%
	19 ~~upto~~to 34	2,650	46.53%
	35 and more	21	0.37%
Sex of child	Male	2,942	51.66%
	Female	2,753	48.34%
Current Contraceptive utilization	~~not use~~Not used	3,875	68.04%
	~~use~~Used	1,820	31.96%
Multiple births	Singleton	5,528	97.07%
	Multiple	167	2.93%

Formatted Table

Formatted: Left

Formatted: Left

Formatted: Left

Formatted: Left

Formatted: Left

Formatted: Left

Formatted: Left

Formatted: Left

Formatted: Left

Birth order	1st order	1,228	21.56%
	2nd and 3rd order	1,883	33.06%
	4th and 5th order	1,298	22.79%
	6th order ~~-~~+	1,286	22.58%
Place where the mother delivered	~~home~~Home delivery	2,937	51.57%
	Facility delivery	2,758	48.43%
Delivery by cesarean section	No	5,351	93.96%
	~~yes~~Yes	344	6.04%
Months of Breastfeeding~~Breastfeed~~	Less than ~~6month~~6 months	918	16.12%
	6 ~~month~~months and ~~grater~~more	4,777	83.88%
Sex of household head	Male	4,561	80.09%
	Female	1,134	19.91%
Age of household head (years?)	~~less~~Less than ~~34~~ 35	2,479	43.53%
	35 ~~up~~ to 50	2,596	45.58%
	50 and more	620	10.89%
Marital Status	Married	5,346	93.87%
	Unmarried	349	6.13%
Highest educational level	No education	3,135	55.05%
	Primary	1,802	31.64%
	Secondary	467	8.20%
	Higher	291	5.11%
Wealth index	Poor	2,940	51.62%
	Middle	797	13.99%
	Rich	1,958	34.38%
Water source	Improved	3,660	64.27%
	Unimproved	2,035	35.73%

Time to fetch water	~~more~~More than 30 min	1,765	30.99%
	~~less~~Less than 30 min	2,896	50.85%
	On premises	1,034	18.16%
Type of latrine used	Improved	1,164	20.44%
	Not improved	4,531	79.56%
Household has electricity	No	4,073	71.52%
	Yes	1,622	28.48%
Cooking fuel in use	Clean fuel	432	7.59%
	Solid fuel	5,263	92.41%
Type of floor of the house	Earth/sand/dung	4,384	76.98%
	Wood	49	0.86%
	Cement	799	14.03%
	Carpet	463	8.13 -%
Type of the house wall	Cane/Palm/Trunk/Reed	1,261	22.14%
	Bamboo/wood	3,531	62.00%
	Cement/stone with lime/bricks/covered adobe	903	15.86%
Type of the house roof	Thatch	1,488	26.13%
	Palm/bam	995	17.47%
	Cement	198	3.48%
	~~corrugated~~Corrugated iron	3,014	52.92 %

Table 2 Community-level ~~factors~~variable

Community level ~~factors~~ variable		frequency	Percentage
Place of residence	Urban	1,306	22.93%
	Rural	4,389	77.07%
Community place of delivery	Home delivery	3,457	60.70%
	Facility delivery	2,238	39.30%
Community toilet utilization	Improved	2,520	44.25%
	Not improved	3,175	55.75%
Community wealth status	Poor	2,776	48.74%
	Rich	2,919	51.26%
Community media exposure	Not exposed to media	3,391	59.54%
	Exposed to media	2,304	40.46%

Table 3 Under-five mortality among predictors

Characteristics		Is the child alive?		total
		Yes	~~No~~	
		~~-~~5357(94.06%)	~~338~~No338(5.94%)	
Is child twin?	Yes	123(2.16%)	44(0.77%)	167(2.93%)
	No	5234(91.9%)	294(5.16%)	5,528-(97.06%)
Region	Tigray	436(7.65%)	14(0.24%)	450(7.90%)
	Afar	616(10.81%)	35(0.612%)	651(11.43%)
	Amhara	478(8.39%)	23(0.40%)	501-(8.79%)
	Oromia	674(11.83%)	41(0.72%)	715-(12.55%)

	Somali	580(10.18%)	57(1.00%)	637-(11.18%)
	Benishangul	477(8.37%)	45(0.79%)	522-(9.17%)
	SNNPR	631(11.08%)	26(0.46%)	657-(11.54%)
	Gambella	409(7.18%)	35(0.61%)	444-(7.79%)
	Harari	408(7.16%)	29(0.51%)	437(7.67%)
	Addis Ababa	281(4.93%)	6(0.11%)	287-(5.04%)
	Dire Dawa	367(6.44%)	27(0.47%)	394(6.92%)
Breastfeeding	Less than 6 months	773(13.57%)	145(2.55%)	918(16.12%)
	6 months and more	4,584(80.49%)	193(3.39%)	4,777-(83.88%)
Birth order	1st order	1,142(20.05%)	86(1.52%)	1,228(21.56%)
	2-3rd order	1,793(31.48%)	90(1.56%)	1,883-(33.06%)
	4-5th order	1,231(21.62%)	67(1.18%)	1,298-(22.79%)
	6th + order	1,191(20.91%)	95(1.67%)	1,286-(22.58%)
Place of delivery	Home delivery	2,733(47.99%)	204(3.58%)	2,937-(51.57%)
	Facility delivery	2,624(46.06%)	134(2.35%)	2,758(48.43%)

Formatted: Tab stops: 0.71", Centered

Table 4 Multivariable multilevel logistic regression analysis of both individual and community-level factors associated with under-five mortality in Ethiopia, EMDHS 2019.

Variable	Null model	Model2 AOR (95%CI)	Model3 AOR(95%CI)	Model4 AOR(95%CI)
Education				
No education		1		1
Primary		1.14 (0.83,1.56)		1.14 (0.83,1.58)
Secondary		0. .59 (0.31, 1.13)		0.58(0.30,1.12)
Higher		0. .65(0. .28, 1.55)		0.60(0.25,1.44)
Household members		0.922(0. 85, 1.01)		0.92(0.83,0.99) *
Number of under-five children		0.17 (0.14, 0 .22)		0.17(0.14, 0.21) ***
Sex				
Male		1		1
female		0 .83(0 .64, 1.07)		0.84(0.64,1.09)
Mode of delivery by C/S				
No		1		1
Yes		1.36 (0. 79, 2.35)		1.37(0.79,2.39)
Preceding birth order				
1st		1		1
2-3rd		1.32(0.92, 1.91)		1.34(0.93,1.94)

Formatted: Justified
Formatted Table
Formatted: Justified, Space After: 5.6 pt
Formatted: Justified, Space After: 5.6 pt
Formatted: Justified, Space After: 5.6 pt
Formatted: Left
Formatted: Left
Formatted: Left
Formatted: Space After: 6.05 pt, Tab stops: 0.4", Centered + 1.15", Right
Formatted: Left
Formatted: Space After: 5.75 pt
Formatted: Space After: 5.95 pt, Tab stops: 1.15", Right
Formatted: Font: 10 pt
Formatted: Font: 10 pt
Formatted: Space After: 5.65 pt
Formatted: Space After: 5.65 pt
Formatted: Space After: 5.95 pt, Tab stops: 1.38", Right
Formatted: Left
Formatted: Left
Formatted: Space After: 6.05 pt, Tab stops: 0.45", Centered + 1.15", Right
Formatted: Left
Formatted: Left
Formatted: Space After: 6.05 pt, Tab stops: 0.58", Centered + 1.15", Right
Formatted: Left
Formatted: Left

4-5th		1.52(0.97, 2.39)		1.51(0.96,2.38)
6+		2.44(1.48, 4.02)		2.46(1.49,4.06) ***
Time to water				
>30min		1		1
<30min		0.77(0.56, 1.04)		0.89(0.64,1.23)
On-premises		0.66(0.38, 1.14)		0.76(0.43,1.34)
Cooking fuel				
Clean fuel		1		1
Solid fuel		1.27(0.62, 2.58)		0.93(0.45,1.94)
Age at 1st birth				
19-34		1		1
<=18		0.91(0.70, 1.20)		0.90(0.68,1.18)
>=35		2.58(0.55, 12.01)		2.72(0.55,13.59)
Breastfeeding				
< 6 months		1		1
>= 6 months		5.09(3.85, 6.72)		5.05(3.82,6.67) ***
Place of delivery				
Home delivery		1		1
Facility delivery		0.54 (0.40,0.75)		0.57(0.41,0.81) **
Main floor				
earth/sand/dung		1		1
Wood		1.71(0.53, 5.52)		2.14(0.66,6.95)
Cement		0.89(0.50, 1.59)		0.86(0.48,1.58)
Carpet		1.56(0.85, 2.86)		1.28(0.67,2.46)
Main wall				
bamboo/wood		1		1

Formatted: Left
Formatted Table
Formatted: Left
Formatted: Space After: 5.6 pt
Formatted: Left
Formatted: Left
Formatted: Left
Formatted: Left
Formatted: Left
Formatted: Left
Formatted: Left
Formatted: Left
Formatted: Space After: 5.75 pt
Formatted: Left
Formatted: Left
Formatted: Space After: 5.6 pt
Formatted: Left
Formatted: Left
Formatted: Left
Formatted: Left
Formatted: Left
Formatted: Left
Formatted: Left
Formatted: Left

cane/palm/trunks/re ed		1.42(0~~———~~.97, 2.07)		1.06(0.70,1.58)
cement/stone		1.53(0.89,2.61)		1.34(0.76,2.34)
Main roof				
Corrugated -iron		1		
thatch		1.25(0.87,1.79)		1.14(0.77,1.67)
palm/bamboo		0.71(0.44,1.15)		0.57(0.34,0.96)*
calamine/cement		1.14(0.54,2.44)		1.04(0.47,2.29)
Contraceptive utilization				
No		1		1
Yes		0.72(0.53,0.98)		0.82(0.60,1.13)
Marital status				
Currently Married		1		1
Currently Unmarried		1.19(0.74,1.92)		1.22(0.75,1.97)
Multiple Twin child				
No		1		1
Yes		15.63(9.33,26.~~2~~ ~~0~~20)		14.39(8.51,24.33~~)*~~ ~~**~~)***
Region				
Tigray			1	1
Afar			1.60(0.76,3.~~4~~ ~~1~~41)	2.55(1.10,5.85)*
Amhara			1.47(0.68,3.~~1~~ ~~9~~19)	0.97(0.44,2.15)
Oromia			1.73(0.84,3.~~5~~ ~~5~~55)	1.52(0.73,3.15)
Somali			2.86(1.39,5.~~8~~ ~~9~~89)	3.46(1.59,7.55)**

Formatted: Left
Formatted Table
Formatted: Space After: 6.1 pt, Tab stops: 0.22", Centered + 0.95", Centered
Formatted: Left
Formatted: Left
Formatted: Left
Formatted: Left
Formatted: Left
Formatted: Left
Formatted: Left
Formatted: Left
Formatted: Left, Right: 0.08", Space After: 5.6 pt
Formatted: Left
Formatted: Left
Formatted: Space After: 5.75 pt
Formatted: Space After: 5.75 pt
Formatted: Left
Formatted: Left
Formatted: Space After: 5.6 pt
Formatted: Left
Formatted: Space After: 5.6 pt
Formatted: Left
Formatted: Space After: 5.75 pt
Formatted: Left
Formatted: Space After: 5.75 pt

Benishangul gumuz			2.74(1.32,5.~~68~~68)	1.95(0.91,4.20)
SNNPR			1.17(.55,2.47)	0.94(0.44,2.03)
Gambella			2.71(1.29,5.~~69~~69)	1.94(0.90,4.21)
Harari			2.37(1.12,5.~~04~~04)	1.95(0.87,4.36)
Addis Ababa			0.71(0.25,2.~~03~~03)	0.64(0.21,2.02)
Dire Dawa			2.18(1.01,4.~~70~~70)	2.02(0.91,4.50)
Community delivery place				
Home delivery			1	1
Facility delivery			0.75(0.52, 1.09)	0.94(0.62,1.43)
Community media				
Not exposed			1	1
exposed			1.09(0.75,1.~~58~~58)	0.94(0.64,1.40)
Intercept	0.49(0.30,0.~~81~~81)	0.22(0.08,0.63)	0.35(0.19,0.~~64~~64)	0.16(0.04,0.57)
ICC	0.130	0.036	0.091	0.025
Log-likelihood	-1263.56	-918.15	-1247.40	-903.72

*** P-value<0.001, ** p-value<0.01, * p-value<0.05

Formatted: Space After: 5.75 pt
Formatted: Left
Formatted: Space After: 5.75 pt
Formatted: Left
Formatted: Space After: 5.75 pt
Formatted: Left
Formatted: Space After: 5.6 pt
Formatted: Left
Formatted: Space After: 5.65 pt
Formatted: Left
Formatted: Space After: 5.6 pt
Formatted: Left
Formatted: Left
Formatted: Space After: 5.6 pt
Formatted: Left
Formatted: Left
Formatted: Space After: 5.75 pt
Formatted: Font: 10 pt
Formatted: Space After: 5.75 pt
Formatted: Font: 10 pt
Formatted: Font: 10 pt
Formatted: Space After: 5.75 pt

PLOS ONE

Point by point response for editors/reviewers' comments

The manuscript title "Geographic variation and factors associated with under-five mortality in Ethiopia. A spatial and multilevel analysis of Ethiopian Mini Demographic and Health Surveys 2019"

Manuscript number: PONE-D-21-20589R2

Dear editor/reviewer,

I would like to thank you for these constructive, building, and improvable comments on this manuscript that would improve the substance and content of the manuscript. We considered each comment and clarification question of reviewers on the manuscript thoroughly. My point-by-point responses for each comment and question are described in detail on the following pages. Further, the details of changes were shown by track changes in the supplementary document attached.

Reviewer comment and authors response

Reviewer #1:

General comments:

Reviewer's comment: - THIS IS A GOOD STUDY BUT IS TERRIBLY WRITTEN! THE ONLY WAY IT WILL BE PUBLISHED IS THAT THE AUTHORS LOOK IN DETAIL AT ALL THE IMPRECISIONS AND BADLY WRITTEN CONTENT. ONLY IF THEY RE-WRITE ENTIRELY WITH AN ENGLISH-SPEAKING PERSON (WHOSE FIRST LANGUAGE IS ENGLISH OR IS VERY PROFFICIENT IN ENGLISH), THIS MANUSCRIPT WILL BE PUBLISHED. OTHERWISE, UNFORTUNATELY, IT CANNOT BE PUBLISHED! PLEASE PAY CLOSE ATTENTION TO ALL THE WRITING

Authors response: Thank you very much for you very important comment specially the language of the manuscript and recommendation to give our manuscript for language editor to English speaking person. We accept your comment and gave our manuscript for English speaking person (Elizabeth Korevaar). We acknowledge her support in our revised manuscript. She gave us extensive language edition and we corrected it in the revised version of the manuscript.

Reviewer's comment: The comment of the reviewer was given on the PDF part of the manuscript with detail comments.

Authors response: - We accept all comments and corrected it accordingly.
