# Supplementary material for: Geographic variation and factors associated with under-five mortality in Ethiopia. A spatial and multilevel analysis of Ethiopian mini demographic and health survey 2019
Source: PLoS One. 2022 Oct 13;17(10):e0275586. doi: 10.1371/journal.pone.0275586 (PMC9560495; doi:10.1371/journal.pone.0275586)
Supplement: Attachment — Submitted filename: Point by point response for U5M.docx [file pone.0275586.s005.docx]

**PLOS ONE**

**Point by point response for editors/reviewers’ comments**

**The manuscript title** “Geographic variation and factors associated with under-five mortality in Ethiopia. A spatial and multilevel analysis of Ethiopian Mini Demographic and Health Surveys 2019”

**Manuscript number:** PONE-D-21-20589R2

Dear editor/reviewer,

I would like to thank you for these constructive, building, and improvable comments on this manuscript that would improve the substance and content of the manuscript. We considered each comment and clarification question of reviewers on the manuscript thoroughly. My point-by-point responses for each comment and question are described in detail on the following pages. Further, the details of changes were shown by track changes in the supplementary document attached.

**Reviewer comment and authors response**

**Reviewer #1**:

General comments:

**Reviewer's comment: -** THIS IS A GOOD STUDY BUT IS TERRIBLY WRITTEN! THE ONLY WAY IT WILL BE PUBLISHED IS THAT THE AUTHORS LOOK IN DETAIL AT ALL THE IMPRECISIONS AND BADLY WRITTEN CONTENT. ONLY IF THEY RE-WRITE ENTIRELY WITH AN ENGLISH-SPEAKING PERSON (WHOSE FIRST LANGUAGE IS ENGLISH OR IS VERY PROFFICIENT IN ENGLISH), THIS MANUSCRIPT WILL BE PUBLISHED. OTHERWISE, UNFORTUNATELY, IT CANNOT BE PUBLISHED! PLEASE PAY CLOSE ATTENTION TO ALL THE WRITING

**Authors response**: Thank you very much for you very important comment specially the language of the manuscript and recommendation to give our manuscript for language editor to English speaking person. We accept your comment and gave our manuscript for English speaking person (Elizabeth Korevaar). We acknowledge her support in our revised manuscript. She gave us extensive language edition and we corrected it in the revised version of the manuscript.

**Reviewer's comment:** The comment of the reviewer was given on the PDF part of the manuscript with detail comments.

**Authors response**: - We accept all comments and corrected it accordingly.
