# Supplementary material for: Geographic variation and factors associated with under-five mortality in Ethiopia. A spatial and multilevel analysis of Ethiopian mini demographic and health survey 2019
Source: PLoS One. 2022 Oct 13;17(10):e0275586. doi: 10.1371/journal.pone.0275586 (PMC9560495; doi:10.1371/journal.pone.0275586)
Supplement: Attachment — Submitted filename: Point by point response for U5M.docx [file pone.0275586.s007.docx]

**PLOS ONE**

**Point by point response for editors/reviewers’ comments**

**The manuscript title** “Geographic variation and factors associated with under-five mortality in Ethiopia. A spatial and multilevel analysis of Ethiopian Mini Demographic and Health Surveys 2019”

**Manuscript number:** PONE-D-21-20589R2

Dear editor,

I would like to thank you for these constructive, building, and improvable comments on this manuscript that would improve the substance and content of the manuscript. We considered each of your recommendation of language edition. Further, the details of changes were shown by track changes in the supplementary document attached.
